# Approximated prediction of genomic selection accuracy when reference and candidate populations are related

**DOI:** 10.1186/s12711-016-0183-3

**Published:** 2016-03-03

**Authors:** Jean-Michel Elsen

**Affiliations:** GenPhySE (Génétique, Physiologie et Systèmes d’Elevage), INRA, 31326 Castanet-Tolosan, France; Animal Genetics and Breeding Unit, University of New England, Armidale, Australia

## Abstract

**Background:**

Genomic selection is still to be evaluated and optimized in many species. Mathematical modeling of selection schemes prior to their implementation is a classical and useful tool for that purpose. These models include formalization of a number of entities including the precision of the estimated breeding value. To model genomic selection schemes, equations that predict this reliability as a function of factors such as the size of the reference population, its diversity, its genetic distance from the group of selection candidates genotyped, number of markers and strength of linkage disequilibrium are needed. The present paper aims at exploring new approximations of this reliability.

**Results:**

Two alternative approximations are proposed for the estimation of the reliability of genomic estimated breeding values (GEBV) in the case of non-independence between candidate and reference populations. Both were derived from the Taylor series heuristic approach suggested by Goddard in 2009. A numerical exploration of their properties showed that the series were not equivalent in terms of convergence to the exact reliability, that the approximations may overestimate the precision of GEBV and that they converged towards their theoretical expectations. Formulae derived for these approximations were simple to handle in the case of independent markers. A few parameters that describe the markers’ genotypic variability (allele frequencies, linkage disequilibrium) can be estimated from genomic data corresponding to the population of interest or after making assumptions about their distribution. When markers are not in linkage equilibrium, replacing the real number of markers and QTL by the “effective number of independent loci”, as proposed earlier is a practical solution. In this paper, we considered an alternative, *i.e.* an “equivalent number of independent loci” which would give a GEBV reliability for unrelated individuals by considering a sub-set of independent markers that is identical to the reliability obtained by considering the full set of markers.

**Conclusions:**

This paper is a further step towards the development of deterministic models that describe breeding plans based on the use of genomic information. Such deterministic models carry low computational burden, which allows design optimization through intensive numerical exploration.

**Electronic supplementary material:**

The online version of this article (doi:10.1186/s12711-016-0183-3) contains supplementary material, which is available to authorized users.

## Background

The effectiveness of genomic selection comes from the possibility of predicting breeding values on un-phenotyped and young animals [1]. Genomic selection promised and proved to be extremely efficient and beneficial for dairy cattle (e.g. [2–7]), but debate continues for other species and production sectors (e.g. [8–12]). A key criterion to decide whether or not selection schemes (also referred to here as breeding plans) should include genomic information is the reliability of the genomic predictor. It was clearly shown that this reliability depends on the structure of the reference population and on the characteristics of the marker set used. The size of this reference population, its diversity, the genetic distance between the reference and the group of selection candidates genotyped, the number of markers, and the degree or strength of the linkage disequilibrium are the main factors that influence this reliability [13–23].

An extensive literature exists on the mathematical modeling of selection schemes prior to their implementation, in order, for instance, to optimize their design, or to evaluate the usefulness of new technologies such as embryo transfer, sperm selection, DNA markers and others (e.g. [24] for a review). These models account for factors such as selection intensities and maintenance or loss of genetic variability. Among these parameters, the precision of breeding value estimates is central. To model genomic selection schemes, equations that predict this reliability as a function of the factors cited above are needed (e.g. [6, 25, 26]).

The quantitative influence of these factors (size of the reference population, its diversity, etc.) was assessed by simulation studies [18–21, 27, 28]. An equation that predicts the reliability of genomic evaluation in the very simple situation of independent quantitative trait loci (QTL), that are perfectly marked by single nucleotide polymorphisms (SNPs) and populations (reference and candidates) of unrelated individuals was derived [13]. This approach was extended to the case when only a part of the genetic variability is imperfectly marked by SNPs [16, 19], and the situation of non-independence between reference and candidate populations was explored [17]. It was demonstrated that genomic information captures historical linkage disequilibrium, short-term linkage between QTL and markers and additive relationships between reference and candidate individuals, the equation of the reliability accounting for these three phenomena being derived in a very simple case of one QTL marked by a single SNP [22].

A Taylor expansion of a matrix inverse involved in the reliability formula was suggested [18], which led to the algebraic development of an approximation. This approximation seems to work well in the simple situation but lacks generality. In this paper, an alternative approximation is proposed, opening a way to include non-independence between reference and candidate populations, and between markers.

After a formalization of the genomic selection context, the principles that underlie these approximations are presented and their properties are compared by using a simple example. Then, the new approximation is derived when reference and candidate animals are related. This is illustrated by some numerical examples. Finally, the extension to the linkage disequilibrium situation is described.

## Methods

### General framework

Although the prediction equations derived below were based on a number of simplifying assumptions, it is important to first draw a complete description of the biological framework, as a basis to subsequently simplify the discussion.

The SNP effects are estimated in a reference population, P_r_, comprising *n*_*r*_ individuals. The genomic estimated breeding values (GEBV) are calculated for a population of candidates for selection and used in breeding, P_c_, comprising *n*_*c*_ individuals.

Let ${\mathbf{\mathcal{P}}} = \left( {{\mathbf{\mathcal{P}}}_{r} ,{\mathbf{\mathcal{P}}}_{c} } \right)$ the population structure (including pedigree relationships between individuals and marker allele frequencies, but not including genotypes and phenotypes).

Individuals are characterized by their genotypes at *n*_*M*_ markers (observed) and at *n*_*Q*_ QTL (unknown). Alleles will be noted *A*_*m*_ and *B*_*m*_ for the marker *m* and *A*_*q*_ and *B*_*q*_ for the QTL *q*. Let *a*_*tim*_ ∊ {0, 1, 2} and *a*_*tiq*_ ∊ {0, 1, 2} be the numbers of *B*_*m*_ (and respectively, *B*_*q*_) alleles that an individual *i* from population P_t_ (P_r_ or P_c_) carries at marker *m* (respectively, QTL *q*). Let *p*_*tm*_ and *p*_*tq*_ be the frequencies of alleles *B*_*m*_ and *B*_*q*_ in P_t_.

Genotypic values will be assigned to the different markers and QTL genotypes. Following [18], genotypes will be coded as *x*_*tim*_ = *a*_*tim*_ − 2*p*_*tm*_ and *w*_*tiq*_ = *a*_*tiq*_ − 2*p*_*tq*_. Different codifications can be proposed [15]. In particular, as described for instance in [29], genotypic values may be standardized, *i.e.x*_*tim*_ = (*a*_*tim*_ − 2*p*_*tm*_)/*σ*_*tm*_ and *w*_*tiq*_ = (*a*_*tiq*_ −2*p*_*tq*_)/*σ*_*tq*_, with variances *σ*_*tm*_^2^ = 2*p*_*tm*_(1 − *p*_*tm*_) and *σ*_*tq*_^2^ = 2*p*_*tq*_(1 − *p*_*tq*_). Most of the following developments are given with the first codification here, and the results with the second codification are described in a specific section.

These genotypic values are assembled in matrices **X** (dim (**X**) = (*n*_*r*_ + *n*_*c*_) × *n*_*M*_) and **W** (dim (**W**) = (*n*_*r*_ + *n*_*c*_) × *n*_*Q*_). Sub-matrices corresponding to sub-populations will be noted in the following way: **X**^′^ = (**X**_**r**_^′^, **X**_**c**_^′^) and **W**^′^ = (**W**_**r**_^′^, **W**_**c**_^′^).

The genetic model assumes additivity of QTL effects. The additive genetic value of an individual is described as $g_{ti} = \sum\nolimits_{q = 1}^{{n_{Q} }} {w_{tiq} \alpha_{q} }$ and, in general, ${\mathbf{ g = W\alpha }}$. The phenotypic values when observed are **y** = **g** + ***ɛ***.

A statistical model describes the performances in the reference population as random variables for which the expectations are linear combinations of SNP effects: $y_{ri} = \sum\nolimits_{m = 1}^{{n_{S} }} {X_{rim} \beta_{m} + e_{ri} }$ and, in general, **y** = **Xβ** + **e**.

In these models, the SNP (or QTL) effects may be considered as fixed, or random. Since the number of SNPs is much bigger than the number of individuals (*n*_*M*_ ≫ *n*_*r*_) the second solution is generally chosen in the statistical model (but not always see [1, 13]).

In the random model, a distribution ${\mathcal{L}}\left( {{\varvec{\uptheta}}_{{\upbeta }} ,{\mathbf{V}}_{{\upbeta }} } \right)$ (respectively ${\mathcal{L}}\left( {{\varvec{\uptheta}}_{\upalpha} ,{\mathbf{V}}_{\upalpha} } \right)$) of the SNP (respectively QTL) effects is assumed, with **θ**_**β**_ (respectively, **θ**_α_) being the vector of expectations and **V**_β_ (respectively, **V**_α_) being the matrix of variances. For a full description of the variability, the **V**_β_ and **V**_α_ matrices are each subdivided into four blocks corresponding to the reference and candidate populations and their covariances. Covariances between the **α** and **β** vectors have also to be considered. Most generally, the SNP (QTL) effects are supposed *i.i.d.* giving **V**_β_ = **I**σ_β_^2^ (**V**_α_ = **I**σ_α_^2^). The interpretation of these QTL effects is nicely debated in Gianola et al. [30]. In the frequentist view, we simply have to imagine that QTL effects are randomly sampled from a distribution with a σ_α_^2^ variance. In the Bayesian context, the prior variability of the SNP effects was most generally described as heteroskedastic or even coming from mixtures of SNPs with or without an effect on the trait.

The expectations ${\varvec{\uptheta}}_{{\upbeta }} \left( {{\varvec{\uptheta}}_{{\upalpha }} } \right)$ are generally assumed equal to zero, but when information about population history is available (in particular, when we know it is a mixed population), non-zero values should be considered.

The vector **q** = **Xβ** is a quantity similar but not equal to the genetic value **g**. Its element *q*_*ti*_ is the molecular score of individual *i* in population *t*. This vector may be segmented in two parts: ${\mathbf{q^{\prime}}} = \left( {{\mathbf{q^{\prime}_{{{r}}}}} ,{\mathbf{q^{\prime}_{{\mathbf{c}}} }}} \right)$.

Since the variances may be defined within a population, we have $v\left( {{\mathbf{q}}_{{\mathbf{r}}} |{\mathbf{X}}} \right) = {\mathbf{X}}_{{\mathbf{r}}} {\mathbf{V}}_{{{\bf\upbeta r}}} {\mathbf{X}}^{\prime}_{{\mathbf{r}}}$, and *v*(**q**_**c**_|**X**) = **X**_**c**_**V**_βc_**X**_**c**_^′^. The residual variance is *v*(**e**) = **I***σ*_*e*_^2^. Assuming that the distribution of marker effects is centered (${\varvec{\uptheta}}_{{\varvec{\upbeta}}} = {\bf 0}$) and *i.i.d.* (${\mathbf{V}}_{{{\upbeta r}}} = {\mathbf{I}}\sigma_{{{\upbeta r}}}^{2} \, {\text{and}} \,{\mathbf{V}}_{{{\upbeta c}}} = {\mathbf{I}}\sigma_{{{\upbeta c}}}^{2}$), and extending Gianola et al. [30], we have $v\left( {q_{ri} } \right) = {\text{E}}_{{\mathbf{X}}} \left[ {v\left( {q_{\text{ri}} |{\mathbf{X}}} \right)} \right] = \sigma_{\beta r}^{2} \sum\nolimits_{m} {2p_{mr} \left( {1 - p_{mr} } \right)} = \sigma_{\beta r}^{2} \sum\nolimits_{m} {\sigma_{mr}^{2} = \sigma_{\beta r}^{2} \tau_{r} }$ in the reference population, and *v*(*q*_*ci*_) = *σ*_βc_^2^*τ*_*c*_ in the candidate population. Assuming that the distribution of the marker effects and genotypes are the same in P_r_ and P_c_, *i.e.*$p_{rm} = p_{cm} = p_{m} ,  p_{rq} = p_{cq} = p_{q}$, thus *τ*_*r*_ = *τ*_*C*_ = *τ* and *σ*_*βr*_^2^ = *σ*_*βc*_^2^ = *σ*_*β*_^2^, we define *σ*_*q*_^2^ = *τσ*_β_^2^. Thus, $v\left( {{\mathbf{q}}|{\mathbf{X}}} \right) = \frac{1}{\tau }{\mathbf{XX^{\prime}}}\sigma_{{q}}^{2}$. These equations hold even if the markers are in linkage disequilibrium (LD) as shown in Eq. A2 from Gianola et al. [30].

We note *σ*^2^ as the total phenotypic variance, *i.e.σ*^2^ = *σ*_*q*_^2^ + *σ*_*e*_^2^, and *ν*^2^ as the proportion of this variance explained by the molecular score $\left( {\nu^{2} = \frac{{\sigma_{q}^{2} }}{{\sigma^{2} }}} \right)$. The ratio $\frac{{\sigma_{q}^{2} }}{{\sigma_{e}^{2} }}$ will be noted *γ*.

The SNP effects **β** may be estimated in different ways. The genomic best linear unbiased prediction (BLUP) will only be considered here, with ${\hat{\varvec{\upbeta} }} = {\text{cov}}\left( {{\varvec{\upbeta}},{\mathbf{y}}} \right){\text{var}}\left( {\mathbf{y}} \right)^{ - 1} {\mathbf{y}}$. Classically, this equation becomes ${\hat{\varvec{\upbeta }}} = \sigma_{{{\upbeta }}}^{2} {\mathbf{X}}^{\prime}_{{\mathbf{r}}} \left[ {\sigma_{{{\upbeta }}}^{2} \left( {{\mathbf{X}}_{{\mathbf{r}}} {\mathbf{X}}^{\prime}_{{\mathbf{r}}} + {\mathbf{I}}{{\uplambda }}_{{{\upbeta }}} } \right)} \right]^{ - 1} {\mathbf{y}} = \left( {{\mathbf{X}}^{\prime}_{{\mathbf{r}}} {\mathbf{X}}_{{\mathbf{r}}} + {\mathbf{I}}{{\uplambda }}_{{{\upbeta }}} } \right)^{ - 1} {\mathbf{X}}^{\prime}_{{\mathbf{r}} }{\mathbf{y}}$ with λ_β_ = σ_e_^2^/σ_β_^2^. The linear combination $\hat{\varvec{q}}_{\varvec{c}} = {\mathbf{X}}_{\varvec{c}} {\hat{\varvec{\upbeta}}}$ is the GBLUP vector for candidates in P_c_. It must be emphasized that these estimations and predictions are conditional on the genotypic structures defined by **X** (**X**_**r**_ and ${\mathbf{X}}_{\varvec{c}}$).

Given **X**, the reliability of the GBLUP is $r^{2} \left( {g_{ci} ,\hat{q}_{ci} |{\mathbf{X}}} \right) = \frac{{cov^{2} \left( {g_{ci} ,\hat{q}_{ci} |{\mathbf{X}}} \right)}}{{v\left( {g_{ci} |{\mathbf{X}}} \right)v\left( {\hat{q}_{ci} |{\mathbf{X}}} \right)}}$.

In [16], the reliability is described (Eq. 6 in [16]) as $r\left( {g_{ci} ,\hat{q}_{ci} } \right) = r\left( {g_{ci} ,q_{ci} } \right) \times r\left( {q_{ci} ,\hat{q}_{ci} } \right)$, by ignoring the conditioning on **X**. In Goddard et al. [18], the reliability is described as $r_{{g_{ci} ,\hat{q}_{ci} }}^{2} = \frac{{v\left( {\hat{q}_{ci} } \right)}}{{v\left( {g_{ci} } \right)}} = \frac{{v\left( {q_{ci} } \right)}}{{v\left( {g_{ci} } \right)}}\frac{{v\left( {\hat{q}_{ci} } \right)}}{{v\left( {q_{ci} } \right)}}$. In this formulation, $\frac{{v\left( {q_{ci} } \right)}}{{v\left( {g_{ci} } \right)}}$ is the proportion of the genetic variance explained by the markers and $\frac{{v\left( {\hat{q}_{ci} } \right)}}{{v\left( {q_{ci} } \right)}}$ is the accuracy of estimated marker effects. This is similar to the ${\text{qr}}_{{\hat{Q}}}$ reported by Dekkers et al. [25]. All these reliability formulae are approximations since $cov^{2} \left( {g_{ci} ,\hat{q}_{ci} } \right) = cov^{2} \left( {\sum w_{ciq} \alpha_{q} ,\sum x_{cis} \hat{\beta }_{s} } \right) \ne v\left( {\hat{q}_{ci} } \right) = v\left( {\sum x_{cis} \hat{\beta }_{s} } \right)$, in general.

### Situation analyzed in this paper

In the following, ignoring the difficulty that was mentioned above, we will assume $r^{2} \left( {q_{\text{ci}} ,\hat{q}_{ci} |{\mathbf{X}}} \right) = \frac{{cov^{2} \left( {q_{ci} ,\hat{q}_{ci} |{\mathbf{X}}} \right)}}{{v\left( {q_{ci} |{\mathbf{X}}} \right)v\left( {\hat{q}_{ci} |{\mathbf{X}}} \right)}} = \frac{{v\left( {\hat{q}_{ci} |{\mathbf{X}}} \right)}}{{v\left( {q_{ci} |{\mathbf{X}}  } \right)}}$. We are interested in a single candidate in $\varvec{P}_{\varvec{c}}$ with a **x**_**c**_ vector of marker genotypes.

Formulae were simplified in two ways. (1) the *i* index of the candidate was omitted in the following developments: the genetic value of the candidate is noted *q*_*c*_, estimated by $\hat{q}_{c} = cov\left( {q_{c} ,{\mathbf{y}}} \right)v\left( {\mathbf{y}} \right)^{ - 1} {\mathbf{y}}$, and its precision is $r^{2} \left( {q_{\text{c}} ,\hat{q}_{c} |{\mathbf{X}}} \right) = \frac{{{\text{v}}(\hat{q}_{c} |{\mathbf{X}})}}{{{\text{v}}\left( {q_{\text{c}} |{\mathbf{X}}} \right)}}$, with $v\left( {q_{\text{c}} |{\mathbf{X}}} \right) = \sigma_{{{\upbeta }}}^{2} {\mathbf{x}}_{{\mathbf{c}}} {\mathbf{x}}^{\prime}_{{\mathbf{c}}}$ and $v\left( {\hat{q}_{c} |{\mathbf{X}}} \right) = \sigma_{{{\upbeta }}}^{2} {\mathbf{x}}_{{\mathbf{c}}} {\mathbf{X}}^{\prime}_{{\mathbf{r}}} \left( {{\mathbf{X}}_{{\mathbf{r}}} {\mathbf{X}}^{\prime}_{{\mathbf{r}}} + {\mathbf{I}}{{\uplambda }}_{{{\upbeta }}} } \right)^{ - 1} {\mathbf{X}}_{{\mathbf{r}}} {\mathbf{x}}^{\prime}_{{\mathbf{c}}}$ (where ${\mathbf{x}}_{{\mathbf{c}}}$ is a row vector); (2) the *r* indices of reference individuals were most often omitted, which resulted in *y*_*i*_ for their phenotypes and *q*_*i*_ for their molecular scores.

In fact, our objective was to estimate the expectation of this precision across the variation domain of **X**_**r**_ and **x**_**c**_ given the pedigree structure $\left( {{\mathbf{\mathcal{P}}}_{\varvec{r}} ,{\mathbf{\mathcal{P}}}_{\varvec{c}} } \right):\;{\text{E}}_{{\mathbf{X}}} \left[ {r^{2} \left( {q_{\text{c}} ,\hat{q}_{c} |{\mathbf{X}}} \right)|{\mathbf{\mathcal{P}}}} \right]$. It will be noted $E\left[ {r_{{q_{\text{c}} ,\hat{q}_{\text{c}} }}^{2} } \right]$.

The following approximation was made: ${\text{E}}\left[ {r_{{q_{\text{c}} ,\hat{q}_{\text{c}} }}^{2} } \right] = \frac{{{\text{E}}_{{\mathbf{X}}} \left[ {{\text{v}}(\hat{q}_{c} |{\mathbf{X}})} \right]}}{{{\text{E}}_{{\mathbf{X}}} \left[ {{\text{v}}\left( {q_{\text{c}} |{\mathbf{X}}} \right)} \right]}} = \frac{{{\text{E}}\left[ {{\text{v}}\left( {\hat{q}_{c} } \right)} \right]}}{{{\text{E}}\left[ {{\text{v}}\left( {q_{\text{c}} } \right)} \right]}}$.

Let **A** be the pedigree relationship matrix between individuals in ${\mathbf{\mathcal{P}}}$. Its blocks are $$ {\mathbf{A}} = \left( {\begin{array}{*{20}c} {{\text{a}}_{\text{cc}} } & {{\mathbf{A}}_{{{\mathbf{cr}}}} } \\ {{\mathbf{A}}_{{{\mathbf{rc}}}} } & {{\mathbf{A}}_{{{\mathbf{rr}}}} } \\ \end{array} } \right) $$. Let $$ {\mathbf{G}}^{ *} = {\mathbf{XX^{\prime}}} = \left( {\begin{array}{*{20}c} {{\mathbf{x}}_{{\mathbf{c}}} {\mathbf{x}}^{\prime}} & {{\mathbf{x}}_{{\mathbf{c}}} {\mathbf{X}}^{\prime}_{{\mathbf{r}}} } \\ {{\mathbf{X}}_{{\mathbf{r}}} {\mathbf{x}}^{\prime}_{{\mathbf{c}}} } & {{\mathbf{X}}_{{\mathbf{r}}} {\mathbf{X}}^{\prime}_{{\mathbf{r}}} } \\ \end{array} } \right) $$, which results in ${\mathbf{V}} = \frac{1}{\tau }{\mathbf{G}}^{ *} \sigma_{q}^{2} + {\mathbf{I}}\sigma_{e}^{2}$. It must be noted that the *σ*_*e*_^2^ term in the diagonal of the **V** submatrix corresponding to the candidate population is artificial since candidates are not phenotyped.

We have E[G*] = **A***τ*. The limits of this equality will be discussed below. As indicated above, the denominator of the expected reliability ${\text{E}}_{{\mathbf{X}}} \left[ {v\left( {q_{\text{c}} |{\mathbf{X}}} \right)} \right]$, is *τσ*_β_^2^ = *σ*_*q*_^2^. Approximating ${\text{E}}\left[ {v\left( {\hat{q}_{c} } \right)} \right]$ by E[*cov*(*q*_*c*_, **y**)]*E*[*v*(**y**)]^−1^*E*[*cov*(**y**, *q*_*c*_)] is useless because it makes an oversimplification of the relationships between the reference and the candidate population: it considers separately the marginal distributions of **x**_**c**_**X**_**r**_^′^ and (**X**_**r**_**X**_**r**_^′^ + **I**λ_β_)^−1^, while these random matrices are correlated. Estimating directly E[*cov*(*q*_*c*_, **y**)*v*(**y**)^−1^*cov*(**y**, *q*_*c*_)] seems impossible in the general case. The approach of Goddard et al. [18] avoids this difficulty, *i.e.* the variance $v\left( {\hat{q}_{c} |{\mathbf{X}}} \right) = \sigma_{{{\upbeta }}}^{2} {\mathbf{x}}_{{\mathbf{c}}} {\mathbf{x}}^{\prime}_{{\mathbf{c}}} + \sigma_{e}^{2} - \frac{1}{{\left\{ {{\mathbf{V}}^{ - 1} } \right\}_{{\varvec{cc}}} }}$, and **V**^−1^ is approximated by a second degree Taylor expansion (${\mathbf{V}}^{ - 1} \sim {\varvec{\Lambda}}\left( {\mathbf{X}} \right)$), giving $v\left( {\hat{q}_{c} |{\mathbf{X}}} \right)\sim \sigma_{{{\upbeta }}}^{2} {\mathbf{x}}_{{\mathbf{c}}} {\mathbf{x}}^{\prime}_{{\mathbf{c}}} + \sigma_{e}^{2} - \frac{1}{{{\varvec{\Lambda}}_{\text{cc}} \left( {{\mathbf{x}}_{{\mathbf{c}}} ,{\mathbf{X}}_{{\mathbf{r}}} } \right)}}$.

### Alternative approximations of the reliability

#### Extension of Goddard’s formula

In their “heuristic approximation for V^*−1^”, Goddard et al. [18] considered the situation where unrelated individuals are included in the reference and candidate populations, that is E[G^*^] = **I***τ* and ${\mathbf{G}}^{ *} = {\mathbf{I}}\tau + {\mathbf{E}}$, with **E**, a “noise” matrix centered on the null matrix ${\bf 0}.$ A direct extension of their development would be the following. The matrix ${\mathbf{V}} = \frac{1}{\tau }{\mathbf{G}}^{ *} \sigma_{q}^{2} + {\mathbf{I}}\sigma_{e}^{2}$ can be written as: 

**V** = *σ*_*e*_^2^(**I** + **A***γ*)[**I** + **D***γ*],

with $$ {\mathbf{D}} = \left( {{\mathbf{I}} + {\mathbf{A}}\gamma } \right)^{ - 1} \left( {\frac{1}{\tau }{\mathbf{G}}^{ *} - {\mathbf{A}}} \right) = {\mathbf{T}}\left( {\frac{1}{\tau }{\mathbf{G}}^{ *} - {\mathbf{A}}} \right) $$,

and $\gamma = \frac{{\sigma_{q}^{2} }}{{\sigma_{e}^{2} }}$. Thus, ${\mathbf{V}}^{ - 1} = \frac{1}{{\sigma_{e}^{2} }}\left[ {{\mathbf{I}} + {\mathbf{D}}\gamma } \right]^{ - 1} {\mathbf{T}}$. The inverse matrix [**I** + **D***γ*]^−1^ will be approximated using a Taylor series. It must be emphasized that the Taylor series **I** − **D***γ* + (**D***γ*)^2^ − (**D***γ*)^3^ + ··· converges towards [**I** + **D***γ*]^−1^ only if the highest Eigen value of **D***γ* is smaller than 1, *i.e.* if $\left( {{\mathbf{D}}\gamma } \right)^{\text{t}} \to {\bf 0}$ when t → ∞.

The second order approximation of **V**^−1^ is equal to $\frac{1}{{\sigma_{e}^{2} }}\left( {{\mathbf{I}} - {\mathbf{D}}\gamma + {\mathbf{D}}^{2} \gamma^{2} } \right){\mathbf{T}}$. As E[**D**] = **0** and ${\text{E}}\left[ {{\mathbf{D}}^{2} } \right] = {\mathbf{T}}\left( {\frac{1}{{\tau^{2} }}{\text{E}}\left[ {{\mathbf{G}}^{ *} {\mathbf{TG}}^{ *} } \right] - {\mathbf{ATA}}  } \right),$ its expectation ${\text{E}}\left[ {\varvec{\Lambda}} \right] = \frac{1}{{\sigma_{e}^{2} }}\left( {{\mathbf{I}} - {\text{E}}\left[ {\mathbf{D}} \right]\gamma + {\text{E}}\left[ {{\mathbf{D}}^{2} } \right]\gamma^{2} } \right){\mathbf{T}}$*i.e.*${\text{E}}\left[ {\varvec{\Lambda}} \right] = \frac{1}{{\sigma_{e}^{2} }}\left( {{\mathbf{I}} - \gamma^{2} {\mathbf{TATA}} + \frac{{\gamma^{2} }}{{\tau^{2} }}{\text{E}}\left[ {{\mathbf{TG}}^{ *} {\mathbf{TG}}^{ *} } \right]} \right){\mathbf{T}}$.

Finally, the reliability of the candidate GBLUP is approximated by:

1 $$ {\tilde{\text{E}}}\left[ {r_{{q_{\text{c}} ,\hat{q}_{\text{c}} }}^{2} } \right]\sim \frac{1}{{\nu^{2} }} - \frac{1}{{\gamma {\mathbf{T}}_{\text{cc}} - \gamma^{3} \left\{ {{\mathbf{TATAT}}} \right\}_{\text{cc}} + \frac{{\gamma^{3} }}{{\tau^{2} }}\left\{ {{\mathbf{T}}{\text{E}}\left[ {{\mathbf{G}}^{ *} {\mathbf{TG}}^{ *} } \right]{\mathbf{T}}} \right\}_{\text{cc}} }} . $$

A difficulty with this approximation comes from the **T** term. As an example, consider a reference population composed of *n*_*r*_ half-sibs of the candidate, ${\mathbf{T}} = {{\upxi }}{\mathbf{I}} + {{\uppsi  }}{\mathbf{J}}$ with ${{\upxi }} = \frac{4}{4 + 3\gamma }$. As ${\mathbf{T}}^{\varvec{t}} = {{\upxi }}^{\varvec{t}} {\mathbf{I}} + \left[ {n_{r}^{t} {{\upxi }}^{\varvec{t}} + \cdots } \right]{\mathbf{J}}$, the **J** coefficient will tend to ∞ as soon as $n_{r} {{\upxi }} = \frac{{4n_{r} }}{4 + 3\gamma } > 1$, a very realistic situation. Thus, the convergence of the Taylor series will be a balance between the increase of ${\mathbf{T}}^{\varvec{t}}$ and decrease of $\left[ {{\mathbf{D}}\gamma } \right]^{\varvec{t}}$.

#### Another approximation of the reliability

##### Principle

Using the classical matrix inversion lemma, the variance $v\left( {\hat{q}_{{\text{c}}} |{\mathbf{x}}_{{\mathbf{c}}} ,{\mathbf{X}}_{{\mathbf{r}}} } \right) = \sigma _{\upbeta }^{2} {\mathbf{x}}_{{\mathbf{c}}} {\mathbf{X}}^{\prime}_{{\mathbf{r}}} \left( {{\mathbf{X}}_{{\mathbf{r}}} {\mathbf{X}}^{\prime}_{{\mathbf{r}}}  + {\mathbf{I}}\uplambda _{\upbeta } } \right)^{{ - 1}} {\mathbf{X}}_{{\mathbf{r}}} {\mathbf{x}}^{\prime}_{{\text{c}}}$ may also be defined as ${\boldsymbol{v}}\left( {\hat{q}_{c} |{\mathbf{x}}_{{\mathbf{c}}} ,{\mathbf{X}}_{{\mathbf{r}}} } \right) = \sigma_{{{\upbeta }}}^{2} {\mathbf{x}}_{{\mathbf{c}}} {\mathbf{x}}^{\prime}_{{\mathbf{c}}} - \sigma_{\text{e}}^{2} {\mathbf{x}}_{{\mathbf{c}}} \left( {{\mathbf{X}}^{\prime}_{{\mathbf{r}}} {\mathbf{X}}_{{\mathbf{r}}} + {\mathbf{I}}{{\uplambda }}_{{{\upbeta }}} } \right)^{ - 1} {\mathbf{x}}^{\prime}_{{\mathbf{c}}}$.

${\mathbf{X}}^{\prime}_{{\mathbf{r}}} {\mathbf{X}}_{{\mathbf{r}}}$ is a very large matrix $\left( {n_{M} \times n_{M} } \right)$ that describes the LD between markers: its elements tend to be smaller when they are more distant from the diagonal.

Elements of E[**X**_**r**_^**′**^**X**_**r**_] are the following: ${\text{E}}\left[ {{\mathbf{X}}^{\prime}_{{\mathbf{r}}} {\mathbf{X}}_{{\mathbf{r}}} } \right]_{\text{ml}} = {\text{E}}\left[ {\mathop {\sum\nolimits_{\text{i}} {\left( {a_{im} - 2p_{m} } \right)\left( {a_{il} - 2p_{l} } \right)} }\limits_{{}} } \right] = 2n_{r} \Updelta_{ml},$ with $\Updelta_{ml}$ the LD between loci *m* and *l*.

E[**X**_**r**_^′^**X**_**r**_]_mm_ = E[ ∑ _i_(*a*_*im*_ − 2*p*_*m*_)^2^] = *n*_*r*_*σ*_m_^2^. Let ${\mathbf{C}} = {\mathbf{I}}{{\uplambda }}_{{{\upbeta }}} + n_{r} {\text{diag}}\left[ {\sigma_{1}^{2} , \ldots ,\sigma_{{n_{M} }}^{2} } \right]$, the **X**_**r**_^′^**X**_**r**_ + **I**λ_β_ matrix may be written as:

${\mathbf{X_r^\prime}} {\mathbf{X}}_{{\mathbf{r}}} + {\mathbf{I}}{{\uplambda }}_{{{\upbeta }}} = \left[ {\left( {{\mathbf{X}}^{\prime}_{{\mathbf{r}}} {\mathbf{X}}_{{\mathbf{r}}} - n_{r} {\text{diag}}\left[ {\sigma_{1}^{2} , \ldots ,\sigma_{{n_{M} }}^{2} } \right]} \right){\mathbf{C}}^{ - 1} + {\mathbf{I}}} \right]{\mathbf{C}}$, which results in:

**X**_**r**_^′^**X**_**r**_ + **I**λ_β_ = [**I** + **B**]**C**.

The convergence of the Taylor series **I** − **B** + **B**^2^ − **B**^3^ + ··· to (**I** + **B**)^−1^ depends on the structure of the **B** matrix, which varies depending on the sample. However, we can examine the case of its expectation E[**B**].

E[**B**]_*mm*_ = 0 and ${\text{E}}\left[ {\mathbf{B}} \right]_{ml} = \frac{{2n_{r} \Updelta_{ml} }}{{{{\uplambda }}_{{{\upbeta }}} + n_{r} \sigma_{\text{l}}^{2} }}$. The ratio λ_β_ is proportional to the number of markers (${{\uplambda }}_{{{\upbeta }}} = n_{M} \frac{{\bar{\sigma }_{\text{m}}^{2} \sigma_{e}^{2} }}{{\sigma_{\text{q}}^{2} }})$ and dominates the denominator when *n*_*M*_ ≫ *n*_*r*_. The (m, l) term in E[**B**]^2^, *i.e.*${\text{E}}\left[ {\mathbf{B}} \right]_{{\varvec{ml}}}^{2} = \sum\nolimits_{k} {\frac{{4n_{r}^{2} \Updelta_{mk} \Updelta_{kl} }}{{\left( {{{\uplambda }}_{{{\upbeta }}} + n_{r} \sigma_{\text{k}}^{2} } \right)\left( {{{\uplambda }}_{{{\upbeta }}} + n_{r} \sigma_{\text{l}}^{2} } \right)}}}$, is of order $\frac{1}{{n_{M} }}$. Thus, we expect the Taylor series to converge to (**I** + E[**B**])^−1^.

##### First order approximation

At the first order, $v\left({\hat{q}_{c} |{\mathbf{x}}_{{\mathbf{c}}} ,{\mathbf{X}}_{{\mathbf{r}}} } \right) \sim \sigma_{{{\upbeta }}}^{2} {\mathbf{x}}_{{\mathbf{c}}} {\mathbf{x}}^{\prime}_{{\mathbf{c}}} - \sigma_{\text{e}}^{2} {\mathbf{x}}_{{\mathbf{c}}} {\mathbf{C}}^{ - 1} \left( {{\mathbf{I}} - {\mathbf{B}}} \right){\mathbf{x}}^{\prime}_{{\mathbf{c}}}$ and the expectation of the reliability of the candidate GBLUP is approximated by $\tilde{\tilde{\text{E}}}\left[ r_{q_{{\text{c}}} ,\hat{q}_{{\text{c}}} }^{2}  \right] = 1 - \frac{{\sigma _{{\text{e}}}^{2} {\text{E}}\left[ {{\mathbf{x}}_{{\mathbf{c}}} {\mathbf{C}}^{{ - 1}} \left( {{\mathbf{I}} - {\mathbf{B}}} \right){\mathbf{x}}^{\prime}_{{\mathbf{c}}} } \right]}}{{\sigma _{\upbeta }^{2} {\text{E}}\left[ {{\mathbf{x}}_{{\mathbf{c}}} {\mathbf{x}}^{\prime}_{{\mathbf{c}}} } \right]}}$.

$$  \begin{aligned} {\mathbf{x}}_{{\mathbf{c}}} {\mathbf{C}}^{-1}
\left( {{\mathbf{I}} -
{\mathbf{B}}}\right){\mathbf{x^{\prime}_{{\mathbf{c}}}}} &= \mathop
\sum \limits_{m} \frac{{x_{cm}^{2}}}{{\lambda_{\beta } + n_{r}
\sigma_{m}^{2} }} + n_{r} \mathop \sum \limits_{m}
\frac{{\sigma_{m}^{2} x_{cm}^{2} }}{{\left( {\lambda_{\beta } +
n_{r} \sigma_{m}^{2} } \right)^{2} }} \\
&\quad-  {\mathbf{x}}_{{\mathbf{c}}} {\mathbf{C}}^{ - 1} \left(
{\mathbf{X_r^\prime}}{\mathbf{X}}_{{\mathbf{r}}} 
\right){\mathbf{C}}^{ - 1} {\mathbf{x^{\prime}_{{\mathbf{c}}}}}
 .\end{aligned}$$

Using $$ {\mathbf{x}}_{{\mathbf{c}}} {\mathbf{C}}^{ - 1} {\mathbf{X}}^{\prime}_{{\mathbf{r}}} = \left({\begin{array}{*{20}l} {\sum\nolimits_{\text{m}} {\frac{{{\text{x}}_{\text{cm}} {\text{X}}_{{{\text{r}}1{\text{m}}}} }}{{{{\uplambda }}_{{{\upbeta }}} + {\text{n}}_{\text{r}} {{\sigma }}_{\text{m}}^{2} }}} } & \cdots & {\sum\nolimits_{\text{m}} {\frac{{{\text{x}}_{\text{cm}} {\text{X}}_{{{\text{rn}}_{\text{r}} {\text{m}}}} }}{{{{\uplambda }}_{{{\upbeta }}} + {\text{n}}_{\text{r}} {{\sigma }}_{\text{m}}^{2} }}} } \\ \end{array} }\right) $$, the last term is: $\sum\nolimits_{i} {\left( {\mathop \sum \nolimits_{m} \frac{{x_{cm} {\text{X}}_{rim} }}{{\lambda_{\beta } + n_{r} \sigma_{m}^{2} }}} \right)^{2} } .$ Finally, the expectation is:

2 $$ \begin{aligned} {{\tilde{\tilde{E}}}}\left[ {r_{{q_{\text{c}} ,\hat{q}_{\text{c}} }}^{2} } \right] =& \,1 - \frac{{\lambda_{\beta } }}{\tau }\left\{ {\sum\nolimits_{m} {\left[ {\frac{{\sigma_{m}^{2} }}{{\lambda_{\beta } + n_{r} \sigma_{m}^{2} }} + \frac{{n_{r} \sigma_{m}^{4} }}{{\left( {\lambda_{\beta } + n_{r} \sigma_{m}^{2} } \right)^{2} }}} \right]} } \right. \hfill \\ & \left. { - \sum\nolimits_{i} {\sum\nolimits_{m} {\left[ {\frac{{{\text{E}}\left[ {x_{cm}^{2} X_{rim}^{2} } \right]}}{{\left( {\lambda_{\beta } + n_{r} \sigma_{m}^{2} } \right)^{2} }} + \sum\nolimits_{l \ne m} {\frac{{{\text{E}}\left[ {x_{cm} X_{rim} x_{cl} X_{ril} } \right]}}{{\left( {\lambda_{\beta } + n_{r} \sigma_{m}^{2} } \right)\left( {\lambda_{\beta } + n_{r} \sigma_{l}^{2} } \right)}}} } \right]} } } \right\} \hfill \\. \end{aligned} $$

## Results

### Application in the case of independent markers

This situation either assumes low density marker information, or corresponds to the idea of an effective number of loci that was developed by Goddard [16, 31]. In the first case, the proportion of the genetic variance explained by the markers $\frac{{v\left( {q_{ci} } \right)}}{{v\left( {g_{ci} } \right)}}$ is small and this quantity should be considered when estimating the genomic precision.

#### First approximation

Using the notation **X**^′^ = (**x**_**c**_^′^, **X**_**r**_^′^), the (*i*, *j*) element of ${\mathbf{G}}^{ *} {\mathbf{TG}}^{ *}$ is: $\left\{ {{\mathbf{XX^{\prime}TXX^{\prime}}}} \right\}_{\text{ij}} = \sum\nolimits_{l} {\sum\nolimits_{k} {t_{kl} \left( {\sum\nolimits_{m} {X_{im} X_{km} } } \right)\left( {\sum\nolimits_{m} {X_{jm} X_{lm} } } \right)} }.$ Thus, elements of E[**XX**^′^**TXX**^′^] will involve expectations of fourth level moments of *X*_*im*_ within *m* joint distributions: E[${\text{E}}\left[ {X_{im}^{2} X_{jm} X_{km} } \right], {\text{E}}\left[ {X_{im}^{2} X_{jm}^{2} } \right], {\text{E}}\left[ {X_{im}^{3} X_{jm} } \right]$*X*_*im*_*X*_*jm*_*X*_*km*_*X*_*lm*_], and E[*X*_*im*_^4^]. Defining and $\tau _{2}  = \sum _{m} [2p_{m} (1 - p_{m} )]^{2}$*a*_*ij*_ the coancestry coefficient between individuals *i* and *j*, we found that [See Additional file 1]: $\left\{ {{\text{E}}\left[ {{\mathbf{XX^{\prime}TXX^{\prime}}}} \right]} \right\}_{\text{ij}} = \sum\nolimits_{l} {\sum\nolimits_{k} {t_{kl} \left( {\frac{1}{2}\tau \alpha_{ijkl}^{1111} - \frac{1}{4}\tau_{2} \gamma_{ijkl}^{1111} + 4a_{ik} a_{jl} \left[ {\tau^{2} - \tau_{2} } \right]} \right)} },$ where parameters $\alpha_{ij \cdots K}^{{d_{i} d_{j} \cdots d_{K} }}$ and $\gamma_{ij \cdots K}^{{d_{i} d_{j} \cdots d_{K} }}$ are functions of the probabilities of the identity states between gametes of $ij \cdots K$ individuals at marker *m* (Table 1). In the summations above, when individuals are repeated (e.g. *i* = *j*), the corresponding exponents are summed (e.g. α_*iiil*_^1111^ = *α*_*il*_^31^).The resulting *X*_*im*_ moments are in Table 2.

**Table 1:** Coefficients describing the genotypes’ distributions moments when using the relation ${\text{E}}\left[ {X_{im}^{{d_{i} }} X_{jm}^{{d_{j} }} \cdots X_{Km}^{{d_{K} }} } \right] = p_{m} \left( {1 - p_{m} } \right)\alpha_{ij \cdots K}^{{d_{i} d_{j} \cdots d_{K} }} - \left[ {p_{m} \left( {1 - p_{m} } \right)} \right]^{2} \gamma_{ij \cdots K}^{{d_{i} d_{j} \cdots d_{K} }}$ from Additional file 1

E[*X*_*im*_]	$\alpha_{i}^{1} = 0$ $\gamma_{i}^{1} = 0$
E[*X*_*im*_^2^]	$\alpha_{i}^{2} = 2$ $\gamma_{i}^{2} = 0$
E[*X*_*im*_^4^]	$\alpha_{i}^{4} = 2$ $\gamma_{i}^{4} = 0$
E[*X*_*im*_ *X*_*jm*_]	$\alpha_{ij}^{11} = 4\delta_{ 1} + 2\left[ {\delta_{ 2} + \delta_{ 3} + \delta_{ 4} + \delta_{ 5} + \delta_{ 9} + \delta_{ 1 2} } \right] + \delta_{ 10} + \delta_{ 1 1} + \delta_{ 1 3} + \delta_{ 1 4} = 4a_{ij}$ $\gamma_{ij}^{ 1 1} = 0$
E[*X*_*im*_ *X*_*jm*_^3^]	$\alpha_{ij}^{13} = 16\delta_{1} + 2\left( {\delta_{2} + \delta_{3} ) + 8(\delta_{4} + \delta_{5} } \right) + 2\left( {\delta_{9} + \delta_{12} } \right) + \delta_{10} + \delta_{11} + \delta_{13} + \delta_{14}$ $\gamma_{ij}^{13} = 2 4\delta_{ 1} + 1 2\left( {\delta_{ 4} + \delta_{ 5} } \right)$
E[*X*_*im*_^2^ *X*_*jm*_^2^]	$\alpha_{ij}^{22} = 1 6\delta_{ 1} + 4\left( {\delta_{ 2} + \delta_{ 3} + \delta_{ 4} + \delta_{ 5} } \right) + 2\left( {\delta_{ 9} + \delta_{ 1 2} } \right) + \delta_{ 10} + \delta_{ 1 1} + \delta_{ 1 3} + \delta_{ 1 4}$ $\gamma_{ij}^{22} = 4 8\delta_{ 1} + 8\left( {\delta_{ 2} + \delta_{ 3} + \delta_{ 4} + \delta_{ 5} } \right) - 4\delta_{ 1 5} - 1 6\delta_{ 6} - 8\left( {\delta_{ 7} + \delta_{ 8} } \right)$

*δ*_*s*_ are the 15 classical identity states probabilities between two individuals [33–35]

Coefficients of expectations ${\text{E}}\left[ {X_{i} X_{j} X_{k}^{2} } \right]$ and E[*X*_*i*_ *X*_*j*_ *X*_*k*_ *X*_*l*_] involve IBD status between three or four different individuals and are explained in Additional file 1

**Table 2:** Moments of genotypes’ distributions depending on genotype codification

Expectations	Genotype codification	
	*x*_*tim*_ = *a*_*tim*_ − 2*p*_*tm*_	*x*_*tim*_ = (*a*_*tim*_ − 2*p*_*tm*_)/*σ*_*tm*_
E[*X*_*im*_]	0	0
E[*X*_*im*_^2^]	*σ*_m_^2^	1
E[*X*_*im*_^4^]	*σ*_m_^2^	1/*σ*_m_^2^
E[*X*_*im*_ *X*_*jm*_]	2*a*_*ij*_ *σ*_m_^2^	2*a*_*ij*_
E[*X*_*im*_ *X*_*jm*_^3^]	$\frac{1}{2}\alpha_{ij}^{13} \sigma_{\text{m}}^{2} - \frac{1}{4}\gamma_{ij}^{13} \sigma_{\text{m}}^{4}$	$\frac{1}{2}\alpha_{ij}^{13} /\sigma_{\text{m}}^{2} - \frac{1}{4}\gamma_{ij}^{13}$
E[*X*_*im*_^2^ *X*_*jm*_^2^]	$\frac{1}{2}\alpha_{ij}^{22} \sigma_{\text{m}}^{2} - \frac{1}{4}\gamma_{ij}^{22} \sigma_{\text{m}}^{4}$	$\frac{1}{2}\alpha_{ij}^{22} /\sigma_{\text{m}}^{2} - \frac{1}{4}\gamma_{ij}^{22}$

#### Second approximation

The expectations ${\text{E}}[ {x_{cm}^{2} x_{rim}^{2} }]$ and ${\text{E}}\left[ {x_{{cm}}^{2} x_{{rim}} x_{{cl}} x_{{ril}} } \right]$are also obtained from the coefficients in Table 1, i.e.: ${\text{E}}\left[ {x_{cm}^{2} x_{rim}^{2} } \right] = \frac{1}{2}\sigma_{m}^{2} \alpha_{ci}^{22} - \frac{1}{4}\sigma_{m}^{4} \gamma_{ci}^{22}$ and, when markers are independent, E[*x*_*cm*_*X*_*rim*_*x*_*cl*_*X*_*ril*_] = E[*x*_*cm*_*X*_*rim*_].E[*x*_*cl*_*X*_*ril*_] = 4*a*_*ci*_^2^*σ*_m_^2^*σ*_l_^2^. Let $\rho_{m} = \frac{{n_{r} \sigma_{m}^{2} }}{{\lambda_{\beta } + n_{r} \sigma_{m}^{2} }}$. After some algebra, it appears that:

3 $$\begin{aligned}   \tilde{\tilde{E}}\left[ {r_{{q_{{\text{c}}} ,\hat{q}_{{\text{c}}} }}^{2} } \right] =   &1 - \frac{{\lambda _{\beta } }}{{n_{r} \tau }}\left\{ {\left( {\sum\limits_{m} {\rho _{m} } } \right) + \left( {\sum\limits_{m} {\rho _{m}^{2} } } \right)\left( {1 + 4\bar{a}_{{ci}}^{2}  + \frac{1}{4}\bar{\gamma }_{{ci}}^{{22}} } \right)} \right. \\ &   \left. {\quad  - \left( {\sum\limits_{m} {\rho _{m} } } \right)^{2} \left( {4\bar{a}_{{ci}}^{2} } \right) - \left( {\sum\limits_{m} {\frac{{\rho _{m}^{2} }}{{\sigma _{m}^{2} }}} } \right)\left( {\frac{1}{2}{\bar{\alpha}}_{{ci}}^{{22}} } \right)} \right\} \\  \end{aligned}  $$

where $\bar{a}_{ci}^{2}$, $\bar{\alpha}_{ci}^{22}$ and $\bar{\gamma }_{ci}^{22}$ are the means of the corresponding coefficients, considering all possible *i* reference individuals.

#### Parameter estimation

The parameters *τ* = ∑_*m*_*σ*_*m*_^2^ and *τ*_2_ = ∑_*m*_*σ*_*m*_^4^ that appear in the first approximation, and the parameters $\sum\nolimits_{m} {\rho_{m} }$, ∑_*m*_*ρ*_*m*_^2^ and $\sum\nolimits_{m} {\frac{{\rho_{m}^{2} }}{{\sigma_{m}^{2} }}}$ that appear in the second approximation, are unknown. Their expectations can be derived by making assumptions about the distribution of the marker allele frequencies. They were derived assuming either a uniform distribution of allele frequencies or the U-shaped distribution of allelic frequencies proposed by Goddard [16]: $f( p) = {k \mathord{\left/ {\vphantom {k {2{\text{p}}\left( {1 - {\text{p}}} \right) }}} \right. \kern-0pt} {2{\text{p}}\left( {1 - {\text{p}}} \right) }}$ with the constant *k* estimated as 1/log2*N*_*e*_, *N*_*e*_ being the effective size of the reference population. The expectations of the parameters are in Table 3. The corresponding algebra is detailed in Additional file 2.

**Table 3:** Expectation of elements involved in precision formulae when a uniform $( f ( p ) = 1 )$ or a U shaped distribution of allelic frequencies is assumed $\left( {f( p) = {k \mathord{\left/ {\vphantom {k {2p\left( {1\text{ - }p} \right)}}} \right. \kern-0pt} {2p\left( {1-p} \right)}}} \right)$

Element	Expectation	
	Uniform	U shaped
E[*σ*_*m*_^2^]	1/3	*k*
E[*σ*_*m*_^4^]	2/15	*k*/3
E[*ρ*_*m*_]	$1 - 2\frac{h}{\omega }\theta$	$\frac{k}{\omega }\theta$
E[*ρ*_*m*_^2^]	$\left( {\frac{4\theta }{\omega } + \frac{2}{h}} \right)\left( {\frac{1 + h}{1 + 4h}} \right)^{2} - \frac{4\theta h}{\omega }$	$\frac{k}{{\omega^{2} }}\left[ {\theta \left( {\omega - \frac{2h}{\omega }} \right) - 1} \right]$
E[*ρ*_*m*_^2^/*σ*_*m*_^2^]	$\frac{1}{{\omega^{2} }}\left[ {\theta \left( {\omega - \frac{2h}{\omega }} \right) - 1} \right]$	$\frac{k}{{2\omega^{3} }}\left\{ {2\theta + \frac{\omega }{h}} \right\}$

A large effective size $N_{e}$ of the population was assumed to make 1/*N*_*e*_ negligible $\theta = \log \left( {\left| {\frac{1 + \omega }{1 - \omega }} \right|} \right),\omega = \sqrt {1 + 4h}$ $h = {{{{\uplambda }}_{{{\upbeta }}} } \mathord{\left/ {\vphantom {{{{\uplambda }}_{{{\upbeta }}} } {2n_{r} }}} \right. \kern-0pt} {2n_{r} }}, {{\uplambda }}_{{{\upbeta }}} = {{\sigma_{e}^{2} } \mathord{\left/ {\vphantom {{\sigma_{e}^{2} } {\sigma_{{{\upbeta }}}^{2} }}} \right. \kern-0pt} {\sigma_{{{\upbeta }}}^{2} }}$

The parameters *τ* and *τ*_2_ are linked to the number *M*_*e*_ of independent segments. This quantity *M*_*e*_ was defined by Goddard [16] as the number of independent chromosomal segments which would give the same variance of genomic covariances $c_{ij}$ between individuals *i* and *j* as that observed, *i.e.* when LD exists. Conditional on the genotypic observation, the genomic covariance between two individuals is cov(*q*_*i*_,*q*_*j*_|**X**) = *σ*_β_^2^∑_q_X_iq_X_jq_ = *c*_*ij*_. Thus, v_X_(*c*_*ij*_) = *σ*_β_^4^v[∑_q_X_iq_X_jq_], or v_X_(*c*_*ij*_) = *σ*_β_^4^(∑_q_v(X_iq_X_jq_) + ∑_q_∑_q′≠q_cov(X_iq_X_jq_,X_iq′_X_jq′_)). When the markers are in linkage equilibrium, the covariance term is null, and ${\text{v}}_{\text{X}} \left( {c_{ij} } \right) = \sigma_{{{\upbeta }}}^{4} \left[ {\frac{1}{2}\tau \alpha_{ij}^{22} - \frac{1}{4}\tau_{2} \gamma_{ij}^{22} - \frac{1}{4}{\text{a}}_{\text{ij}}^{2} \tau_{2} } \right]$. If individuals are unrelated, *α*_*ij*_^22^ = 0, *γ*_*ic*_^22^ = −4 and a_ij_ = 0. Thus, v_X_(*c*_*ij*_) = *σ*_β_^4^*τ*_2_. As *σ*_*q*_^2^ = *σ*_β_^2^*τ*, ${\text{v}}_{\text{X}} \left( {c_{ij} } \right) = \sigma_{q}^{4} \frac{{\tau_{2} }}{{\tau^{2} }}$. From the appendix in the paper of Goddard [16], this variance is v_X_(*c*_*ij*_) = *σ*_*q*_^4^/*M*_*e*_. Thus:

4 $$ M_{e} = \tau^{2} /\tau_{2} . $$

It must be emphasized that *M*_*e*_, which depends on the variability of allele frequencies, is not the number of markers *n*_*M*_.

#### The case of unrelated individuals

The first approximation gives results similar to Goddard et al. [18] when individuals are not related. In this case, **A** = **I** then ${\mathbf{T}} = \frac{1}{1 + \gamma }{\mathbf{I}} = \frac{{\sigma_{e}^{2} }}{{\sigma_{q}^{2} + \sigma_{e}^{2} }}{\mathbf{I}} = \frac{{\sigma_{e}^{2} }}{{\sigma^{2} }}{\mathbf{I}}$. The ratio $\frac{\gamma }{1 + \gamma } = \frac{{\sigma_{q}^{2} }}{{\sigma^{2} }} = \nu^{2}$ is the proportion of the phenotypic variance explained by the molecular score.

$$ \begin{aligned} {\text{E}}\left[ {{\varvec{\Lambda}}_{\text{cc}} } \right] = \frac{1}{{\sigma_{e}^{2} }}\left\{ {{\mathbf{T}}_{\text{cc}} - \gamma^{2} \left\{ {{\mathbf{TATAT}}} \right\}_{\text{cc}} + \frac{{\gamma^{2} }}{{\tau^{2} }}\left\{ {{\mathbf{T}}{\text{E}}\left[ {{\mathbf{G}}^{ *} {\mathbf{TG}}^{ *} } \right]{\mathbf{T}}} \right\}_{\text{cc}} } \right\} \hfill \\             = \frac{1}{{\sigma_{e}^{2} }}\left\{ {\frac{1}{1 + \gamma } - \frac{{\gamma^{2} }}{{\left( {1 + \gamma } \right)^{3} }} + \frac{{\gamma^{2} }}{{\tau^{2} \left( {1 + \gamma } \right)^{2} }}{\text{E}}\left[ {{\mathbf{G}}^{ *} {\mathbf{TG}}^{ *} } \right]_{\text{cc}} } \right\} \hfill \\ \end{aligned} $$

$$ \left\{ {{\text{E}}\left[ {{\mathbf{G}}^{ *} {\mathbf{TG}}^{ *} } \right]} \right\}_{\text{cc}} = \sum\nolimits_{l} {\sum\nolimits_{k} {t_{kl} \left( {\frac{1}{2}\tau \alpha_{cckl}^{1111} - \frac{1}{4}\tau_{2} \gamma_{cckl}^{1111} + 4a_{ck} a_{cl} \left[ {\tau^{2} - \tau_{2} } \right]} \right)} } $$

**T** being diagonal, this equation simplifies to $$ \left\{ {{\text{E}}\left[ {{\mathbf{G}}^{ *} {\mathbf{TG}}^{ *} } \right]} \right\}_{\text{cc}} = \sum\nolimits_{k} {t_{kk} \left( {\frac{1}{2}\tau \alpha_{ck}^{22} - \frac{1}{4}\tau_{2} \gamma_{ck}^{22} + 4a_{ck}^{2} \left[ {\tau^{2} - \tau_{2} } \right]} \right)}, $$ with $$ t_{kk} = \frac{1}{1 + \gamma } $$, $$ \alpha_{ck}^{22} = 0 \;{\text{and}}\; \gamma_{ck}^{22} = - 4\;{\text{if}} \;c \ne k $$, $$ \alpha_{cc}^{22} = \alpha_{c}^{4} = 2 \;{\text{and}} \;\gamma_{c}^{4} = 0 $$, $$ {\text{a}}_{\text{cc}} = {\text{a}}_{\text{kk}} = \frac{1}{2} $$ and a_ck_ = 0. Hence $$ \left\{ {{\text{E}}\left[ {{\mathbf{G}}^{ *} {\mathbf{TG}}^{ *} } \right]} \right\}_{\text{cc}} = \frac{1}{1 + \gamma }\left\{ {\tau + \tau^{2} - \tau_{2} + n_{r} \tau_{2} } \right\} $$, and $$ {\text{E}}\left[ {{\varvec{\Lambda}}_{\text{cc}} } \right] = \frac{1}{{\sigma_{e}^{2} }}\left\{ {\frac{1}{1 + \gamma } + \frac{{\gamma^{2} }}{{\left( {1 + \gamma } \right)^{3} }}\left( {\frac{1}{\tau } + \left( {n_{r} - 1} \right)\frac{{\tau_{2} }}{{\tau^{2} }}} \right)} \right\} $$. $$ \begin{aligned} {\text{E}}\left[ {v\left( {\hat{q}_{c} } \right)} \right] 
&= {\text{E}}\left[ {{\mathbf{V}}_{\text{cc}}^{ *} } \right] - \frac{1}{{{\text{E}}\left[ {{\varvec{\Lambda}}_{\text{cc}} } \right]}}\\  &= \sigma^{2} - \sigma^{2} \frac{1}{{1 + \nu^{4} \left( {\frac{1}{\tau } + \frac{{n_{r} - 1}}{{M_{e} }}} \right)}} \\  & = \sigma^{2} \frac{{\nu^{4} \left( {\frac{1}{\tau } + \frac{{n_{r} - 1}}{{M_{e} }}} \right)}}{{1 + \nu^{4} \left( {\frac{1}{\tau } + \frac{{n_{r} - 1}}{{M_{e} }}} \right)}}. \\ \end{aligned} $$ If we neglect $$ \frac{1}{\tau } - \frac{1}{{M_{e} }} $$ and use $$ \nu^{2} = \frac{{\sigma_{q}^{2} }}{{\sigma^{2} }} $$, we get $$ {\text{E}}\left[ {v\left( {\hat{q}_{c} } \right)} \right] = \sigma_{q}^{2} \frac{{\nu^{2} \frac{{n_{r} }}{{M_{e} }}}}{{1 + \nu^{4} \frac{{n_{r} }}{{M_{e} }}}} $$, which is similar but not identical to the equation in Goddard et al. [18] ($$ \sigma_{q}^{2} \frac{{\nu^{2} \frac{{n_{r} }}{{M_{e} }}}}{{1 + \nu^{2} \frac{{n_{r} }}{{M_{e} }}}} $$). Finally, the precision is estimated as:

5 $$ {\tilde{\text{E}}}\left[ {r_{{q_{\text{c}} ,\hat{q}_{c} }}^{2} } \right] = \frac{{\nu^{2} \frac{{n_{r} }}{{M_{e} }}}}{{1 + \nu^{4} \frac{{n_{r} }}{{M_{e} }}}} .$$

In this situation of unrelatedness between the candidate and the reference population, the second approximation simplifies to ${{\tilde{\tilde{E}}}}\left[ {r_{{q_{\text{c}} ,\, \hat{q}_{c} }}^{2} } \right] = 1 - \lambda_{\beta } \frac{{E\left[ {\mathop \sum \nolimits_{m} \rho_{m} } \right]}}{{n_{r} \tau }}$. From Table 3, we have $E\left[ {\sum\nolimits_{m} {\rho_{m} } } \right] = n_{M} \frac{k}{\omega }\theta$ with $\theta = \log \left( {\left| {\frac{1 + \omega }{1 - \omega }} \right|} \right),\omega = \sqrt {1 + 4h}$, $h = {{{{\uplambda }}_{{{\upbeta }}} } \mathord{\left/ {\vphantom {{{{\uplambda }}_{{{\upbeta }}} } {2n_{r} }}} \right. \kern-0pt} {2n_{r} }}$ and *k* = 1/ log 2*N*_*e*_. As *λ*_*β*_ = *τ*/*γ*, we found:

6 $$ {{\tilde{\tilde{E}}}}\left[ {r_{{q_{\text{c}} ,\hat{q}_{c} }}^{2} } \right] = 1 - \frac{{n_{M} {\text{k}}\theta  }}{{\gamma n_{r} \omega }} . $$

#### Non-independence between reference and candidate population, a simple example

We consider the situation of a candidate that is the son of one of the *n*_*r*_ individuals in P_r_ (say the first in the list) while still assuming that reference individuals are unrelated. In this situation, the pedigree relationship matrix is $$ \left( {\begin{array}{*{20}c} {\begin{array}{*{20}c} 1 & {0.5 } \\ {0.5} & 1 \\ \end{array} } & {\bf 0} \\ {\bf 0} & {{\mathbf{I}}_{{{\text{n}}_{\text{r}} - 1}} } \\ \end{array} } \right) $$, which results in a **T** matrix $$ \left( {\begin{array}{*{20}c} {\begin{array}{*{20}c} a & b \\ b & a \\ \end{array} } & {\bf 0} \\ {\bf 0} & {\frac{1}{1 + \gamma }{\mathbf{I}}_{{{\text{n}}_{\text{r}} - 1}} } \\ \end{array} } \right) $$ with $\gamma = \frac{{\sigma_{q}^{2} }}{{\sigma_{e}^{2} }}, a = \frac{1 + \gamma }{{\left( {1 + \gamma } \right)^{2} - {1 \mathord{\left/ {\vphantom {1 {4\gamma^{2} }}} \right. \kern-0pt} {4\gamma^{2} }}}}$ and $b = - \frac{{{\gamma \mathord{\left/ {\vphantom {\gamma 2}} \right. \kern-0pt} 2}}}{{\left( {1 + \gamma } \right)^{2} - {1 \mathord{\left/ {\vphantom {1 {4\gamma }}} \right. \kern-0pt} {4\gamma }}}}$. Applications of formulae (2) and (3) are described in Additional file 3. The expected approximate precision with the first approach is:

7 $$ {\tilde{\text{E}}}\left[ {r_{{q_{\text{c}} ,\hat{q}_{\text{c}} }}^{2} } \right] \sim \frac{1}{{\nu^{2} }} - \frac{1}{{\gamma {\text{a}} + \gamma^{3} \frac{{{{\tau }} - {{\tau }}_{2} }}{{{{\tau }}^{2} }}{\text{c}}1 + \gamma^{3} \frac{{{{\tau }}_{2} }}{{{{\tau }}^{2} }}\left[ {{\text{c}}2 + \frac{{{\text{n}}_{\text{r}} - 1}}{1 + \gamma }{\text{c}}3} \right]}} , $$

where $c1 = \left( {{\text{a}} + {\text{b}}} \right)^{3} + \left( {{\text{a}}^{2} + {\text{b}}^{2} } \right)\frac{1}{2}{\text{a}}$, $c2 = \frac{1}{4}{\text{a}}\left( {{\text{b}}^{2} - {\text{a}}^{2} } \right)$ and $c3 = {\text{a}}^{2} + {\text{b}}^{2} + \frac{1}{2}   {\text{ab}}$. And with the second approach:

8 $$ {{\tilde{\tilde{E}}}}\left[ {r_{{q_{\text{c}} ,\hat{q}_{c} }}^{2} } \right] = 1 - \frac{{{\text{n}}_{\text{M}} k\theta }}{{\gamma {\text{n}}_{\text{r}} \omega }} - \frac{{{\text{n}}_{\text{M}} k}}{{4\gamma n_{r}^{2} \omega^{2} }}\left( {5\theta \omega - \frac{{\left( {10h + 2} \right)\theta }}{\omega } - 5 - {\text{n}}_{\text{M}} {\text{k}}\theta^{2} - \frac{1}{h}} \right) . $$

#### Alternative genotypes codification

In all the previous developments, genotypes were coded *x*_*tim*_ = *a*_*tim*_ − 2*p*_*tm*_ and *w*_*tiq*_ = *a*_*tiq*_ − 2*p*_*tq*_. Alternatively, we could define *x*_*tim*_ = (*a*_*tim*_ − 2*p*_*tm*_)/*σ*_*tm*_ and *w*_*tiq*_ = (*a*_*tiq*_ − 2*p*_*tq*_)/*σ*_*tq*_. The relation between genetic and marker variances becomes *σ*_*q*_^2^ = *n*_*M*_*σ*_β_^2^ and the relation between pedigree and genomic matrices becomes E[G*] = **A***n*_*M*_. Thus, formulae (1) and (2) are still valid when replacing *τ* by *n*_*M*_. The ${\text{E}}\left[ {X_{im}^{{d_{i} }} X_{jm}^{{d_{j} }} \cdots X_{Km}^{{d_{K} }} } \right]$ elements derived in Additional file 1, need to be divided by $\sigma_{m}^{{d_{i} + d_{j} + \cdots + d_{K} }}$. Table 2 gives the expectations with this alternative codification of genotypes. The quantity {E[**XX**^′^**TXX**^′^]}_ij_ has to be changed, using ${{\zeta }} = \frac{1}{{n_{M} }}\sum\nolimits_{m} {\frac{1}{{\sigma_{m}^{2} }}}$. We have: $\sum\nolimits_{\text{m}} {{\text{E}}\left[ {X_{im} X_{km} X_{jm} X_{lm} } \right] = \frac{{n_{M} }}{2}{{\zeta \alpha }}_{\text{ijkl}}^{1111} - \frac{{n_{M} }}{4}{{\gamma }}_{\text{ijkl}}^{1111} }$, ∑_m_E[*X*_*im*_*X*_*km*_] = 2*n*_*M*_*a*_*ik*_, and ∑_m_(E[*X*_*im*_*X*_*km*_]E[*X*_*jm*_*X*_*lm*_]) = 4*n*_*M*_*a*_*ik*_*a*_*jl*_. Thus:

$$ \begin{aligned}& \left\{ {{\text{E}}\left[ {{\mathbf{XX^{\prime}TXX^{\prime}}}} \right]} \right\}_{\text{ij}}  \\&\quad= \sum\nolimits_{l} {\sum\nolimits_{k} {t_{kl} \left( {\frac{{n_{M} }}{2}{{\zeta }}\alpha_{ijkl}^{1111} - \frac{{n_{M} }}{4}\gamma_{ijkl}^{1111} + 4n_{M} \left( {n_{M} - 1} \right)a_{ik} a_{jl} } \right)} }.  \\ \end{aligned} $$

When applied to the case of unrelated individuals and no LD, *i.e.* when $t_{kk} = \frac{1}{1 + \gamma }$, $\alpha_{ck}^{22} = 0 \;{\text{and}}\; \gamma_{ck}^{22} = - 4 \;{\text{if}}\; c \ne k$, $\alpha_{cc}^{22} = \alpha_{c}^{4} = 2 \;{\text{and}}\; \gamma_{c}^{4} = 0$, ${\text{a}}_{\text{cc}} = {\text{a}}_{\text{kk}} = \frac{1}{2}$ and a_ck_ = 0, we have:

$$ \begin{aligned} {\text{E}}\left[ {{\varvec{\Lambda}}_{\text{cc}} } \right] &= \frac{1}{{\sigma_{e}^{2} }}\left\{ {\frac{1}{1 + \gamma } - \frac{{\gamma^{2} }}{{\left( {1 + \gamma } \right)^{3} }} + \frac{{\gamma^{2} }}{{n_{M}^{2} \left( {1 + \gamma } \right)^{2} }}} \right. . \hfill \\&\quad \left. {\sum\nolimits_{k} {\frac{1}{1 + \gamma }\left( {\frac{{n_{M} }}{2}{{\zeta }}\alpha_{cckk}^{1111} - \frac{{n_{M} }}{4}\gamma_{cckk}^{1111} + 4n_{M} \left( {n_{M} - 1} \right)a_{ck} a_{ck} } \right)} } \right\}, \hfill \\ \end{aligned} $$

which gives:

$$ {\text{E}}\left[ {{\varvec{\Lambda}}_{\text{cc}} } \right] = \frac{1}{{\sigma_{e}^{2} \left( {1 + \gamma } \right)}}\left\{ {1 - \frac{{\gamma^{2} }}{{\left( {1 + \gamma } \right)^{2} }}\left( {1 - \frac{{{{\zeta }} + n_{R} + n_{M} - 1}}{{n_{M} }}} \right)} \right\} $$

*i.e.*$$ {\text{E}}\left[ {{\varvec{\Lambda}}_{\text{cc}} } \right] = \frac{1}{{\sigma^{2} }}\left\{ {1 + \nu^{4} \left( {\frac{{{{\zeta }} + n_{R} - 1}}{{n_{M} }}} \right)} \right\} $$ and $$ {\tilde{\text{E}}}\left[ {r_{{q_{\text{c}} ,\hat{q}_{\text{c}} }}^{2} } \right] = \frac{{\nu^{2} \frac{{{{\zeta }} + n_{R} - 1}}{{n_{M} }}}}{{1 + \nu^{4} \frac{{{{\zeta }} + n_{R} - 1}}{{n_{M} }}}} $$.

Based on Additional file 2, the expectation of ζ parameter is $\frac{k}{4}\left[ {2\log \left( {N_{e} - 1} \right) + 2\frac{{N_{e} \left( {N_{e} - 2} \right)}}{{N_{e} - 1}}} \right]$ for a U-shaped distribution of alleles frequencies and log (*N*_*e*_ − 1) for a uniform distribution.

### The case of markers in linkage disequilibrium

So far, following Goddard [16], we considered the situation of *n*_*M*_ independent segments that each carries a single QTL in LD with a single marker. More typically, the genomic information consists of a large number of non-independent markers. This non-independence comes from long-term effects due to bottlenecks, mutations, migrations, etc. and short-term effects due to family structure.

#### Effective and equivalent numbers of independent loci

We based our developments on the very fruitful concept of the effective number of loci that Goddard defined as “the number of independent loci that gives the same variance of realized relationships as that obtained in the more realistic situation” (Goddard [16] appendix). Since our objective was to predict the reliability of GEBV, we now suggest the alternative definition of an “equivalent number of independent loci” which would give the reliability of GEBV for unrelated individuals when considering a sub-set of independent markers that would be identical to the reliability obtained when considering the full set of markers. From the derivation of the reliability given previously, defining **x**_**c**_^**i**^ and ${\mathbf{X}}_{{\mathbf{r}}}^{{\mathbf{i}}}$ as the genotype meed ${\text{E}}_{{{\mathbf{x}}_{{\mathbf{c}}} ,{\mathbf{X}}_{{\mathbf{r}}} }} \left[ {v\left( {\hat{q}_{c} |{\mathbf{x}}_{{\mathbf{c}}} ,{\mathbf{X}}_{{\mathbf{r}}} } \right)} \right] = {\text{E}}_{{{\mathbf{x}}_{\text{c}}^{\text{i}} ,{\mathbf{X}}_{\text{r}}^{\text{i}} }} \left[ {v\left( {\hat{q}_{c} |{\mathbf{x}}_{{\mathbf{c}}}^{{\mathbf{i}}} ,{\mathbf{X}}_{{\mathbf{r}}}^{{\mathbf{i}}} } \right)} \right].$ With a few simplifying assumptions (identical distribution of genotypes in the reference and candidate populations and equal genotypic variance at all loci) a simple formula can be derived [see Additional file 4]:

9 $$ n_{{M_{i} }} = n_{M} \frac{1 + \gamma }{\gamma }\left( {1 - tr\left[ {\left( {{\text{E}}\left[ {{\mathbf{X}}^{\prime}_{{\mathbf{r}}} {\mathbf{X}}_{{\mathbf{r}}} } \right] + {{\uplambda }}_{{{\upbeta }}} {\mathbf{I}}} \right)^{ - 1} } \right]\frac{{\mathop \sum \nolimits_{m} \sigma_{m}^{2} /n_{M} }}{\gamma }} \right) , $$

where *tr*[M] is the trace of matrix M.

Once marker allele frequencies and between-marker LD are estimated in a population of interest, the equivalent number of independent loci which can be estimated from formula (9) and this parameter can be used in models that predict the genetic gain expected from a genomic selection scheme applied to this population.

In the more general situation, prior to the observation of the **X**_**r**_ matrix, a simple approximation for $n_{{M_{i} }}$ is obtained assuming equal variances *σ*_*m*_^2^ = *s*^2^, and using the relation between expected LD and effective population size *N*_*e*_ as derived by Sved [32]: ${\text{E}}\left[ {2\Updelta_{ml} } \right] = {{\sigma_{m} \sigma_{l} } \mathord{\left/ {\vphantom {{\sigma_{m} \sigma_{l} } {\sqrt {1 + 4N_{e} d_{lm} } }}} \right. \kern-0pt} {\sqrt {1 + 4N_{e} d_{lm} } }}$ with *d*_*lm*_ the distance between ordered loci *l* and *m*, such that $d_{lm} = {{\left| {l - m} \right|L} \mathord{\left/ {\vphantom {{\left| {l - m} \right|L} {n_{M} }}} \right. \kern-0pt} {n_{M} }}$ with *L* the genome length in Morgan. With those hypotheses, let U = *tr*[(*γn*_*R*_**R** + *n*_*M*_**I**)^−1^] with ${\mathbf{R}}_{\text{ml}} = \sqrt {{{n_{M} } \mathord{\left/ {\vphantom {{n_{M} } {\left( {n_{M} + 4N_{e} \left| {l - m} \right|L} \right)}}} \right. \kern-0pt} {\left( {n_{M} + 4N_{e} \left| {l - m} \right|L} \right)}}}$.

In this simplified situation, the equivalent number of loci is [See Additional file 4]:

10 $$ n_{{M_{i} }} = n_{M} \frac{{n_{R} \gamma \left( {1 - U} \right)}}{{n_{R} \gamma - n_{M} \left( {1 - U} \right)}} . $$

#### Towards an exact treatment of linkage disequilibrium

For a complete treatment of the LD situation, it is necessary to estimate the expectations of the product of four genetic values. For instance, with the second approximation [formula (2)], we need to compute E[*x*_*cm*_*X*_*rim*_*x*_*cl*_*X*_*ril*_]. Let $X_{im} = g_{imf} + g_{imd}$, where *g*_*imf*_ and *g*_*imd*_ are the “values” of the alleles transmitted to individual *i* by its father and its dam, with *g*_*imf*_ and $g_{imd} = \left( {0 \;or\; 1} \right) - p_{m}$. They will be called allelic values in the following. Equivalent terms are defined for *x*_*cl*_,$x_{cm}$ and *X*_*il*_. The random variable *M*_*cls*_ is the allele of individual *c* received from *s* at locus $l \left(f\, {\rm or}\, d \right)$. $M_{cmt} , M_{ilu}\,  {\text{and}}\, M_{imv}$ are defined similarly.

11 $$ \begin{aligned} & {\text{E}}\left[ {x_{cl} x_{cm} X_{il} X_{im} } \right]  \\ &\quad= \sum\limits_{{s \in \left\{ {f,d} \right\}}} {\sum\limits_{{t \in \left\{ {f,d} \right\}}} {\sum\limits_{{u \in \left\{ {f,d} \right\}}} {\sum\limits_{{v \in \left\{ {f,d} \right\}}}  {{\text{E}}\left[ {g_{cls} g_{cmt} g_{ilu} g_{imv} } \right]} } } } \hfill \\ \end{aligned}. $$

For the candidate *c* as for the reference individual *i*, the pair of genetic values may originate from the same parent (and coded on the same chromosome) or not, giving four types of $\left( {g_{cls} ,g_{cmt} ,g_{ilu,} g_{imv} } \right)$ vectors. In type 1 ($s = t \, and \, u = v$), both alleles (belonging to loci $m\; {\text{and}} \;l$) of each pair of loci (one for *c* and one for *i*) are on the same chromosome (may be from the two fathers, the two dams, *c*’s father and *i*’s dam or *i*’s father and* c*’s dam). In type 2 ($s = t \, and \, u \ne v$), both alleles (belonging to loci $m \;{\text{and}} \;l$) of the candidate are on the same chromosome, while alleles of the reference individual *i* are not on the same chromosome.Type 3 ($s \ne t \, and \, u = v$) is the reverse from type 2. In type 4 ($s \ne t \, and \, u \ne v$), alleles of loci $m\; {\text{and}} \;l$ of both individuals and *i* are on different chromosomes.

For each of these situations, the identity by descent (IBD) status between alleles at locus $m$ on chromosomes *ct* and *iv*, and at locus $l$ on chromosomes *cs* and *iu* are considered. There are four, as follows:



Thus, 16 terms involved in E[*x*_*cl*_*x*_*cm*_*X*_*il*_*X*_*im*_] are given by:12

As described in Additional file 5, only seven ${\text{E}}\left[ {g_{cls} g_{cmt} g_{ilu} g_{imv} |{\mathcal{S}}_{k} } \right]$ are non-null (Table 4). Principles on which the probabilities *φ*_*k*_^*stuv*^ are estimated and basic examples are described in Additional file 5.

**Table 4:** Expectations of products of four allelic values received by two individuals at two loci depending on the IBD status and parental origins of the alleles

${\mathcal{S}}$	${\mathcal{T}}$	${\text{E}}\left[ {g_{cls} g_{cmt} g_{ilu} g_{imv} |{\mathcal{S} }\,\& \text{ }\,T} \right]$
${\mathcal{S}}_{ml}$	$s = t \, and \, u = v$	$\left( {1 - p_{m} } \right)\left( {1 - p_{l} } \right)p_{m} p_{l} + \Updelta_{lm} \left( {1 - 2p_{l} } \right)\left( {1 - 2p_{m} } \right)$
	$s = t \, \, and \, \, u \ne v$	$\left( {1 - p_{m} } \right)\left( {1 - p_{l} } \right)p_{m} p_{l} + \Updelta_{lm} \left( {1 - 2p_{l} } \right)\left( {1 - 2p_{m} } \right)$
	$s \ne t \, and \, u = v$	$\left( {1 - p_{m} } \right)\left( {1 - p_{l} } \right)p_{m} p_{l} + \Updelta_{lm} \left( {1 - 2p_{l} } \right)\left( {1 - 2p_{m} } \right)$
	$s \ne t \, and \, u \ne v$	(1 − *p*_*m*_) (1 − *p*_*l*_)*p*_*m*_ *p*_*l*_
	$s = t \, and \, u = v$	$\Updelta_{lm}^{2} \times {{\left[ {p_{m}^{3} + \left( {1 - p_{m} } \right)^{3} } \right]} \mathord{\left/ {\vphantom {{\left[ {p_{m}^{3} + \left( {1 - p_{m} } \right)^{3} } \right]} {\left[ {p_{m} \left( {1 - p_{m} } \right)} \right]}}} \right. \kern-0pt} {\left[ {p_{m} \left( {1 - p_{m} } \right)} \right]}}$
	$s = t \, and \, u = v$	$\Updelta_{lm}^{2} \times {{\left[ {p_{l}^{3} + \left( {1 - p_{l} } \right)^{3} } \right]} \mathord{\left/ {\vphantom {{\left[ {p_{l}^{3} + \left( {1 - p_{l} } \right)^{3} } \right]} {\left[ {p_{l} \left( {1 - p_{l} } \right)} \right]}}} \right. \kern-0pt} {\left[ {p_{l} \left( {1 - p_{l} } \right)} \right]}}$
	$s = t \, and \, u = v$	$\Updelta_{lm}^{2} \times \left( {1 - 2p_{m} } \right)\left( {1 - 2p_{l} } \right)$

Only non-null terms are given

*p*_*m*_ and *p*_*l*_ are the frequencies of the most frequent alleles at loci *m* and *l*. $\Updelta_{lm}$ is the linkage disequilibrium measure between *m* and *l*

$g_{cls} = \left( {0 \,or\, 1} \right) - p_{l}$ is the allelic value the candidate received from its parent $s$ at locus *l* etc

${\mathcal{S}}_{ml}$ means *c* and *i* genes are IBD at *m* and *l*,  only at *m* etc

As an illustration, we consider again the situation of a candidate (*c*), that is the son of one of the *n*_*r*_ individuals in P_r_ and assume that *c*^’*s*^dam is unrelated to the sire. In formula (2), the summation over the reference individuals *i* comprises a single term for the sire of the candidate and *n*_*r*_ − 1 terms for the individual that are unrelated to the *c* members of this reference population.

Based on Additional files 1 and 5, expectations involved in the precision formulae (2) are:

E[*x*_*cm*_^2^*X*_*rim*_^2^] = *p*_*m*_(1 − *p*_*m*_), and

$$ \begin{aligned} {\text{E}}\left[ {x_{cl} x_{cm} X_{il} X_{im} } \right] \hfill \\ = \left( {1 - p_{m} } \right)\left( {1 - p_{l} } \right)p_{m} p_{l} + \Updelta_{lm} \left( {1 - 2p_{l} } \right)\left( {1 - 2p_{m} } \right) \hfill \\ + 2\Updelta_{lm}^{2} \left[ {r_{ml} \left( {\frac{{p_{m}^{3} + \left( {1 - p_{m} } \right)^{3} }}{{p_{m} \left( {1 - p_{m} } \right)}} + \frac{{p_{l}^{3} + \left( {1 - p_{l} } \right)^{3} }}{{p_{l} \left( {1 - p_{l} } \right)}}} \right) + \left( {1 - r_{ml} } \right)\left( {1 - 2p_{m} } \right)\left( {1 - 2p_{l} } \right)} \right], \hfill \\ \end{aligned} $$ when *i* is the sire of *c*; and E[*x*_*cm*_^2^*X*_*rim*_^2^] = 4[*p*_*m*_(1 − *p*_*m*_)]^2^ and ${\text{E}}\left[ {x_{cl} x_{cm} X_{il} X_{im} } \right] = 4\Updelta_{lm}^{2} \left( {1 - 2p_{m} } \right)\left( {1 - 2p_{l} } \right)$, when *i* and *c* are unrelated.

### Numerical evaluation

#### Simulation of allele frequencies

In the following numerical evaluation of the formulae derived above, allele frequencies were simulated following an inverse transform sampling (e.g. [32]): $n_{M}$ allele frequency cumulative distribution function values *u*_*m*_ were simulated in a uniform ${\mathcal{U}}\left( {0,1} \right)$, and corresponding allele frequencies *p*_*m*_, *i.e.* such as $u_{m} = \int_{{1/2n_{r} }}^{{p_{m} }} {f\left( p \right)dp}$, computed by $p_{m} = \frac{{\left( {2n_{r} - 1} \right)^{{\left( {2u_{m} - 1} \right)}} }}{{1 + \left( {2n_{r} - 1} \right)^{{\left( {2u_{m} - 1} \right)}} }}$.

#### Basic situation: no LD and unrelated individuals

Convergence of Taylor series and quality of expectation of the reliability approximations were tested for different population sizes ($n_{r} = 500, 1000, 1500 \;{\text{and}}\; 2500$), numbers of markers ($n_{M} = 50, 100, 250, 1000, 1500, 2000 \;{\text{and}}\; 2500$) and proportions of the phenotypic variance explained by the molecular score ($\nu^{2} = 0.1, 0.4 \;{\text{and}} \;0.7$). Given the set of allele frequencies $p_{m} \left( {m = 1 \ldots n_{M} } \right)$, genotypes **X** of *n*_*r*_ + 1 individuals were generated and the **G** matrix was built. The reliability of the candidate individual GEBV, $r^{2} = \frac{{{\text{v}}(\hat{q}_{c} |{\mathbf{X}})}}{{{\text{v}}\left( {q_{\text{c}} |{\mathbf{X}}} \right)}}$ was computed as described in the section «Situation analyzed» , as well as approximations considering 1–10 elements in the Taylor series **I** − **D***γ* + **D**^2^*γ*^2^ − **D**^3^*γ*^3^··· The convergence of the series as predicted by the value (lower or higher than 1) of the matrix’s largest eigenvalue was checked numerically, by estimating the mean of this largest eigenvalue from five simulations in each case studied ($n_{r} = 200\; {\text{to}} \;1000 ;n_{M} = 100 \;{\text{to}}\; 2000 \;{\text{and}} \;\nu^{2} = 0.1, 0.4, 0.7).$

 This limited number of replications was chosen after observation of a very limited variance of this eigenvalue. Finally, the asymptotic values of the suggested approximations [formulae (5) and (6)] were computed using the number of independent segments as described by [4]. The process was repeated 50 times and the means of those exact or approximated reliabilities computed.

**Fig. 1:**
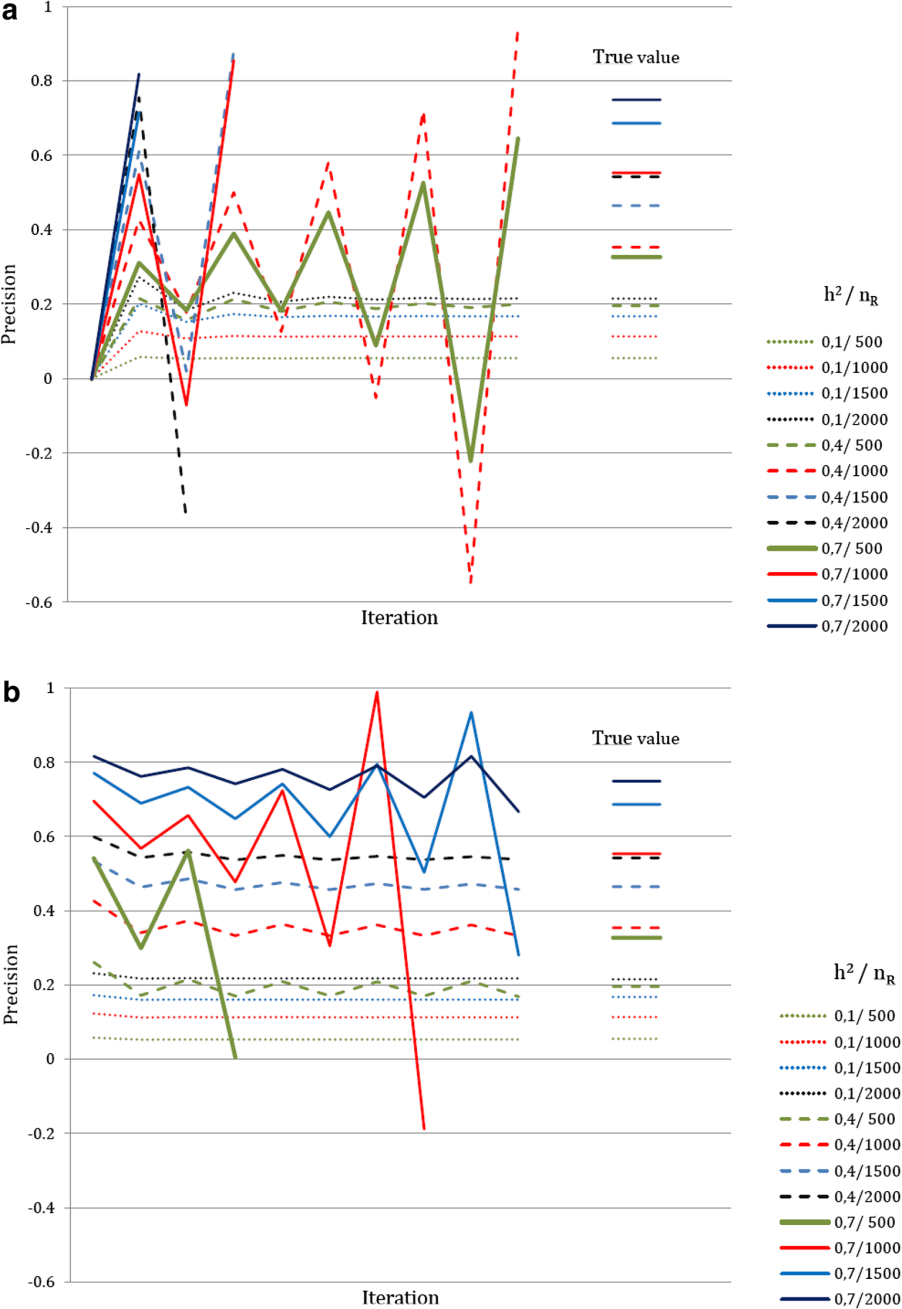
Convergence of the Taylor series as a function of heritability and reference population size ($n_{M} = 2000$). **a** First approximation. **b** Second approximation

**Fig. 2:**
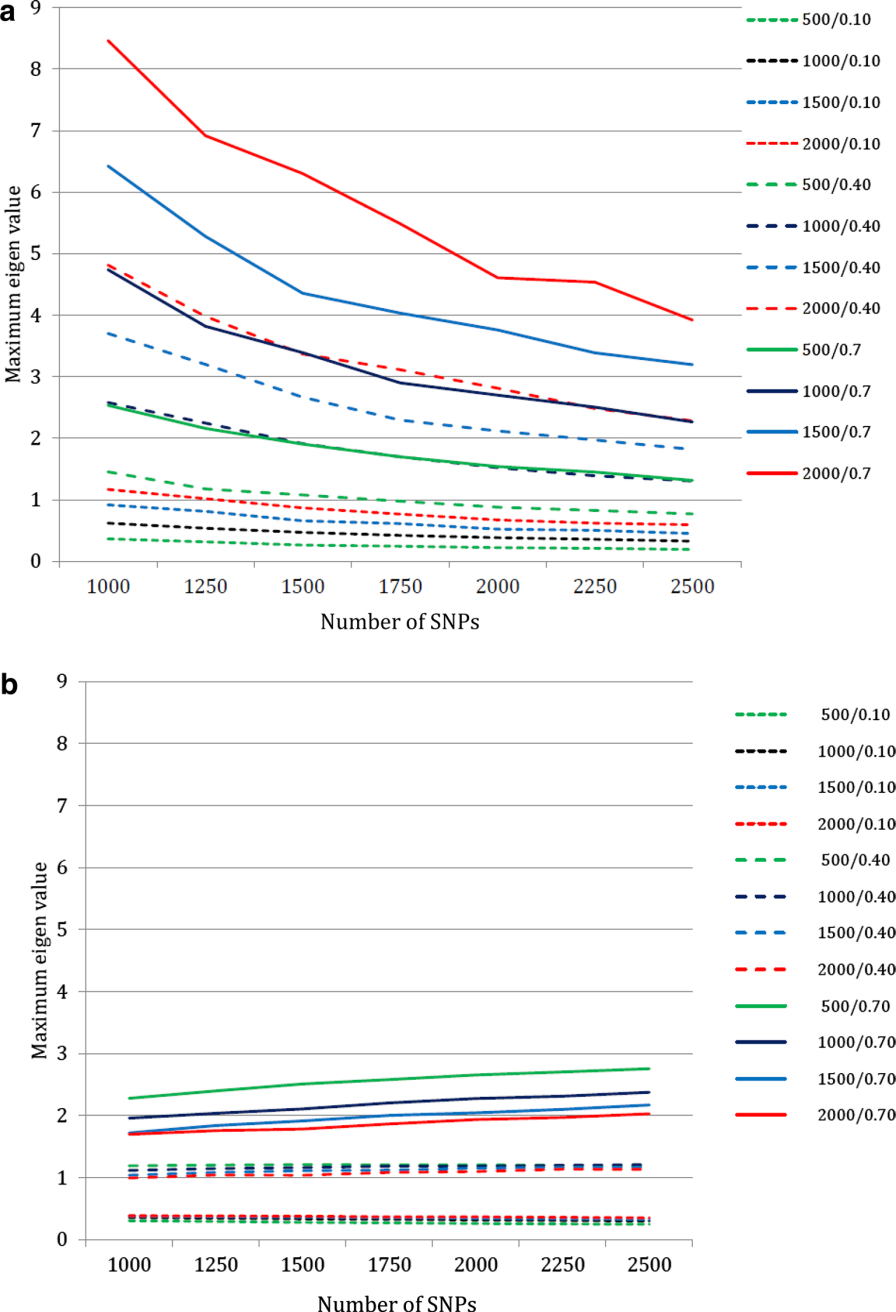
Largest Eigen value of the noise matrix ${\mathbf{D}}\varvec{\gamma}$ involved in the Taylor expansion of the phenotypic variances matrix ${\mathbf{V}}$ as a function of heritability, reference population size and number of markers. **a** First approximation. **b** Second approximation

Figure 1a and b illustrates the convergence of the Taylor series when 2000 markers are used, and Tables 5 and 6 give the results for both approximations when *ν*^2^ = 0.4. The Taylor series converged when the proportion *ν*^2^ of the phenotypic variance explained by the molecular score was low, with oscillations and divergence observed when $\nu^{2} = 0.4$ or 0.7 with the first approximation and *ν*^2^ = 0.7 with the second approximation. These observations were in accordance with the deviation to one of the largest eigenvalue of the matrix involved in the series (Fig. 2a, b). When the series converged, the approximations rapidly reached a plateau, at the 3rd (respectively, 2nd) order for the first (respectively, second) approximation.

**Table 5:** Performances of the first approximation $\left( {\tilde{r}_{{q_{\text{c}} ,\hat{q}_{\text{c}} }}^{2} } \right)$ for an unrelated reference population as a function of the number of markers (*n*_*M*_) and reference population size (*n*_*R*_), assuming *ν* ^2^ = 0.4

*n*_*M*_	*n*_*R*_	True value $\left( {{r}_{{q_{\text{c}} ,\hat{q}_{\text{c}} }}^{2} } \right)$	Approximation $\left( {\hat{r}_{{q_{\text{c}} ,\hat{q}_{\text{c}} }}^{2} } \right)$	Convergence criteria	${\text{E}}\left[ {\hat{r}_{{q_{\text{c}} ,\hat{q}_{\text{c}} }}^{2} } \right]$
50	500	0.92	2.14	2.74	2.05
50	1000	0.96	2.30	2.62	2.23
50	1500	0.96	2.31	2.57	2.28
50	2000	0.97	2.43	2.60	2.37
100	500	0.85	1.71	2.58	1.69
100	1000	0.90	2.01	2.49	2.05
100	1500	0.95	2.21	2.58	2.18
100	2000	0.96	2.31	2.60	2.26
250	500	0.67	1.09	2.53	1.09
250	1000	0.81	1.56	2.53	1.55
250	1500	0.85	1.72	2.54	1.72
250	2000	0.89	1.96	2.53	1.97
1000	500	0.32	0.39	0.61	0.38
1000	1000	0.52	0.72	2.45	0.73
1000	1500	0.64	0.99	2.51	0.99
1000	2000	0.71	1.18	2.51	1.18
1500	500	0.25	0.28	0.27	0.28
1500	1000	0.42	0.54	1.88	0.54
1500	1500	0.54	0.76	2.49	0.76
1500	2000	0.61	0.91	2.50	0.91
2000	500	0.20	0.22	0.20	0.22
2000	1000	0.35	0.43	0.95	0.43
2000	1500	0.46	0.61	2.28	0.61
2000	2000	0.54	0.76	2.48	0.76
2500	500	0.16	0.17	0.16	0.17
2500	1000	0.30	0.35	0.44	0.35
2500	1500	0.40	0.50	1.61	0.50
2500	2000	0.49	0.65	2.40	0.66

The convergence criterion is the value of the Taylor series at order 10

${\text{E}}\left[ {\hat{r}_{{q_{\text{c}} ,\hat{q}_{\text{c}} }}^{2} } \right]$ is the expectation of the first approximation across the distribution of allele frequencies as given in Goddard [16]

**Table 6:** Performances of the second approximation $\left( {\tilde{r}_{{q_{\text{c}} ,\hat{q}_{\text{c}} }}^{2} } \right)$ for an unrelated reference population as a function of the number of markers (*n*_*M*_) and reference population size (*n*_*R*_), assuming *ν* ^2^ = 0.4

*n*_*M*_	*n*_*R*_	True value $\left( {{r}_{{q_{\text{c}} ,\hat{q}_{\text{c}} }}^{2} } \right)$	Approximation $\left( {\tilde{r}_{{q_{\text{c}} ,\hat{q}_{\text{c}} }}^{2} } \right)$	10th order approximation	${\text{E}}\left[ {\tilde{r}_{{q_{\text{c}} ,\hat{q}_{\text{c}} }}^{2} } \right]$
50	500	0.92	0.91	0.91	0.91
50	1000	0.96	0.95	0.94	0.95
50	1500	0.96	0.97	0.97	0.96
50	2000	0.97	0.97	0.97	0.97
100	500	0.85	0.83	0.82	0.83
100	1000	0.90	0.91	0.90	0.91
100	1500	0.95	0.94	0.94	0.94
100	2000	0.96	0.95	0.95	0.95
250	500	0.67	0.71	0.67	0.71
250	1000	0.81	0.83	0.81	0.82
250	1500	0.85	0.88	0.87	0.88
250	2000	0.89	0.90	0.90	0.90
1000	500	0.32	0.41	0.31	0.40
1000	1000	0.52	0.59	0.52	0.57
1000	1500	0.62	0.68	0.64	0.67
1000	2000	0.69	0.73	0.70	0.73
1500	500	0.24	0.32	0.23	0.31
1500	1000	0.42	0.50	0.42	0.48
1500	1500	0.52	0.60	0.53	0.59
1500	2000	0.60	0.66	0.61	0.67
2000	500	0.19	0.26	0.17	0.28
2000	1000	0.34	0.43	0.33	0.44
2000	1500	0.46	0.53	0.46	0.53
2000	2000	0.53	0.60	0.54	0.61
2500	500	0.16	0.22	0.14	0.23
2500	1000	0.30	0.38	0.28	0.38
2500	1500	0.40	0.48	0.39	0.47
2500	2000	0.47	0.55	0.48	0.56

The convergence criterion is the value of the Taylor series at order 10

${\text{E}}\left[ {\tilde{r}_{{q_{\text{c}} ,\hat{q}_{\text{c}} }}^{2} } \right]$ is the expectation of the second approximation across the distribution of allele frequencies as given in Goddard [16]

Table 6 shows that the second Taylor series converges always when $\nu^{2} = 0.4$. The proposed approximation was generally biased upwards. This over-estimation of the precision was generally limited but increased as the number of markers and the reference population size decreased. The maximum over-estimation observed was 37.5 % (0.22 instead of 0.16 with a standard error less than 0.02). Based on the results in Table 5, it appears that when the first Taylor series converges, the proposed approximation is also slightly over-estimated. The expectation of the approximations, as given in formulae (5) and (6) are very close to the observations.

#### No LD and non-independence between reference and candidate population

The quality of the approximation was tested as above, by considering the case of a candidate having one of its parents in the reference population and all other individuals being unrelated. Tables 7 and 8, which summarize the results of the simulation, show that the second approximation is still the most efficient (systematic convergence of the Taylor series and consistency between first order approximation and its expectation). Again, an overestimation of about 20 % is observed with this approximation.

**Table 7:** Performances of the first approximation $\left( {\hat{r}_{{q_{\text{c}} ,\hat{q}_{\text{c}} }}^{2} } \right)$ when the parents of candidate belong to the reference population as a function of the number of markers (*n*_*M*_) and reference population size (*n*_*R*_), assuming *ν* ^2^ = 0.4

*n*_*M*_	*n*_*R*_	True value $\left( {{r}_{{q_{\text{c}} ,\hat{q}_{\text{c}} }}^{2} } \right)$	Approximation $\left( {\hat{r}_{{q_{\text{c}} ,\hat{q}_{\text{c}} }}^{2} } \right)$	10th order approximation	${\text{E}}\left[ {\hat{r}_{{q_{\text{c}} ,\hat{q}_{\text{c}} }}^{2} } \right]$
1000	500	0.37	0.42	0.58	0.47
1000	1000	0.56	0.73	2.47	0.82
1000	1500	0.65	0.95	2.50	1.04
1000	2000	0.72	1.17	2.52	1.26
1500	500	0.31	0.34	0.33	0.37
1500	1000	0.46	0.56	1.87	0.63
1500	1500	0.56	0.73	2.44	0.81
1500	2000	0.62	0.87	2.50	0.96
2000	500	0.27	0.29	0.27	0.32
2000	1000	0.40	0.46	0.89	0.52
2000	1500	0.50	0.62	2.24	0.69
2000	2000	0.57	0.76	2.48	0.84
2500	500	0.24	0.25	0.24	0.27
2500	1000	0.36	0.40	0.49	0.45
2500	1500	0.46	0.55	1.79	0.61
2500	2000	0.52	0.67	2.35	0.74

The convergence criterion is the value of the Taylor series at order 10

${\text{E}}\left[ {\hat{r}_{{q_{\text{c}} ,\hat{q}_{\text{c}} }}^{2} } \right]$ is the expectation of the first approximation across the distribution of allele frequencies as given in Goddard [16]

**Table 8:** Performances of the second approximation $\left( {\tilde{r}_{{q_{\text{c}} ,\hat{q}_{\text{c}} }}^{2} } \right)$ when the parents of the candidates belong to the reference population as a function of the number of markers (*n*_*M*_) and reference population size (*n*_*R*_), assuming *ν* ^2^ = 0.4

*n*_*M*_	*n*_*R*_	True value $\left( {{r}_{{q_{\text{c}} ,\hat{q}_{\text{c}} }}^{2} } \right)$	Approximation $\left( {\tilde{r}_{{q_{\text{c}} ,\hat{q}_{\text{c}} }}^{2} } \right)$	10th order approximation	${\text{E}}\left[ {\tilde{r}_{{q_{\text{c}} ,\hat{q}_{\text{c}} }}^{2} } \right]$
1000	500	0.37	0.46	0.35	0.46
1000	1000	0.53	0.60	0.54	0.61
1000	1500	0.64	0.70	0.65	0.69
1000	2000	0.71	0.75	0.72	0.75
1500	500	0.30	0.39	0.26	0.40
1500	1000	0.47	0.55	0.46	0.51
1500	1500	0.56	0.63	0.56	0.61
1500	2000	0.63	0.69	0.64	0.68
2000	500	0.27	0.36	0.22	0.35
2000	1000	0.40	0.49	0.38	0.48
2000	1500	0.50	0.58	0.50	0.56
2000	2000	0.57	0.64	0.57	0.62
2500	500	0.24	0.33	0.20	0.32
2500	1000	0.34	0.44	0.31	0.45
2500	1500	0.44	0.53	0.43	0.53
2500	2000	0.37	0.46	0.35	0.46

The convergence criterion is the value of the Taylor series at order 10

${\text{E}}\left[ {\tilde{r}_{{q_{\text{c}} ,\hat{q}_{\text{c}} }}^{2} } \right]$ is the expectation of the second approximation across the distribution of allele frequencies as given in Goddard [16]

#### Example of the use of the second approach

As an illustration of formula (3) different situations that differ in the relationships between the candidate and reference populations were compared. Coefficients of formula (3) were estimated using the elements in Table 3. An effective reference population size of 200, the genotyping of 10,000 markers and a heritability of 0.4 were assumed. Scenarios included no individuals related to the candidate in the reference population, its sire, both parents, 1–10 half-sibs (or uncles), and a combination of parental and half-sib information.

**Fig. 3:**
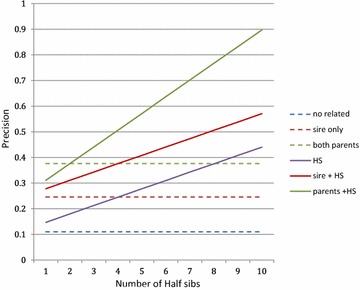
Example of approximated precision [from Eq. (3)] corresponding to various relations between the candidate and reference populations. ($n_{R} = 1000;  n_{M} = 10,000; \nu^{2} = 0.4$)

The results are in Fig. 3. The linearity of the precision increases with the number of half-sibs, which is consistent with the approximation, but unsatisfactory, as discussed below.

#### Equivalent number of independent loci

This number was computed using formula (8), for various effective population sizes ($N_{e} = 100 \;{\text{to}} \;1000$), heritabilities ($h^{2} = 0.1 \;{\text{to}}\; 0.5$), total numbers of loci ($n_{M} = 1000 \;{\text{to}}\; 10,000)$ and reference population sizes ($n_{R} = 1000 \;{\text{to}}\; 2500$).

**Fig. 4:**
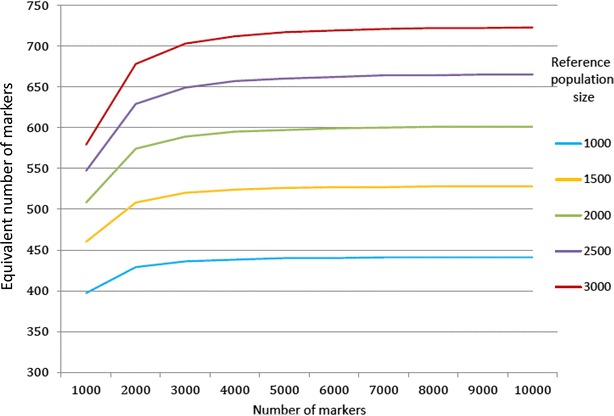
Number of equivalent markers [from Eq. (8)] as a function of the total number of markers (*n*_*M*_) and reference population size (*n*_*R*_). ($Ne = 200;  \nu^{2} = 0.4$)

Figure 4 shows how equivalent numbers of independent loci ($$ n_{{M_{i} }} $$) vary with the total number of markers ($$ n_{M} ) $$ and reference population size (*n*_*R*_). As *n*_*M*_ increases, the number $$ n_{{M_{i} }} $$ rapidly converges to a value which strongly depends on the size of the reference population size. This dependence on *n*_*R*_ of the equivalent number of independent loci does not exist in the Goddard’s effective number of loci and clearly shows the difference in nature between these concepts. Three phenomena, observed when considering the extreme case of two markers (see Additional file 5), explain this behavior:

1. The trace T of (E[**X**_**r**_^′^**X**_**r**_] + λ_β_**I**)^−1^ is a decreasing function of n_*r*_: as a consequence, the larger is the population size, the smaller is T, which is proportional to the marker effects conditional variances $v\left(\varvec{\beta}\right) - cov\left( {\varvec{\beta},{\mathbf{y}}} \right)v\left( \varvec{y} \right)^{ - 1} cov( {{\mathbf{y}},\varvec{\beta}})$) and the higher is the variance of the estimated molecular score (${\text{v}}\left( {q_{c} |{\mathbf{y}}} \right) = {\mathbf{x}}_{{\mathbf{c}}} cov\left( {\varvec{\beta},{\mathbf{y}}} \right)v\left( \varvec{y} \right)^{ - 1} cov\left( {{\mathbf{y}},\varvec{\beta}} \right){\mathbf{x}}^{\prime}_{{\mathbf{c}}} )$.
2. The trace T is always higher in the situation of LD than for independent markers $\left( {{\text{T}}_{LD} > {\text{T}}_{LE} } \right)$.
3. The rate of decrease is higher for T_*LD*_ than for T_*LE*_. On the whole, the reliability for a given number of observed markers corresponds to the reliability that is reached with a larger number of independent loci when the size of the reference population is larger.

**Fig. 5:**
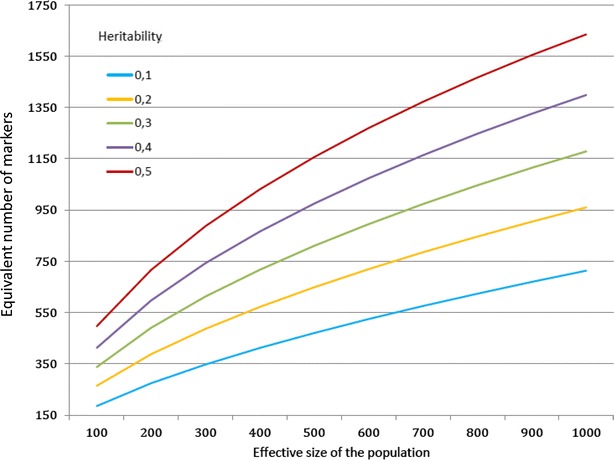
Number of equivalent markers [from Eq. (8)] as a function of the effective population size (*Ne*) and heritability (*v* ^2^) $\left( {n_{M} = 5000; n_{R} = 2000} \right)$

Figure 5 indicates that the equivalent number of independent loci increases with heritability and effective population size. This last observation was expected since with larger effective population sizes, the LD between two loci decreases and this increases the effective number of loci. The effect of heritability is less direct.

## Discussion

The objective of this paper was to explore approximations of the precision of genomic selection when the selection candidate has relatives in the reference population. Two approximations were developed and numerically compared.

These approximations were based on Taylor expansions of a matrix inverse **M**^−1^. In both cases, the initial matrix is the sum of the identity matrix and a perturbation (**M** = **I** + **E**). Convergence of these series is not guaranteed and depends on the behavior of the perturbation (**I** − **E** + **E**^2^ − **E**^3^ → (**I** + **E**)^−1^ if ${\mathbf{E}}^{\text{t}} \to 0$ when t → ∞). With the first approximation, derived from the appendix in [18], this convergence failed when the number of makers was too small (less than 1500 in our example) or the heritability was greater than 0.1. This was only observed when *ν*^2^ = 0.7 with the second approximation. This is fully consistent with the deviation to one of the largest eigenvalue of the **E** matrix.

The expectation of the proposed approximation, when data were simulated with the model corresponding to the hypotheses underlying their algebraic development, was very close to the mean value after 50 simulations. Thus, extremely fast estimation of the precision is possible, which allows intensive optimization and comparison of selection schemes.

When individuals are unrelated and markers are in linkage equilibrium, we obtain an estimation of the GEBV accuracy which differs from that of Goddard et al. [18]. This is surprising since that approach was said to be based on the Taylor approximation used here. Their formula may be obtained in a simpler way [see Additional file 6]. However, relaxing the assumption of “absence of between-individual relationship” is not straightforward using this approach.

A strong limit of our new approximation comes from the limitation to the first order term of the Taylor series. Deriving algebra was only possible at this stage. The side effect is that no genotypic covariance terms between reference individuals appear in this approximation. As a consequence, only the direct relationships between candidate and reference individuals play a role in the estimation, but not the structure within the reference population. This is unfortunate, because accuracies of genomic prediction are obviously affected by the construction of the reference population. Our last numerical example, in which there is a linear trend with the number of half-sibs, reveals this drawback: two half-sibs of the candidates are treated as unrelated and the information that they carry is just the double of that of a single half-sib. Future developments should focus on this limitation, for instance to derive the expectation of the **x**_**c**_**C**^−1^**B**^2^**x**_**c**_^′^ term.

**Fig. 6:**
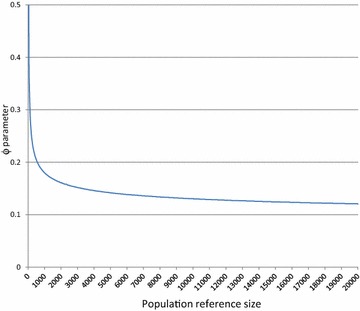
Parameter of the beta distribution ${\mathcal{B}} ( {\phi ,\phi })$ that best fits Godard’s distribution of allele frequencies

The U-shaped density function $f( p)$ of allele frequencies was defined as in [16]$.$ A Beta distribution ${\mathcal{B}}( {\phi_{a} ,\phi_{b} })$ for the allele frequencies was assumed by Gianola et al. [30], following Wright [34]. Assuming that the frequency distribution is centered on 0.5, *i.e.Φ*_*a*_ = *Φ*_*b*_ = *Φ*, this quantity *Φ* can be adjusted to fit the distribution of Goddard. Using the Chi^2^ test as a fitting option, we observed that the adjusted $\hat{\phi }$ rapidly decreased as the population size increased (Fig. 6), with a slower and slower evolution as the population size grew larger (with $n_{r} = 200{,}000$ the adjusted $\hat{\phi }$ is 0.9750000). Using a Beta distribution could give more generality to the results. If the expectation of τ and τ_2_ are easily derived from the moments generating function of Beta distribution (${\text{E}}[ {{\tau }}] = \frac{{n_{r} a}}{2a + 1}$ and ${\text{E}} [ {{{\tau }}_{2} } ] = n_{r} \frac{{4a^{2} + 16a + 18}}{{4a^{2} + 8a + 3}}$), deriving the expectation of parameters $\sum\nolimits_{m} {\rho_{m} }$, ∑_*m*_*ρ*_*m*_^2^ and $\sum\nolimits_{m} {\frac{{\rho_{m}^{2} }}{{\sigma_{m}^{2} }}}$ is not simple. However, these quantities are quite easily obtained by numerical integration. Thus, adjusting a Beta distribution to observed allele frequencies and numerically computing formula (3) parameters would be a feasible and more versatile implementation of our second genomic precision approximation.

Our work focused on the BLUP precision of the molecular score $r^{2} \left( {q_{ci} ,\hat{q}_{ci} |{\mathbf{X}}} \right) = \frac{{v\left( {\hat{q}_{ci} } \right)}}{{v\left( {q_{ci} } \right)}}$ but left aside the proportion of the genetic variance that is captured by the markers $\left( {\frac{{v ( {q_{ci} }  )}}{{v ( {g_{ci} } )}}} \right)$. This last term could be treated as in Goddard et al. [18]: $\frac{{v\left( {q_{ci} } \right)}}{{v ( {g_{ci} })}} = b = \frac{{n_{M} }}{{n_{M} + M_{e} }}$ with *M*_*e*_ the number of independent segments. As noted in the section on the general framework, the quantity $\frac{{v ( {\hat{q}_{ci} } )}}{{v ( {g_{ci} } )}} = b \times r ( {q_{ci} ,\hat{q}_{ci} |{\mathbf{X}}}  )$ is only an approximation of these GEBV reliabilities i.e. $r^{2} \left( {g_{ci} ,\hat{q}_{ci} |{\mathbf{X}}} \right) = \frac{{cov^{2} \left( {g_{ci} ,\hat{q}_{ci} |{\mathbf{X}}} \right)}}{{v\left( {g_{ci} |{\mathbf{X}}} \right)v\left( {\hat{q}_{ci} |{\mathbf{X}}} \right)}}$. Equality between those quantities is obtained when ${\mathbf{X}} = {\mathbf{W}}$(identity between statistical and genetical models), a condition assumed in Goddard [16] where markers and QTL are modeled as a series of uncorrelated pairs.

All the developments shown in this paper are based on the hypothesis that the reliability of GEBV based on non-independent markers for a trait controlled by $n_{Q}$ QTL that are in incomplete LD with the markers can be approached by the reliability of GEBV when there are *n*_*M*_ independent segments that carry a single QTL in LD with a single marker. A few difficulties arose when applying this approach proposed by Goddard [16]. How many independent markers should be considered? The reasoning in Goddard [16] was based on the idea of an effective number of loci (*M*_*e*_) corresponding to a given variance of realized relationships. Here, we proposed the alternative equivalent number of independent loci (*M*_*i*_) which corresponds to a given reliability. We showed that this *M*_*i*_ number depends on the size of the reference population and on heritability, a dependence that does not occur with *M*_*e*_. If we invert the argument, controlling the level of realized relationships variance with the effective number of loci (*M*_*e*_) does not seem to be a good approach to control the estimated GEBV reliability.

As detailed by Hayes et al. [17], the effective number of independent chromosome segments depends on the population structure. The higher is the mean relationship level, the smaller is this effective number. However, we suggest the use of this number as estimated from a set of unrelated individuals, or of its expectation prior to any observation, assuming independence between individuals. Without formal proof, the idea was that long-term LD was considered by using an effective (or equivalent) number of independent loci while short-term non-independence was taken into account with our formalization of the matrix’s expectations that is developed in Additional file 1. A complete proof of the procedure is still needed.

Regardless of the definition of $M_{e}$ or *M*_*i*_, there is no reason that the number of independent loci must equal the number of QTL, which is unknown, contrary to the hypothesis about pairs of marker-QTL (in practice, since the QTL effects are random variables, many segments will only have very small effects on the trait, thus simulating the more likely situation of a limited number of “real” QTL). Equating ${\mathbf{X}}$ and **W** as well as σ_β_^2^ and σ_α_^2^ has no clear justification. The variance $v\left( {\hat{q}_{c} |{\mathbf{X}}} \right)$ of the molecular score should not be *σ*_β_^2^**x**_**c**_**X**_**r**_^′^(**X**_**r**_**X**_**r**_^′^ + **I**λ_β_)^−1^**X**_**r**_**x**_**c**_^′^ but *σ*_α_^2^**x**_**c**_**X**_**r**_^′^(**X**_**r**_**X**_**r**_^′^ + **I**λ_β_)^−1^(**W**_**r**_**W**_**r**_^′^ + **I**λ_α_)(**X**_**r**_**X**_**r**_^′^ + **I**λ_β_)^−1^**X**_**r**_**x**_**c**_^′^. This other formula assembles two sets of unknown parameters: the variances σ_α_^2^ and σ_β_^2^, and the genotypes** X** and **W**. It is often assumed that ${{\sigma }}_{{{\upbeta }}}^{2} = \sigma_{g}^{2} /\left( {n_{M} \bar{\tau }} \right)$ (e.g. [1]), which results in an overestimation of the ${{\uplambda }}_{{{\upbeta }}}$ parameter since LD is not considered. Working on the number of independent loci ($M_{e}\, {\text{or}}\,M_{i}$) apparently solves this difficulty. The QTL variance ${{\sigma }}_{{{\alpha }}}^{2} = \sigma_{g}^{2} /\left( {n_{Q} \bar{\tau }} \right)$ could be derived based on a hypothesis about the number of QTL. The situation is more difficult for the genotype matrices since the **W**_**r**_ matrix is not observed.

If the framework considered so far (*n*_*M*_ markers-QTL pairs with strong LD within pairs and no LD between pairs) is partly retained, a slight improvement is possible considering the element b of the genetic variability explained by SNPs. The idea would be to replace, in the formulae used in this paper, σ_q_^2^ by b × σ_g_^2^. Element b can be derived by considering that the markers’ (**β**) and QTL’ (**α**) effects are fixed in the genetic and statistical models. Leaving aside the singularity of **X**_**r**_^′^**X**_**r**_ when the number of SNPs is large, the marker effects are now estimated by ${\hat{\varvec{\upbeta }}} = \left( {{\mathbf{X}}^{\prime}_{{\mathbf{r}}} {\mathbf{X}}_{{\mathbf{r}}} } \right)^{ - 1} {\mathbf{X}}^{\prime}_{{\mathbf{r} }}{\mathbf{y}}$ and the molecular score defined as ${\hat q}= {\mathbf{X}}_{{\mathbf{r}}} {\hat{\varvec{\upbeta }}}$, while the genetic value was **g** = **W**_**r**_**α**. Given the genotype matrices, the sample genetic variability is *v*_*g*_ = **α**^**′**^**W**_**r**_^′^**W**_**r**_**α** and the sample molecular score variability **y**^**′**^**X**_**r**_(**X**_**r**_^′^**X**_**r**_)^−1^**X**_**r**_^′^**y** with an expectation *v*_*q*_ = **α**^′^**W**_**r**_^′^**X**_**r**_(**X**_**r**_^′^**X**_**r**_)^−1^**X**_**r**_^′^**W**_**r**_**α**. The part of the genetic variability explained by the SNPs is the ratio b = *v*_*q*_/*v*_*g*_.

Expectations of the matrices’ product elements $\left\{ {{\mathbf{X}}^{\prime}_{{\mathbf{r}}} {\mathbf{X}}_{{\mathbf{r}}} } \right\}_{{\varvec{ml}}}$ are $2n_{r} \Updelta_{ml}$ off diagonal and 2*n*_*r*_*p*_*m*_(1 − *p*_*m*_) = *n*_*r*_*σ*_*m*_^2^ in the diagonal, with similar expressions for ${\mathbf{W^{\prime}_{\mathbf{r}}}} {\mathbf{X}}_{{\mathbf{r}}}$ and **W**_**r**_^′^**W**_**r**_ elements.

Following Goddard [16], approximating expectations of the matrices’ functions by the function of their expectation, and assuming that (1) markers are independent, (2) each QTL *q* is in LD with only one marker *m*(*q*), with a LD value $\Updelta_{qm\left( q \right)}$, and (3) individuals are unrelated: *v*_*g*_ = *n*_*r*_ ∑ _*q*_*α*_*q*_^2^*σ*_*q*_^2^, we get $v_{q} \sim 4n_{r} \sum\nolimits_{q} {\frac{{\Updelta_{qm\left( q \right)}^{2} }}{{\sigma_{m\left( q \right)}^{2} }}\alpha_{q}^{2} } = n_{r} \sum\nolimits_{q} {r_{qm\left( q \right)}^{2} \alpha_{q}^{2} \sigma_{q}^{2} }$, and ${\text{b}} = \frac{{\mathop \sum \nolimits_{q} r_{qm\left( q \right)}^{2} \alpha_{q}^{2} \sigma_{q}^{2} }}{{\mathop \sum \nolimits_{q} \alpha_{q}^{2} \sigma_{q}^{2} }}$, corresponding to Eq. (4) in [16].

The ratio b is the weighted mean of LD *r*^2^. Unfortunately, neither *α*_*q*_^2^ nor *σ*_*q*_^2^ are known. The unweighted mean $\frac{{\mathop \sum \nolimits_{q} r_{qm\left( q \right)}^{2} }}{{n_{q} }} = \bar{r}^{2}$ may be a fruitful approximation. Following Sved [33], the expectation of $r_{qm\left( q \right)}^{2}$ is $\frac{1}{{1 + 4N_{e} c}}$ with *c* being the distance, in Morgan, between the QTL and its marker. Let *L* be the total length of the genome, and assume an equal distance *L*/*n*_*M*_ between each successive marker ${\text{b}}\sim \int_{0}^{{L/2n_{M} }} {\frac{1}{{1 + 4N_{e} c}}\frac{1}{{{L \mathord{\left/ {\vphantom {L {2n_{M} }}} \right. \kern-0pt} {2n_{M} }}}}dc = \frac{{n_{M} }}{{2N_{e} L}}\left[ {{ \log }\left( {1 + {{2N_{e} L} \mathord{\left/ {\vphantom {{2N_{e} L} {n_{M} }}} \right. \kern-0pt} {n_{M} }}} \right)} \right]}$.

The expectation of the reliability ${\text{E}}\left[ {r_{{q_{\text{c}} ,\hat{q}_{\text{c}} }}^{2} } \right]$, which is a ratio of variances ${\text{E}}_{{\mathbf{X}}} \left[ {{\text{v}}\left( {\hat{q}_{c} |{\mathbf{X}}} \right)/{\text{v}}\left( {q_{\text{c}} |{\mathbf{X}}} \right)} \right]$ was approximated by the ratio of the variance expectations ${\text{E}}_{{\mathbf{X}}} \left[ {{\text{v}}\left( {\hat{q}_{c} |{\mathbf{X}}} \right)} \right]/{\text{E}}_{{\mathbf{X}}} \left[ {{\text{v}}\left( {q_{\text{c}} |{\mathbf{X}}} \right)} \right]$. The usual second degree approximation (E[*N*/*D*] = E[*N*]/E[*D*] − *cov*[*N*, *D*]/E^2^[*D*] + *v*[*D*]E[*N*]/E^3^[*D*]) could not be used here due to algebra complexity. However, in the case of unrelated individuals and independent markers, numerical evaluation of the difference between exact and approximated results for various reference population sizes and numbers of markers shows a very small underestimation of the reliability (Table 9).

**Table 9:** Expectation of the ratio of variances vs. the ratio of the variance expectations considering different reference population sizes and numbers of markers (*ν* ^2^ = 0.4, 50 simulations)

*n*_*r*_	*n*_*M*_	${\text{E}}\left[ {{\text{v}}\left( {\hat{q}_{\text{c}} } \right)/{\text{v}}\left( {q_{\text{c}} } \right)} \right]$	${\text{E}}\left[ {{\text{v}}\left( {\hat{q}_{\text{c}} } \right)} \right]/{\text{E}}\left[ {{\text{v}}\left( {q_{\text{c}} } \right)} \right]$
500	1000	0.403	0.401
1000	1000	0.726	0.725
1500	1000	1.010	1.008
2000	1000	1.212	1.212
500	1500	0.270	0.269
1000	1500	0.535	0.534
1500	1500	0.753	0.753
2000	1500	0.944	0.944
500	2000	0.213	0.213
1000	2000	0.414	0.413
1500	2000	0.597	0.597
2000	2000	0.760	0.759
500	2500	0.175	0.175
1000	2500	0.349	0.348
1500	2500	0.515	0.514
2000	2500	0.670	0.669

The theory presented here was developed by considering a single selection candidate. When candidates are diversely related to the reference population, as suggested in Goddard et al. [18], the candidates should be examined one by one. Moreover, non-independence between candidates should be considered. A further step towards the modeling of genomic selection could be an approximation of the mean genetic values of selected individuals when GEBV reliabilities are heterogeneous.

A few other hypotheses were made in this paper, including additivity and *i.i.d.* of QTL effects, and the use of GBLUP. As long as the objective is to model and optimize breeding plans, only relative values are interesting and we assumed that these hypotheses were not critical.

## Conclusions

The objective of this paper was to provide a further step towards the development of deterministic models that describe genomic breeding plans. Such deterministic models carry low computational burden and thus allow design optimization through intensive numerical exploration.

We proposed two alternative approximations of the estimation of GEBV reliability in the case of non-independence between candidate and reference populations. Both were derived from the Taylor series heuristic approach suggested by Goddard [16]. A numerical exploration of their properties showed that the series were not equivalent in terms of convergence to the exact reliability, that the approximations may overestimate GEBV precision and that they perfectly converged toward their theoretical expectations.

Formulae derived for these approximations were simple to handle in the case of independent markers. A few parameters that describe the markers’ genotypic variability (allele frequencies, linkage disequilibrium) can be estimated from genomic data corresponding to the population of interest or estimated after assumption about their distribution.

When markers are not in linkage equilibrium (*i.e.* there is LD), replacing the real number of markers and QTL by an effective or equivalent number of independent loci, as proposed by Goddard [16] and Hayes et al. [17], is a practical solution. Research efforts are still needed to overcome some strong limits of this approach.

## Additional files

10.1186/s12711-016-0183-3 Computation of ${\text{E}}\left[ {{\text{X}}_{\text{im}}^{{{\text{d}}_{\text{i}} }} {\text{X}}_{\text{jm}}^{{{\text{d}}_{\text{j}} }} \cdots {\text{X}}_{\text{Km}}^{{{\text{d}}_{\text{K}} }} } \right]$ as a function of between-chromosome identity coefficients, in the case of independent markers. Using an extension of the identity coefficients theory, the document shows how to compute the elements of the expectation E[XX^′^TXX^′^] (*i.e.* ${\text{E}}\left[ {X_{i} X_{j} } \right],  {\text{E}}\left[ {X_{i} X_{j}^{2} } \right],  {\text{E}}\left[ {X_{i} X_{j}^{3} } \right], {\text{E}}\left[ {X_{i}^{2} X_{j}^{2} } \right],$ ${\text{E}}\left[ {X_{i} X_{j} X_{k}^{2} } \right]$ and E[*X*_*i*_ *X*_*j*_ *X*_*k*_ *X*_*l*_]) when markers are independent and individuals are related.
10.1186/s12711-016-0183-3 Expectations of $\mathop \sum \limits_{m} \sigma_{m}^{2} , \mathop \sum \limits_{m} \sigma_{m}^{4} , \mathop \sum \limits_{m} \rho_{m}$, ∑_*m*_ *ρ*_*m*_^2^, ∑_*m*_ *ρ*_*m*_^2^/*σ*_*m*_^2^ and ∑_*m*_1/*σ*_*m*_^2^. The expectations of the listed quantities are computed assuming either a U-shaped or a uniform distribution of allele frequencies.
10.1186/s12711-016-0183-3 Precision formulae when the candidate is related to reference individuals. The approximated formulae derived in the main text are applied to the case of a candidate for which the sire belongs to the reference population.
10.1186/s12711-016-0183-3 Equivalent numbers of independent loci. (1) equation of the equivalent number of independent loci which gives the precision ${\text{E}}\left[ {r_{{q_{\text{c}} ,\hat{q}_{c} }}^{2} } \right]\sim \frac{{{\text{E}}_{{\mathbf{X}}} \left[ {{\text{v}}(\hat{q}_{c} |{\mathbf{X}})} \right]}}{{{\text{E}}_{{\mathbf{X}}} \left[ {{\text{v}}\left( {q_{\text{c}} |{\mathbf{X}}} \right)} \right]}}$ obtained with the total number of non-independent markers; (2) simple approximation in a very simplified situation; (3) relations between number of independent markers and size of the reference population.
10.1186/s12711-016-0183-3 The case of markers in linkage disequilibrium. Derivation of the crossed terms expectation of genomic values (E[*x*_*cl*_ *x*_*cm*_ *X*_*il*_ *X*_*im*_]) in the situation of LD between loci *l* and *m*. Many examples are given for diverse situations.
10.1186/s12711-016-0183-3 Another demonstration of Goddard et al. [18] accuracy. A complete demonstration is given using the notations of the present paper.
