# Supplementary material for: Approximated prediction of genomic selection accuracy when reference and candidate populations are related
Source: Genet Sel Evol. 2016 Mar 3;48:18. doi: 10.1186/s12711-016-0183-3 (PMC4778372; doi:10.1186/s12711-016-0183-3)

Additional file 1 : Computation of $\mathrm{E}\left[X_{im}^{d_i} X_{jm}^{d_j} \cdots X_{Km}^{d_K}\right]$ as a function of between chromosomes identity coefficients, in the case of independent markers.

Principles

Derivation of $\mathrm{E}[\mathbf{XX}'\mathbf{TXX}']$ elements are simplified using three properties:

- $\mathrm{E}\left[(\sum_m X_{im}X_{km})(\sum_m X_{jm}X_{lm})\right] = \sum_\mathrm{m} \mathrm{E}\left[X_{im}X_{km}X_{jm}X_{lm}\right] + (\sum_\mathrm{m} \mathrm{E}[X_{im}X_{km}])(\sum_\mathrm{m} \mathrm{E}\left[X_{jm}X_{lm}\right]) - \sum_\mathrm{m}\left(\mathrm{E}[X_{im}X_{km}]\mathrm{E}\left[X_{jm}X_{lm}\right]\right)$
- We will demonstrate in this supplementary material that, at least for the K (1 to 4) and d_k (1 to 4) values present in our derivations, any expectation $\mathrm{E}\left[X_{im}^{d_i} X_{jm}^{d_j} \cdots X_{Km}^{d_K}\right]$ with $\sum_i d_i$ even can be written as
$$p_m(1-p_m)\alpha_{ij\cdots K}^{d_i d_j \cdots d_K} - [p_m(1-p_m)]^2 \gamma_{ij\cdots K}^{d_i d_j \cdots d_K}$$
where parameters $\alpha_{ij\cdots K}^{d_i d_j \cdots d_K}$ and $\gamma_{ij\cdots K}^{d_i d_j \cdots d_K}$ are functions of identity states probabilities between gametes of $ij \cdots K$ individuals at marker m.
- When individual are repeated (*e.g.* $i = j$), $\mathrm{E}\left[\cdots X_{im}^{d_i} X_{jm}^{d_j} \cdots\right] = \mathrm{E}\left[\cdots X_{im}^{d_i + d_j} \cdots\right]$.

Let $= \sum_m 2p_m(1-p_m)$, $\tau_2 = \sum_q [2p_m(1-p_m)]^2$ and a_{ij} the coancestry coefficient between individuals i and j

$$\sum_\mathrm{m} \mathrm{E}\left[X_{im}X_{km}X_{jm}X_{lm}\right] = \frac{1}{2}\tau\alpha_{\mathrm{ijkl}}^{1111} - \frac{1}{4}\tau_2\gamma_{\mathrm{ijkl}}^{1111}$$
$$\sum_\mathrm{m} \mathrm{E}[X_{im}X_{km}] = \frac{1}{2}\tau\alpha_{ik}^{11} - \frac{1}{4}\tau_2\gamma_{ik}^{11} = 2a_{ik}\tau \text{ and } \sum_\mathrm{m} \mathrm{E}\left[X_{jm}X_{lm}\right] = 2a_{jl}\tau$$
$$\sum_\mathrm{m}\left(\mathrm{E}[X_{im}X_{km}]\mathrm{E}\left[X_{jm}X_{lm}\right]\right) = 4a_{ik}a_{jl}\tau_2$$

Thus $\{\mathrm{E}[\mathbf{XX}'\mathbf{TXX}']\}_\mathrm{ij} = \sum_l \sum_k t_{kl}\left(\frac{1}{2}\tau\alpha_{ijkl}^{1111} - \frac{1}{4}\tau_2\gamma_{ijkl}^{1111} + 4a_{ik}a_{jl}[\tau^2 - \tau_2]\right)$. Elements of this summation are computed considering the third property given above.

Demonstration.

Let $S_{c_1 c_2 \cdots c_{n_C}}$ the identity state between n_C chromosomes at a given locus. This is an extension of the identity coefficients [35-37]. We do not consider pairs of chromosomes of two individuals, but a set of n_C chromosomes which may or not belong to different individuals. For instance, c_1 could mean the paternal allele at locus m for individual i (it will be noted in this case c_1 =is). The figure SM1-1 represents the possible states, depending on the number of chromosomes. When $n_C = 2$ only two identity states are possible: locus are IBD ($S_{c_1 c_2} = 1$) or not IBD ($S_{c_1 c_2} = 2$). When $n_C = 3$ five identity states are possible and when there are 4 chromosomes, we find back the 15 classical identity states .

The codification for the genotypes are $X_{im} = (0, 1 \text{ or } 2) - 2p_m$. (It is equivalent to $X_{im} = g_{ims} + g_{imd}$ where g_{ims} and g_{imd} are the "values" of the alleles transmitted to individual i by its sire and its dam, with g_{ims} and $g_{imd} = (0 \; or \; 1) - p_m$. As we only consider one locus in the following derivation the m indice will be omitted : $X_i = g_{is} + g_{id}$ and g_{is} and $g_{id} = (0 \; or \; 1) - p$.

Different situations were encountered for the product $\mathrm{E}\left[X_i^{d_i} X_j^{d_j} \cdots X_K^{d_K}\right]$

$$\mathrm{E}\left[X_i X_j\right] \; i \neq j$$

$$\mathrm{E}\left[X_i X_j^3\right] \; i \neq j$$

$$\mathrm{E}\left[X_i^2 X_j^2\right]\ i \neq j$$

$$\mathrm{E}\left[X_i X_j X_k^2\right]\ i \neq j \neq k$$

$$\mathrm{E}\left[X_i X_j X_k X_l\right]\ i \neq j \neq k \neq l$$

In all case, we will decompose the X genotypes in their g values

$$\mathrm{E}\left[X_i^{d_i} X_j^{d_j} \cdots\right] = \sum_{S_{is,id,js,jd,\cdots}} p(S_{S_{is,id,js,jd,\dots,\cdots}})\ \mathrm{E}\left[(g_{is}+g_{id})^{d_i}(g_{js}+g_{jd})^{d_j} \cdots |S_{S_{is,id,js,jd,\dots,\cdots}}\right]$$

This formula turns to be the weighted sum of elements such as $\mathrm{E}\left[g_{is}{}^{d_i} g_{js}{}^{d_j-1} g_{jd} \cdots |S_{is,js,jd\cdots}\right]$ which are very simple to derive from the allele frequency p.

Computations are simplified by the fact that those expectations are null when one of the elements of the product, here say g_{jd} , is unic and the state $S_{is,js,jd\cdots}$ such that the jd locus is not IBD with any other locus.

1) $\mathrm{E}\left[X_i X_j\right]\ i \neq j$

$$\mathrm{E}\left[X_i X_j\right] = \mathrm{E}\left[g_{is} g_{js}\right] + \mathrm{E}\left[g_{is} g_{jd}\right] + \mathrm{E}\left[g_{id} g_{js}\right] + \mathrm{E}\left[g_{id} g_{jd}\right]$$

$\mathrm{E}\left[g_{is} g_{js}\right] = p\left(S_{is,js}=1\right)\{(1-p)(0-p)^2 + p(1-p)^2\} + p\left(S_{is,js}=0\right)\{(1-p)^2(0-p)^2 + 2p(1-p)(1-p)(0-p) + p^2(1-p)^2\}$

$\mathrm{E}\left[g_{is} g_{js}\right] = p\left(S_{is,js}=1\right)\{p(1-p)\}$

$$\boxed{\mathrm{E}\left[X_i X_j\right] = p(1-p)\left[p\left(S_{is,js}=1\right) + p\left(S_{is,jd}=1\right) + p\left(S_{id,js}=1\right) + p\left(S_{id,jd}=1\right)\right]}$$

It must be noted that the event $S_{is,js}=1$ etc. corresponds to the classical identity states 1, 2, 4, 9, 10, that is $p\left(S_{is,js}=1\right) = \delta_1 + \delta_2 + \delta_4 + \delta_5 + \delta_9 + \delta_{10}$; that $S_{is,jd}=1$ corresponds to the 1,3,4,12,13 etc . The results being that

$\mathrm{E}\left[X_i X_j\right] = p(1-p)[4\delta_1 + 2[\delta_2 + \delta_3 + \delta_4 + \delta_5 + \delta_9 + \delta_{12}] + \delta_{10} + \delta_{11} + \delta_{13} + \delta_{14}] = 4p(1-p)a_{ij}$, as expected

$$\boxed{\mathrm{E}\left[X_i X_j\right] = 4p(1-p)a_{ij}}$$

2) $\mathrm{E}\left[X_i X_j^3\right]\ i \neq j$

$\mathrm{E}\left[X_i X_j^3\right] = \mathrm{E}\left[(g_{is}+g_{id})(g_{js}+g_{jd})^3\right] = \mathrm{E}\left[g_{is} g_{js}^3\right] + 3\mathrm{E}\left[g_{is} g_{js}^2 g_{jd}\right] + 3\mathrm{E}\left[g_{is} g_{js} g_{jd}^2\right] + \mathrm{E}\left[g_{is} g_{jd}^3\right] + \mathrm{E}\left[g_{id} g_{js}^3\right] + 3\mathrm{E}\left[g_{id} g_{js}^2 g_{jd}\right] + 3\mathrm{E}\left[g_{id} g_{js} g_{jd}^2\right] + \mathrm{E}\left[g_{id} g_{jd}^3\right]$

$\mathrm{E}\left[g_{is} g_{js}^3\right] = p\left(S_{is,js}=1\right)\{(1-p)(0-p)^4 + p(1-p)^4\}$=$p\left(S_{is,js}=1\right)\{p(1-p)[1-3p(1-p)]\}$

$\mathrm{E}\left[g_{is} g_{js}^2 g_{jd}\right] = p\left(S_{is,js,jd}=1\right)\{(1-p)(0-p)^4 + p(1-p)^4\} + p\left(S_{is,js,jd}=4\right)\{(1-p)^2(0-p)^4 + 2p(1-p)(0-p)^2(1-p)^2 + p^2(1-p)^4\} = p\left(S_{is,js,jd}=1\right)\{p(1-p)[1-3p]\} + p\left(S_{is,js,jd}=4\right)[p(1-p)]^2$

$\mathrm{E}\left[X_i X_j^3\right] = p(1-p)[1-3p(1-p)]\left[p\left(S_{is,js}=1\right) + p\left(S_{is,jd}=1\right) + p\left(S_{id,js}=1\right) + p\left(S_{id,jd}=1\right) + 6p\left(S_{is,js,jd}=1\right) + 6p\left(S_{id,js,jd}=1\right)\right] + [p(1-p)]^2\left[3p\left(S_{is,js,jd}=4\right) + 3p\left(S_{is,js,jd}=2\right) + 3p\left(S_{id,js,jd}=4\right) + 3p\left(S_{id,js,jd}=2\right)\right]$

	S1	S2	S3	S4	S5	S6	S7	S8	S9	S10	S11	S12	S13	S14	S15	X
$S_{is,js} = 1$	X	X		X					X	X						1
$S_{is,jd} = 1$	X		X	X								X	X			1
$S_{id,js} = 1$	X	X			X							X		X		1
$S_{id,jd} = 1$	X		X		X				X		X					1
$S_{is,js,jd} = 1$	X			X												6
$S_{id,js,jd} = 1$	X				X											6
$S_{is,js,jd} = 4$			X									X	X			3
$S_{is,js,jd} = 2$		X							X	X						3
$S_{id,js,jd} = 4$			X						X		X					3
$S_{id,js,jd} = 2$		X										X		X		3

$$\mathrm{E}\left[X_i X_j^3\right] = p(1-p)[1-3p(1-p)][16\delta_1 + 2(\delta_2+\delta_3) + 8(\delta_4+\delta_5) + 2(\delta_9+\delta_{12}) + \delta_{10} + \delta_{11} + \delta_{13} + \delta_{14}] + [p(1-p)]^2[6(\delta_2+\delta_3+\delta_9+\delta_{12}) + 3(\delta_{10}+\delta_{11}+\delta_{13}+\delta_{14})]$$

3) $\mathrm{E}\left[X_i^2 X_j^2\right]$ $i \neq j$

$\mathrm{E}\left[X_i^2 X_j^2\right] = \mathrm{E}\left[(g_{is}+g_{id})^2(g_{js}+g_{jd})^2\right] = \mathrm{E}\left[g_{is}^2 g_{js}^2\right] + \mathrm{E}\left[g_{is}^2 g_{jd}^2\right] + \mathrm{E}\left[g_{id}^2 g_{js}^2\right] + \mathrm{E}\left[g_{id}^2 g_{jd}^2\right] + 2\mathrm{E}\left[g_{is}^2 g_{js} g_{jd}\right] + 2\mathrm{E}\left[g_{id}^2 g_{js} g_{jd}\right] + 2\mathrm{E}\left[g_{is} g_{id} g_{js}^2\right] + 2\mathrm{E}\left[g_{is} g_{id} g_{jd}^2\right] + 4\mathrm{E}\left[g_{is} g_{id} g_{js} g_{jd}\right]$

$\mathrm{E}\left[g_{is}^2 g_{js}^2\right] = p(S_{is,js}=1)\{(1-p)(0-p)^4 + p(1-p)^4\} + p(S_{is,js}=2)\{(1-p)^2(0-p)^4 + 2p(1-p)(1-p)^2(0-p)^2 + p^2(1-p)^4\} = p(S_{is,js}=1)\{p(1-p)[p^3+(1-p)^3]\} + p(S_{is,js}=2)[p(1-p)]^2$

$\mathrm{E}\left[g_{is}^2 g_{js} g_{jd}\right] = p(S_{is,js,jd}=1)\{(1-p)(0-p)^4 + p(1-p)^4\} + p(S_{is,js,jd}=3)\{(1-p)^2(0-p)^4 + 2p(1-p)(1-p)^2(0-p)^2 + p^2(1-p)^4\}$ The state $S_{is,js,jd}=3$ corresponds to g_{js} IBD to g_{jd} and g_{is} not IBD to the others. In the other states we have terms like $\mathrm{E}\left[g_{jd}\right]=0$ in the conditional expectation $\mathrm{E}\left[g_{is}^2 g_{js} g_{jd} | S_{is,js,jd}\right]$.

$\mathrm{E}\left[g_{is}^2 g_{js} g_{jd}\right] = p(S_{is,js,jd}=1)\{p(1-p)[p^3+(1-p)^3]\} + p(S_{is,js,jd}=3)[p(1-p)]^2$

$\mathrm{E}\left[g_{is} g_{id} g_{js} g_{jd}\right] = p(S_{is,id,js,jd}=1)\{(1-p)(0-p)^4 + p(1-p)^4\} + \left[p(S_{is,id,js,jd}=6) + p(S_{is,id,js,jd}=9) + p(S_{is,id,js,jd}=12)\right]\{(1-p)^2(0-p)^4 + 2p(1-p)(1-p)^2(0-p)^2 + p^2(1-p)^4\}$

$\mathrm{E}\left[g_{is} g_{id} g_{js} g_{jd}\right] = p(S_{is,id,js,jd}=1)\{p(1-p)[p^3+(1-p)^3]\} + \left[p(S_{is,id,js,jd}=6) + p(S_{is,id,js,jd}=9) + p(S_{is,id,js,jd}=12)\right][p(1-p)]^2$

Finally

$$\begin{aligned}\mathrm{E}\left[X_i^2 X_j^2\right] = {} & \{p(1-p)[p^3+(1-p)^3]\}[p(S_{is,js}=1) + p(S_{is,jd}=1) + p(S_{id,js}=1) \\ & + p(S_{id,jd}=1) + 2p(S_{is,js,jd}=1) + 2p(S_{id,js,jd}=1) + 2p(S_{is,id,js}=1) \\ & + 2p(S_{is,id,jd}=1) + 4p(S_{is,id,js,jd}=1)] \\ & + [p(1-p)]^2\{p(S_{is,js}=2) + p(S_{is,jd}=2) + p(S_{id,js}=2) + p(S_{id,jd}=2) \\ & + 2p(S_{is,js,jd}=3) + 2p(S_{id,js,jd}=3) + 2p(S_{is,id,js}=2) + 2p(S_{is,id,jd}=2) \\ & + 4p(S_{is,id,js,jd}=6) + 4p(S_{is,id,js,jd}=9) + 4p(S_{is,id,js,jd}=12)\}\end{aligned}$$

	S1	S2	S3	S4	S5	S6	S7	S8	S9	S10	S11	S12	S13	S14	S15	X

$S_{is,js} = 1$	X	X		X					X	X						1
$S_{is,jd} = 1$	X		X	X								X	X			1
$S_{id,js} = 1$	X	X			X							X		X		1
$S_{id,jd} = 1$	X		X		X				X		X					1
$S_{is,js,jd} = 1$	X			X												2
$S_{id,js,jd} = 1$	X				X											2
$S_{is,id,js} = 1$	X	X														2
$S_{is,id,jd} = 1$	X		X													2
$S_{is,id,js,jd} = 1$	X															4
$S_{is,js} = 2$			X		X	X	X	X			X	X	X	X	X	1
$S_{is,jd} = 2$		X			X	X	X	X	X	X	X			X	X	1
$S_{id,js} = 2$			X	X		X	X	X	X	X	X		X		X	1
$S_{id,jd} = 2$		X		X		X	X	X		X		X	X	X	X	1
$S_{is,js,jd} = 3$					X	X		X								2
$S_{id,js,jd} = 3$				X		X		X								2
$S_{is,id,js} = 2$			X			X	X									2
$S_{is,id,jd} = 2$		X				X	X									2
$S_{is,id,js,jd} = 6$						X										4
$S_{is,id,js,jd} = 9$									X							4
$S_{is,id,js,jd} = 12$												X				4

$$\mathrm{E}\left[X_i^2 X_j^2\right] = \left[p(1-p)[1-3p(1-p)]\right]\{16\delta_1 + 4(\delta_2+\delta_3+\delta_4+\delta_5) + 2(\delta_9+\delta_{12}) + \delta_{10} + \delta_{11} + \delta_{13} + \delta_{14}\} + [p(1-p)]^2\{4(\delta_2+\delta_3+\delta_4+\delta_5+\delta_{15}) + 16\delta_6 + 8(\delta_7+\delta_8) + 6(\delta_9+\delta_{12}) + 3(\delta_{10}+\delta_{11}+\delta_{13}+\delta_{14}))\}$$

4) $\mathrm{E}\left[X_i X_j X_k^2\right]\ i \neq j \neq k$

$$\mathrm{E}\left[X_i X_j X_k^2\right] = \mathrm{E}\left[(g_{is}+g_{id})(g_{js}+g_{jd})(g_{ks}+g_{kd})^2\right] = \mathrm{E}\left[g_{is}g_{js}g_{ks}^2\right] + \mathrm{E}\left[g_{is}g_{jd}g_{ks}^2\right] + \mathrm{E}\left[g_{id}g_{js}g_{ks}^2\right] + \mathrm{E}\left[g_{id}g_{jd}g_{ks}^2\right] + \mathrm{E}\left[g_{is}g_{js}g_{kd}^2\right] + \mathrm{E}\left[g_{is}g_{jd}g_{kd}^2\right] + \mathrm{E}\left[g_{id}g_{js}g_{kd}^2\right] + \mathrm{E}\left[g_{id}g_{jd}g_{kd}^2\right] + 2\mathrm{E}\left[g_{is}g_{js}g_{ks}g_{kd}\right] + 2\mathrm{E}\left[g_{is}g_{jd}g_{ks}g_{kd}\right] + 2\mathrm{E}\left[g_{id}g_{js}g_{ks}g_{kd}\right] + 2\mathrm{E}\left[g_{id}g_{jd}g_{ks}g_{kd}\right]$$

$$\mathrm{E}\left[g_{is}g_{js}g_{ks}^2\right] = p\left(S_{is,js,ks}=1\right)\{(1-p)(0-p)^4 + p(1-p)^4\} + p\left(S_{is,js,ks}=2\right)\{(1-p)^2(0-p)^4 + 2p(1-p)(1-p)^2(0-p)^2 + p^2(1-p)^4\} = p\left(S_{is,js,ks}=1\right)p(1-p)[1-3p(1-p)] + p\left(S_{is,js,ks}=2\right)[p(1-p)]^2$$

$$\mathrm{E}\left[g_{is}g_{js}g_{ks}g_{kd}\right] = p\left(S_{is,js,ks,kd}=1\right)p(1-p)[1-3p(1-p)] + \left\{p\left(S_{is,js,ks,kd}=6\right) + p\left(S_{is,js,ks,kd}=9\right) + p\left(S_{is,js,ks,kd}=12\right)\right\}[p(1-p)]^2$$

$$\mathrm{E}\left[X_i X_j X_k^2\right] = p(1-p)[1-3p(1-p)]\left[\textstyle\sum_{a_i\in\{s,d\}}\sum_{a_j\in\{s,d\}}\sum_{a_k\in\{s,d\}} p\left(S_{ia_i,ja_j,ka_k}=1\right) + 2\sum_{a_i\in\{s,d\}}\sum_{a_j\in\{s,d\}} p\left(S_{ia_i,ja_j,ks,kd}=1\right)\right] + [p(1-p)]^2\left\{\textstyle\sum_{a_i\in\{s,d\}}\sum_{a_j\in\{s,d\}}\sum_{a_k\in\{s,d\}} p\left(S_{ia_i,ja_j,ka_k}=2\right) + 2\left[\sum_{a_i\in\{s,d\}}\sum_{a_j\in\{s,d\}} p\left(S_{ia_i,ja_j,ks,kd}=6\right) + p\left(S_{ia_i,ja_j,ks,kd}=9\right) + p\left(S_{ia_i,ja_j,ks,kd}=12\right)\right]\right\}$$

5) $\mathrm{E}\left[X_i X_j X_k X_l\right]\ i \neq j \neq k \neq l$

$$\mathrm{E}\left[X_i X_j X_k X_l\right] = \mathrm{E}\left[(g_{is}+g_{id})(g_{js}+g_{jd})(g_{ks}+g_{kd})(g_{ls}+g_{ld})\right] = \textstyle\sum_{a_i\in\{s,d\}}\sum_{a_j\in\{s,d\}}\sum_{a_k\in\{s,d\}}\sum_{a_l\in\{s,d\}} \mathrm{E}\left[g_{ia_i}g_{ja_j}g_{ka_k}g_{la_l}\right]$$

$$\mathrm{E}\left[g_{is}g_{js}g_{ks}g_{ls}\right] = p\left(S_{is,js,ks,ls} = 1\right)p(1-p)[1-3p(1-p)] + \left\{p\left(S_{is,js,ks,ls} = 6\right) + p\left(S_{is,js,ks,ls} = 9\right) + p\left(S_{is,js,ks,ls} = 12\right)\right\}[p(1-p)]^2$$

$$\boxed{\mathrm{E}\left[X_iX_jX_kX_l\right] = p(1-p)[1-3p(1-p)]\left[\sum_{a_i\in\{s,d\}}\sum_{a_j\in\{s,d\}}\sum_{a_k\in\{s,d\}}\sum_{a_l\in\{s,d\}} p\left(S_{ia_i,ja_j,ka_k,la_l} = 1\right)\right] + [p(1-p)]^2\left[\sum_{a_i\in\{s,d\}}\sum_{a_j\in\{s,d\}}\sum_{a_k\in\{s,d\}}\sum_{a_l\in\{s,d\}}\left\{p\left(S_{ia_i,ja_j,ka_k,la_l} = 6\right) + p\left(S_{ia_i,ja_j,ka_k,la_l} = 9\right) + p\left(S_{ia_i,ja_j,ka_k,la_l} = 12\right)\right\}\right]}$$

Figure SM1-1

Possible IBD states, depending on the number of chromosomes

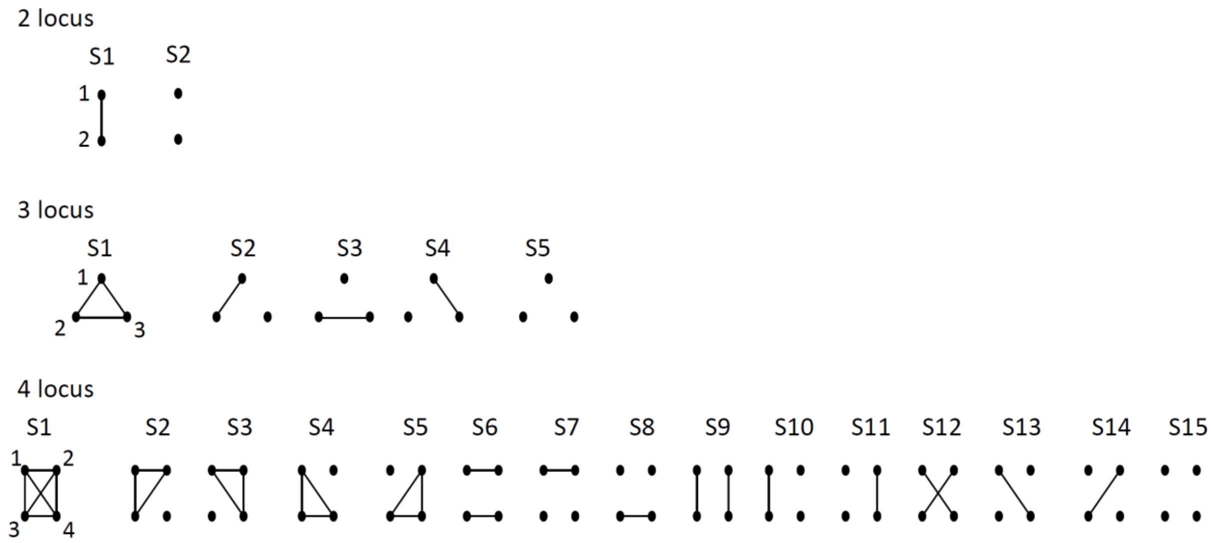

Supplement: Supplementary file 1 — 10.1186/s12711-016-0183-3 Computation of ${\text{E}}\left[ {{\text{X}}_{\text{im}}^{{{\text{d}}_{\text{i}} }} {\text{X}}_{\text{jm}}^{{{\text{d}}_{\text{j}} }} \cdots {\text{X}}_{\text{Km}}^{{{\text{d}}_{\text{K}} }} } \right]$EXimdiXjmdj⋯XKmdK as a function of between-chromosome identity coefficients, in the case of independent markers. Using an extension of the identity coefficients theory, the document shows how to compute the elements of the expectation E[XX′TXX′] (i.e. ${\text{E}}\left[ {X_{i} X_{j} } \right], {\text{E}}\left[ {X_{i} X_{j}^{2} } \right], {\text{E}}\left[ {X_{i} X_{j}^{3} } \right], {\text{E}}\left[ {X_{i}^{2} X_{j}^{2} } \right],$EXiXj,EXiXj2,EXiXj3,EXi2Xj2, ${\text{E}}\left[ {X_{i} X_{j} X_{k}^{2} } \right]$EXiXjXk2 and E[X i X j X k X l]) when markers are independent and individuals are related. [file 12711_2016_183_MOESM1_ESM.pdf]
