# Supplementary material for: Approximated prediction of genomic selection accuracy when reference and candidate populations are related
Source: Genet Sel Evol. 2016 Mar 3;48:18. doi: 10.1186/s12711-016-0183-3 (PMC4778372; doi:10.1186/s12711-016-0183-3)
Supplement: Supplementary file 2 — 10.1186/s12711-016-0183-3 Expectations of $\mathop \sum \limits_{m} \sigma_{m}^{2} , \mathop \sum \limits_{m} \sigma_{m}^{4} , \mathop \sum \limits_{m} \rho_{m}$∑mσm2,∑mσm4,∑mρm, ∑m ρ m2, ∑m ρ m2/σ m2 and ∑m1/σ m2. The expectations of the listed quantities are computed assuming either a U-shaped or a uniform distribution of allele frequencies. [file 12711_2016_183_MOESM2_ESM.pdf]

Additional file 2. Expectations of $\sum_m \sigma_m^2$, $\sum_m \sigma_m^4$, $\sum_\mathrm{m} \rho_\mathrm{m}$, $\sum_\mathrm{m} \rho_\mathrm{m}^2$ $\sum_\mathrm{m} \rho_\mathrm{m}^2/\sigma_\mathrm{m}^2$ and $\sum_\mathrm{m} 1/\sigma_\mathrm{m}^2$

The elements were defined as $\tau = \sum_m \sigma_m^2$ and $\tau_2 = \sum_m \sigma_m^4$, $\rho_m = \frac{n_r\sigma_m^2}{\lambda_\beta + n_r\sigma_m^2}$ and $\sigma_m^2 = 2p_m(1-p_m)$. The parameter $\lambda_\beta = \sigma_e^2/\sigma_\beta^2 = n_M \frac{\overline{\sigma_\mathrm{m}^2}\sigma_e^2}{\sigma_\mathrm{q}^2}$. Notations will be simplified forgetting the m indice.

For the U shaped distribution, following Goddard [16], the allele frequencies p_m were assumed to be distributed with a density $f(p) = k/2\mathrm{p}(1-\mathrm{p})$ with $k = 1/\log 2N_e$, and N_e the effective size of the reference population.

The demonstration largely uses decomposition in simple elements:

$\lambda_\beta + n_r\sigma_m^2 = 2n_r[h + p(1-p)]$ and $h + p(1-p) = -(p-\beta_1)(p-\beta_2)$

With $h = \frac{\lambda_\beta}{2n_r}$, $\beta_1 = \frac{1-\omega}{2}$, $\beta_2 = \frac{1+\omega}{2}$ and $\omega = \sqrt{1+4h}$ (note that $\beta_1 + \beta_2 = 1$, $\beta_1 - \beta_2 = -\omega$, $\beta_1\beta_2 = -h$)

The decompositions are $\frac{1}{h+p(1-p)} = -\frac{1}{(\beta_1-\beta_2)}\left[\frac{1}{(p-\beta_1)} - \frac{1}{(p-\beta_2)}\right]$ and $\frac{1}{[(p-\beta_1)(p-\beta_2)]^2} = \frac{1}{(\beta_1-\beta_2)^3}\left[-\frac{2}{p-\beta_1} + \frac{\beta_1-\beta_2}{(p-\beta_1)^2} + \frac{2}{p-\beta_2} + \frac{\beta_1-\beta_2}{(p-\beta_2)^2}\right]$ giving

$$\int \frac{1}{h+p(1-p)}dp = \frac{1}{(\beta_1-\beta_2)}\left[\log\left(\frac{p-\beta_2}{p-\beta_1}\right)\right] = A \quad [1]$$

$$\int \frac{1}{[h+p(1-p)]^2}dp = \frac{1}{(\beta_1-\beta_2)^3}\left[2\log\left(\frac{p-\beta_2}{p-\beta_1}\right) - (\beta_1-\beta_2)\left(\frac{1}{p-\beta_1} + \frac{1}{p-\beta_2}\right)\right] = B \quad [2]$$

1) $\mathrm{E}[\sigma^2] = \mathrm{E}[2p(1-p)]$

a- U shaped distribution

$\mathrm{E}[\sigma^2] = \int_{1/N_e}^{1-1/N_e} 2p(1-p)\,f(p)dp = k(1-2/N_e)$ following [16] about integration bounds.

When N_e is large: $\mathrm{E}[\sigma^2] = k$

b- Uniform distribution

$$\mathrm{E}[\sigma^2] = \int_0^1 2p(1-p)\,u(p)dp = 2\left[\frac{p^2}{2}\right]_0^1 - 2\left[\frac{p^3}{3}\right]_0^1 = \frac{1}{3}$$

2) $\mathrm{E}[\sigma^4] = \mathrm{E}[[2p(1-p)]^2]$

a- U shaped distribution

$$\mathrm{E}[\sigma^4] = \int_{1/N_e}^{1-1/N_e}[2p(1-p)]^2\,f(p)dp = 2k\int_{1/N_e}^{1-1/N_e} p(1-p)dp = 2k\left[\frac{1}{6} - \frac{1}{N_e^2} + \frac{2}{3N_e^3}\right]$$

When N_e is large: $\mathrm{E}[\sigma^4] = k/3$

b- Uniform distribution

$$\mathrm{E}[\sigma^4] = \int_0^1 [2p(1-p)]^2\, u(p)dp = 4\left(\left[\frac{p^3}{3}\right]_0^1 - 2\left[\frac{p^4}{4}\right]_0^1 + \left[\frac{p^5}{5}\right]_0^1\right) = \frac{2}{15}$$

3) $\mathrm{E}[\rho] = \mathrm{E}\left[\frac{n_r\sigma^2}{\lambda_\beta + n_r\sigma^2}\right] = \mathrm{E}\left[\frac{p(1-p)}{h+p(1-p)}\right]$

a- U shaped distribution

$$\mathrm{E}[\rho] = \mathrm{E}\left[\frac{n_r\sigma^2}{\lambda_\beta + n_r\sigma^2}\right] = \mathrm{E}\left[\frac{p(1-p)}{h+p(1-p)}\right] = \int_{1/N_e}^{1-1/N_e} \frac{p(1-p)}{h+p(1-p)} f(p)dp \ = \frac{k}{2}\int_{1/N_e}^{1-1/N_e} \frac{1}{h+p(1-p)} dp$$

Using [1], $\mathrm{E}[\rho] = \frac{k}{2(\beta_1-\beta_2)}\log\left(\frac{(1-1/N_e-\beta_2)}{(1-1/N_e-\beta_1)} \times \frac{(1/N_e-\beta_1)}{(1/N_e-\beta_2)}\right)$

When N_e is large: $\mathrm{E}[\rho] = \frac{k}{-2\omega}\log\left(\left(\frac{1-\omega}{1+\omega}\right)^2\right) = \frac{k}{\omega}\log\left(\left|\frac{1+\omega}{1-\omega}\right|\right) = \frac{k}{\omega}\theta$

b- Uniform distribution

$$\mathrm{E}[\rho] = \int_0^1 \frac{p(1-p)}{h+p(1-p)} dp$$

From [1] , $\int \frac{p(1-p)}{h+p(1-p)} dp = 1 - hA$ and $\mathrm{E}[\rho] = 1 - h\frac{1}{(\beta_1-\beta_2)}\left[\log\left(\frac{1-\beta_2}{1-\beta_1} \times \frac{\beta_1}{\beta_2}\right)\right]$

$$\mathrm{E}[\rho] = 1 - \frac{h}{-\omega}\log\left(\left(\frac{1-\omega}{1+\omega}\right)^2\right) = 1 - \frac{2h}{\omega}\theta$$

4) $\mathrm{E}[\rho^2]$

a- U shaped distribution

$$\mathrm{E}[\rho^2] = \int_{1/N_e}^{1-1/N_e} \left(\frac{p(1-p)}{h+p(1-p)}\right)^2 f(p)dp = \frac{k}{2}\int_{1/N_e}^{1-1/N_e} \frac{p(1-p)}{(h+p(1-p))^2} dp$$

From [1] and [2], $\int \frac{p(1-p)}{(h+p(1-p))^2} dp = \mathrm{A} - hB$

$$\frac{2}{k}\mathrm{E}[\rho^2] = \frac{1}{(\beta_1-\beta_2)}\left[\log\left(\frac{p-\beta_2}{p-\beta_1}\right)\right]_{1/N_e}^{1-1/N_e} - h\left\{\frac{1}{(\beta_1-\beta_2)^3}\left[2\log\left(\frac{p-\beta_2}{p-\beta_1}\right) - (\beta_1-\beta_2)\left(\frac{1}{p-\beta_1} + \frac{1}{p-\beta_2}\right)\right]_{1/N_e}^{1-1/N_e}\right\}$$

We used $\left[\log\left(\frac{p-\beta_2}{p-\beta_1}\right)\right]_{1/N_e}^{1-1/N_e} = \log\left(\frac{(1-1/N_e-\beta_2)}{(1-1/N_e-\beta_1)} \times \frac{(1/N_e-\beta_1)}{(1/N_e-\beta_2)}\right) \sim \log\left(\left(\frac{\beta_1}{\beta_2}\right)^2\right) = -2\theta$

and $\left[\left(\frac{1}{p-\beta_1}\right)\right]_{1/N_e}^{1-1/N_e} = \frac{2/N_e - 1}{(1-1/N_e-\beta_1)(1/N_e-\beta_1)} \sim \frac{1}{\beta_1\beta_2} = -\frac{1}{h}$

$$\frac{2}{k}\mathrm{E}[\rho^2] = \frac{1}{(\beta_1-\beta_2)}(-2\theta) - h\left\{\frac{1}{(\beta_1-\beta_2)^3}\left[2(-2\theta) - (\beta_1-\beta_2)2\left(-\frac{1}{h}\right)\right]\right\}$$

$$\mathrm{E}[\rho^2] = \frac{k}{2}\left(2\theta\left[-\frac{1}{(\beta_1-\beta_2)} + \frac{2h}{(\beta_1-\beta_2)^3}\right] - \frac{2}{(\beta_1-\beta_2)^2}\right) = -\frac{k}{\omega^2} + \frac{k\theta}{\omega}\left[1 - \frac{2h}{\omega^2}\right] = \frac{k}{\omega^2}\left[\theta\left(\omega - \frac{2h}{\omega}\right) - 1\right]$$

b- Uniform distribution

$$\mathrm{E}[\rho^2] = \int_0^1 \left(\frac{p(1-p)}{h+p(1-p)}\right)^2 dp = \int_0^1 \left(1 - \frac{2hp(1-p)}{\left(h+p(1-p)\right)^2} + \frac{h^2}{\left(h+p(1-p)\right)^2}\right)dp$$

From [1] and [2], $\mathrm{E}[\rho^2] = 1 - 2h(\mathrm{A} - hB) + h^2B = (1+h)^2B - 2hA$

$$\mathrm{E}[\rho^2] = (1+h)^2\left\{\frac{1}{(\beta_1-\beta_2)^3}\left[2\log\left(\frac{p-\beta_2}{p-\beta_1}\right) - (\beta_1-\beta_2)\left(\frac{1}{p-\beta_1}+\frac{1}{p-\beta_2}\right)\right]_0^1\right\} - 2h\frac{1}{(\beta_1-\beta_2)}\left[\log\left(\frac{p-\beta_2}{p-\beta_1}\right)\right]_0^1$$

We used $\left[\log\left(\frac{p-\beta_2}{p-\beta_1}\right)\right]_0^1 \sim -2\theta$ and $\left[\left(\frac{1}{p-\beta_1}\right)\right]_0^1 = -\frac{1}{h}$

$$\mathrm{E}[\rho^2] = (1+h)^2\left\{\frac{1}{(\beta_1-\beta_2)^3}\left[-4\theta - (\beta_1-\beta_2)\left(-\frac{2}{h}\right)\right]\right\} - 2h\frac{1}{(\beta_1-\beta_2)}[-2\theta] = 4\theta\left(\frac{(1+h)^2}{\omega^3} - \frac{h}{\omega}\right) + 2\left(\frac{(1+h)^2}{h\omega^2}\right) = \frac{4\theta}{\omega}\left(\left(\frac{1+h}{\omega}\right)^2 - \mathrm{h}\right) + \frac{2}{h}\left(\frac{1+h}{\omega}\right)^2 = \left(\frac{4\theta}{\omega} + \frac{2}{h}\right)\left(\frac{1+h}{1+4h}\right)^2 - \frac{4\theta h}{\omega}$$

5) $\mathrm{E}[\rho^2/\sigma^2]$

a- U shaped distribution

$$\mathrm{E}[\rho^2/\sigma^2] = \int_{1/N_e}^{1-1/N_e}\frac{\left(\frac{p(1-p)}{h+p(1-p)}\right)^2}{2p(1-p)}f(p)dp = \frac{k}{4}\int_{1/N_e}^{1-1/N_e}\left(\frac{1}{h+p(1-p)}\right)^2 dp$$

From [2] $\mathrm{E}[\rho^2/\sigma^2] = \frac{k}{4}\frac{1}{(\beta_1-\beta_2)^3}\left[2\log\left(\frac{p-\beta_2}{p-\beta_1}\right) - (\beta_1-\beta_2)\left(\frac{1}{p-\beta_1}+\frac{1}{p-\beta_2}\right)\right]_{1/N_e}^{1-1/N_e}$

When N_e is large and using $\left[\log\left(\frac{p-\beta_2}{p-\beta_1}\right)\right]_0^1 \sim -2\theta$ and $\left[\left(\frac{1}{p-\beta_1}\right)\right]_0^1 = -\frac{1}{h}$

$$\mathrm{E}[\rho^2/\sigma^2] = \frac{k}{4}\frac{1}{(\beta_1-\beta_2)^3}\left[-4\theta - (\beta_1-\beta_2)\left(-\frac{2}{h}\right)\right] = \frac{k}{2h\omega^2} + \frac{k\theta}{\omega^3} = \frac{k}{2\omega^3}\left[\frac{\omega}{h} + 2\theta\right]$$

b- Uniform distribution

$\mathrm{E}[\rho^2/\sigma^2] = \int_0^1\frac{\left(\frac{p(1-p)}{h+p(1-p)}\right)^2}{2p(1-p)}dp = \frac{1}{2}\int_0^1\frac{p(1-p)}{(h+p(1-p))^2}dp$ which was found in the $\mathrm{E}[\rho^2]$ for the U-shaped distribution.

Thus $\mathrm{E}[\rho^2/\sigma^2] = \frac{1}{\omega^2}\left[\theta\left(\omega - \frac{2h}{\omega}\right) - 1\right]$

6) $\mathrm{E}[1/\sigma^2]$

a- U shaped distribution

$$\mathrm{E}[1/\sigma^2] = \int_{1/N_e}^{1-1/N_e}\frac{1}{2p(1-p)}f(p)dp = \frac{k}{4}\int_{1/N_e}^{1-1/N_e}\left(\frac{1}{p(1-p)}\right)^2 dp = \frac{k}{4}\int_{1/N_e}^{1-1/N_e}\left(\frac{1}{p^2} + \frac{1}{(1-p)^2} + \frac{2}{p} + \frac{2}{1-p}\right)dp$$

Thus $\mathrm{E}[1/\sigma^2] = \frac{k}{4}\left[2\log\left(\frac{p}{1-p}\right) - \frac{1}{p} + \frac{1}{1-p}\right]_{1/N_e}^{1-1/N_e} = \frac{k}{4}\left[2\log(N_e-1) + 2\frac{N_e(N_e-2)}{N_e-1}\right]$

b- Uniform distribution

Keeping the $1/N_e$, $1-1/N_e$ bounds, $\mathrm{E}[1/\sigma^2] = \int_{1/N_e}^{1-1/N_e}\frac{1}{2p(1-p)}dp = \log(N_e-1)$
