# Supplementary material for: Approximated prediction of genomic selection accuracy when reference and candidate populations are related
Source: Genet Sel Evol. 2016 Mar 3;48:18. doi: 10.1186/s12711-016-0183-3 (PMC4778372; doi:10.1186/s12711-016-0183-3)
Supplement: Supplementary file 3 — 10.1186/s12711-016-0183-3 Precision formulae when the candidate is related to reference individuals. The approximated formulae derived in the main text are applied to the case of a candidate for which the sire belongs to the reference population. [file 12711_2016_183_MOESM3_ESM.pdf]

Additional file 3. Precisions formulae when the candidate is related to reference individuals

First approximation

Matrices $\mathbf{A}$ and $\mathbf{T}$ needed to apply formula $\{I\}$ are the following:

The pedigree relationship matrix is $\mathbf{A} = \begin{pmatrix} 1 & 1/2 & 0 & \cdots & 0 \\ 1/2 & 1 & 0 & \cdots & 0 \\ 0 & 0 & 1 & \cdots & 0 \\ \vdots & \vdots & \vdots & \ddots & \vdots \\ 0 & 0 & 0 & \cdots & 1 \end{pmatrix}$ and

$\mathbf{T} = \begin{pmatrix} a & b & 0 & \cdots & 0 \\ b & a & 0 & \cdots & 0 \\ 0 & 0 & 1/(1+\gamma) & \cdots & 0 \\ \vdots & \vdots & \vdots & \ddots & \vdots \\ 0 & 0 & 0 & \cdots & 1/(1+\gamma) \end{pmatrix}$ with $\gamma = \frac{\sigma_q^2}{\sigma_e^2}$, $a = \frac{1+\gamma}{(1+\gamma)^2 - 1/4\gamma^2}$ and $b = -\frac{\gamma/2}{(1+\gamma)^2 - 1/4\gamma^2}$

$\{\mathbf{TATAT}\}_{\mathrm{cc}} = \mathrm{a}[(a+b/2)^2 + (b+a/2)^2] + \mathrm{b}[2(a+b/2)(b+a/2)]$

$\{\mathbf{T}\mathrm{E}[\mathbf{G}^*\mathbf{T}\mathbf{G}^*]\mathbf{T}\}_{\mathrm{cc}} = a^2\mathrm{h}_{\mathrm{cc}} + 2\mathrm{abh}_{\mathrm{c1}} + b^2\mathrm{h}_{11}$, with $\mathrm{h} = \mathrm{E}[\mathbf{G}^*\mathbf{T}\mathbf{G}^*]$

$\{\mathrm{E}[\mathbf{G}^*\mathbf{T}\mathbf{G}^*]\}_{\mathrm{ij}} = \sum_l \sum_k t_{kl}\left(\frac{1}{2}\tau\alpha_{ijkl}^{1111} - \frac{1}{4}\tau_2\gamma_{ijkl}^{1111} + 4a_{ik}a_{jl}[\tau^2 - \tau_2]\right)$

$\mathrm{h}_{\mathrm{cc}} = \sum_l \sum_k t_{kl}\left(\frac{1}{2}\tau\alpha_{ckl}^{211} - \frac{1}{4}\tau_2\gamma_{ckl}^{211} + 4a_{ck}a_{cl}[\tau^2 - \tau_2]\right)$

Considering the structure of $\mathbf{T}$ matrix in this case:

$\mathrm{h}_{\mathrm{cc}} = t_{cc}\left(\frac{1}{2}\tau\alpha_c^4 - \frac{1}{4}\tau_2\gamma_c^4 + 4a_{cc}^2[\tau^2 - \tau_2]\right) + t_{11}\left(\frac{1}{2}\tau\alpha_{c1}^{22} - \frac{1}{4}\tau_2\gamma_{c1}^{22} + 4a_{c1}^2[\tau^2 - \tau_2]\right) +$
$\sum_{k\neq c\&1} t_{kk}\left(\frac{1}{2}\tau\alpha_{ck}^{22} - \frac{1}{4}\tau_2\gamma_{ck}^{22} + 4a_{ck}^2[\tau^2 - \tau_2]\right) + 2t_{c1}\left(\frac{1}{2}\tau\alpha_{c1}^{31} - \frac{1}{4}\tau_2\gamma_{c1}^{31} + 4a_{cc}a_{c1}[\tau^2 - \tau_2]\right)$

The needed parameters, following table 1, are given in Supplementary table 3.

Supplementary table 3

Parameters corresponding to the situation of a candidate son of a reference individuals ($k \neq c$ & 1)

α_c^4	γ_c^4	α_{c1}^{22}	γ_{c1}^{22}	α_{c1}^{31}	γ_{c1}^{31}	α_{ck}^{22}	γ_{ck}^{22}	α_{ck}^{31}	γ_{ck}^{31}	α_{ckl}^{211}	γ_{ckl}^{211}	a_{cc}	a_{c1}	a_{ck}
2	0	1	0	1	0	0	-4	0	0	0	-1	1/2	1/4	0

Thus $\mathrm{h}_{\mathrm{cc}} = \mathrm{a}(\tau + \tau^2 - \tau_2) + \mathrm{a}\left(\frac{1}{2}\tau + \frac{1}{4}[\tau^2 - \tau_2]\right) + \frac{\mathrm{n_r}-1}{1+\lambda}\tau_2 + 2\mathrm{b}\left(\frac{1}{2}\tau + \frac{1}{2}[\tau^2 - \tau_2]\right)$. I will note $\tau + \tau^2 - \tau_2 = \gamma_1$ and $\frac{1}{2}\tau + \frac{1}{4}[\tau^2 - \tau_2] = \gamma_2$

$$\Rightarrow \mathrm{h}_{\mathrm{cc}} = (\mathrm{a}+\mathrm{b})(\tau + \tau^2 - \tau_2) + \mathrm{a}\left(\frac{1}{2}\tau + \frac{1}{4}[\tau^2 - \tau_2]\right) + \frac{\mathrm{n_r} - 1}{1+\gamma}\tau_2$$

For symmetrical reasons, the diagonal term h_{11} is identical to h_{cc}

$\mathrm{h}_{\mathrm{c1}} = \sum_l \sum_k t_{kl}\left(\frac{1}{2}\tau\alpha_{c1kl}^{1111} - \frac{1}{4}\tau_2\gamma_{c1kl}^{1111} + 4a_{ck}a_{1l}[\tau^2 - \tau_2]\right)$

$\mathrm{h_{c1}} = t_{cc}\left(\frac{1}{2}\tau\alpha_{c1}^{31} - \frac{1}{4}\tau_2\gamma_{c1}^{31} + 4a_{cc}a_{c1}[\tau^2 - \tau_2]\right) + t_{11}\left(\frac{1}{2}\tau\alpha_{1c}^{31} - \frac{1}{4}\tau_2\gamma_{1c}^{31} + 4a_{11}a_{c1}[\tau^2 - \tau_2]\right) + \sum_{k\neq c\&1} t_{kk}\left(\frac{1}{2}\tau\alpha_{kc1}^{211} - \frac{1}{4}\tau_2\gamma_{kc1}^{211} + 4a_{ck}a_{1k}[\tau^2 - \tau_2]\right) + t_{1c}\left(\frac{1}{2}\tau\alpha_{1c}^{22} - \frac{1}{4}\tau_2\gamma_{1c}^{22} + 4a_{c1}^2[\tau^2 - \tau_2]\right) + t_{c1}\left(\frac{1}{2}\tau\alpha_{1c}^{22} - \frac{1}{4}\tau_2\gamma_{1c}^{22} + 4a_{cc}a_{11}[\tau^2 - \tau_2]\right)$

$\mathrm{h_{c1}} = a\left(\frac{1}{2}\tau + \frac{1}{2}[\tau^2 - \tau_2]\right) + a\left(\frac{1}{2}\tau + \frac{1}{2}[\tau^2 - \tau_2]\right) + \frac{\mathrm{n_r}-1}{1+\gamma}\left(\frac{1}{4}\tau_2\right) + b\left(\frac{1}{2}\tau + \frac{1}{4}[\tau^2 - \tau_2]\right) + b\left(\frac{1}{2}\tau + [\tau^2 - \tau_2]\right)$

$$\Rightarrow \mathrm{h_{c1}} = (\mathrm{a}+\mathrm{b})(\tau + \tau^2 - \tau_2) + \tfrac{1}{4}\mathrm{b}(\tau^2 - \tau_2) + \frac{\mathrm{n_r}-1}{1+\gamma}\left(\frac{1}{4}\tau_2\right)$$

For symmetrical reasons, the $\mathrm{h_{1c}}$ is identical to $\mathrm{h_{c1}}$

$\{\mathbf{T}\mathrm{E}[\mathbf{G}^*\mathbf{T}\mathbf{G}^*]\mathbf{T}\}_{\mathrm{cc}} = \mathrm{a^2h_{cc}} + 2\mathrm{abh_{c1}} + \mathrm{b^2h_{11}} = (\mathrm{a}+\mathrm{b})^3(\tau + \tau^2 - \tau_2) + (\mathrm{a^2}+\mathrm{b^2})\mathrm{a}\left(\frac{1}{2}\tau + \frac{1}{4}[\tau^2 - \tau_2]\right) + \frac{1}{2}\mathrm{ab^2}(\tau^2 - \tau_2) + \frac{\mathrm{n_r}-1}{1+\gamma}\tau_2\left[\mathrm{a^2} + \mathrm{b^2} + \frac{1}{2}ab\right]$

Using $\sigma_q^2\mathrm{E}[\mathbf{\Lambda}_{\mathrm{cc}}] = \gamma\mathbf{T}_{\mathrm{cc}} - \gamma^3\{\mathbf{TATAT}\}_{\mathrm{cc}} + \frac{\gamma^3}{\tau^2}\{\mathbf{T}\mathrm{E}[\mathbf{G}^*\mathbf{T}\mathbf{G}^*]\mathbf{T}\}_{\mathrm{cc}}$ and after some algebra the precision comes to be

$$\widetilde{\mathrm{E}}\left[r_{q_{\mathrm{c}},\hat{q}_{\mathrm{c}}}^2\right] \sim \frac{1}{\nu^2} - \frac{1}{\gamma\mathrm{a} + \gamma^3\frac{\tau - \tau_2}{\tau^2}\left[(\mathrm{a}+\mathrm{b})^3 + (\mathrm{a^2}+\mathrm{b^2})\frac{1}{2}\mathrm{a}\right] + \gamma^3\frac{\tau_2}{\tau^2}\left[\frac{1}{4}\mathrm{a}(\mathrm{b^2}-\mathrm{a^2}) + \frac{\mathrm{n_r}-1}{1+\gamma}\left(\mathrm{a^2}+\mathrm{b^2}+\frac{1}{2}\,\mathrm{ab}\right)\right]} \qquad [SM3.1]$$

Second approximation

With the hypothesis of relatedness between reference individuals and absence of inbreeding, the averaged coefficients in formula $\{III\}$ are $\overline{a_{ci}^2} = \frac{1}{\mathrm{n_r}}\frac{1}{16}$, $\overline{\alpha_{ci}^{22}} = \frac{1}{\mathrm{n_r}}$ and $\overline{\gamma_{ci}^{22}} = -4\frac{\mathrm{n_r}-1}{\mathrm{n_r}}$.

$$\widetilde{\widetilde{\mathrm{E}}}\left[r_{q_{\mathrm{c}},\hat{q}_{\mathrm{c}}}^2\right] = 1 - \lambda_\beta\frac{\left(\sum_m\rho_m\right) + \left(\sum_m\rho_m^2\right)\left(1 + \frac{1}{4\mathrm{n_r}} - \frac{\mathrm{n_r}-1}{\mathrm{n_r}}\right) - \left(\sum_m\rho_m\right)^2\frac{1}{4\mathrm{n_r}} - \left(\sum_m\frac{\rho_m^2}{\sigma_m^2}\right)\left(\frac{1}{2}\frac{1}{\mathrm{n_r}}\right)}{n_r\tau}$$

Expectations of elements in ρ_m are given in table 2.

$$\widetilde{\widetilde{\mathrm{E}}}\left[r_{q_{\mathrm{c}},\hat{q}_{\mathrm{c}}}^2\right] = 1 - \lambda_\beta\mathrm{n_M}\frac{\frac{k}{\omega}\theta + \frac{5}{4\mathrm{n_r}}\frac{k}{\omega^2}\left[\theta\left(\omega - \frac{2h}{\omega}\right) - 1\right] - \frac{1}{4\mathrm{n_r}}\mathrm{n_M}\left(\frac{k}{\omega}\theta\right)^2 - \frac{1}{2\mathrm{n_r}}\left(\frac{k}{2\omega^3}\left\{2\theta + \frac{\omega}{h}\right\}\right)}{\tau\mathrm{n_r}}$$

Using $\lambda_\beta = \tau/\gamma$, and after some algebra,

$$\widetilde{\widetilde{\mathrm{E}}}\left[r_{q_{\mathrm{c}},\hat{q}_{\mathrm{c}}}^2\right] = 1 - \frac{\mathrm{n_M}}{\gamma n_r^2}\left[\mathrm{n_r}\frac{k}{\omega}\theta + \frac{5}{4}\frac{k}{\omega^2}\left[\theta\left(\omega - \frac{2h}{\omega}\right) - 1\right] - \frac{1}{4}\mathrm{n_M}\left(\frac{k}{\omega}\theta\right)^2 - \frac{k}{4\omega^3}\left\{2\theta + \frac{\omega}{h}\right\}\right]$$

$$\widetilde{\widetilde{\mathrm{E}}}\left[r_{q_{\mathrm{c}},\hat{q}_{\mathrm{c}}}^2\right] = 1 - \frac{\mathrm{n_M}k\theta}{\gamma\mathrm{n_r}\omega} - \frac{\mathrm{n_M}k}{4\gamma n_r^2\omega^2}\left(5\theta\omega - \frac{10h\theta}{\omega} - \frac{2\theta}{\omega} - 5 - \mathrm{n_M}\mathrm{k}\theta^2 - \frac{1}{h}\right) \qquad [SM3.2]$$
