# Supplementary material for: Approximated prediction of genomic selection accuracy when reference and candidate populations are related
Source: Genet Sel Evol. 2016 Mar 3;48:18. doi: 10.1186/s12711-016-0183-3 (PMC4778372; doi:10.1186/s12711-016-0183-3)
Supplement: Supplementary file 4 — 10.1186/s12711-016-0183-3 Equivalent numbers of independent loci. (1) equation of the equivalent number of independent loci which gives the precision ${\text{E}}\left[ {r_{{q_{\text{c}} ,\hat{q}_{c} }}^{2} } \right]\sim \frac{{{\text{E}}_{{\mathbf{X}}} \left[ {{\text{v}}(\hat{q}_{c} |{\mathbf{X}})} \right]}}{{{\text{E}}_{{\mathbf{X}}} \left[ {{\text{v}}\left( {q_{\text{c}} |{\mathbf{X}}} \right)} \right]}}$Erqc,q^c2∼EXv(q^c|X)EXvqc|X obtained with the total number of non-independent markers; (2) simple approximation in a very simplified situation; (3) relations between number of independent markers and size of the reference population. [file 12711_2016_183_MOESM4_ESM.pdf]

Additional file 4. Equivalent numbers of independent loci

Estimation of equivalent number of independent markers

Let $\mathbf{x}_\mathbf{c}^\mathbf{i}$ and $\mathbf{X}_\mathbf{r}^\mathbf{i}$ be the genotype matrices of the independent loci. We search the equivalent number of independent loci which gives the precision $\mathrm{E}\left[r^2_{q_c,\hat{q}_c}\right] \sim \frac{\mathrm{E}_\mathbf{X}[\mathrm{v}(\hat{q}_c|\mathbf{X})]}{\mathrm{E}_\mathbf{X}[\mathrm{v}(q_c|\mathbf{X})]}$ obtained with the total number of non-independent markers. It will be supposed that $\mathrm{E}_\mathbf{X}[\mathrm{v}(q_c|\mathbf{X})] = \mathrm{E}[\mathrm{v}(q_c)] = \sigma^2_\mathrm{q}$ is the same considering all markers or only the set of independent loci. This may be a critical hypothesis if the correlation between the molecular score (q_c) and the genetic value (g_c) is not high. Under this hypothesis, $\sigma^2_\mathrm{q} = \tau\sigma^2_\beta = \tau_i\sigma^2_{\mathrm{i}\beta}$ and the ratios $\lambda_\beta = \frac{\tau}{\gamma}$ and $\lambda^\mathrm{i}_\beta = \frac{\tau_i}{\gamma}$.

The equivalence will be observed if $\mathrm{E}_{\mathbf{x}_\mathbf{c},\mathbf{X}_\mathbf{r}}[v(\hat{q}_c|\mathbf{x}_\mathbf{c},\mathbf{X}_\mathbf{r})] = \mathrm{E}_{\mathbf{x}^\mathrm{i}_\mathbf{c},\mathbf{X}^\mathrm{i}_\mathbf{r}}\left[v\left(\hat{q}_c|\mathbf{x}^\mathbf{i}_\mathbf{c},\mathbf{X}^\mathbf{i}_\mathbf{r}\right)\right]$. Using $v(\hat{q}_c|\mathbf{x}_\mathbf{c},\mathbf{X}_\mathbf{r}) = \sigma^2_\beta\mathbf{x}_\mathbf{c}\mathbf{x}'_\mathbf{c} - \sigma^2_\mathrm{e}\mathbf{x}_\mathbf{c}\left(\mathbf{X}'_\mathbf{r}\mathbf{X}_\mathbf{r} + \lambda_\beta\mathbf{I}\right)^{-1}\mathbf{x}'_\mathbf{c}$ and assuming that $\mathbf{X}'_\mathbf{r}\mathbf{X}_\mathbf{r} \sim \mathrm{E}[\mathbf{X}'_\mathbf{r}\mathbf{X}_\mathbf{r}]$ we have

$$\mathrm{E}_{\mathbf{x}_\mathbf{c},\mathbf{X}_\mathbf{r}}[v(\hat{q}_c|\mathbf{x}_\mathbf{c},\mathbf{X}_\mathbf{r})] = \sigma^2_\beta\mathrm{E}[\mathbf{x}'_\mathbf{c}\mathbf{x}_\mathbf{c}] - \sigma^2_\mathrm{e}tr\left[\left(\mathrm{E}[\mathbf{X}'_\mathbf{r}\mathbf{X}_\mathbf{r}] + \lambda_\beta\mathbf{I}\right)^{-1}\mathrm{E}[\mathbf{x}'_\mathbf{c}\mathbf{x}_\mathbf{c}]\right]$$

If $\mathrm{E}[\mathbf{x}'_\mathbf{c}\mathbf{x}_\mathbf{c}] = \mathrm{E}[\mathbf{X}'_\mathbf{r}\mathbf{X}_\mathbf{r}]$, the trace becomes $tr[\mathrm{I}] - \lambda_\beta tr\left[\left(\mathrm{E}[\mathbf{X}'_\mathbf{r}\mathbf{X}_\mathbf{r}] + \lambda_\beta\mathbf{I}\right)^{-1}\right]$

These relations hold for the independent loci case and the equivalence supposes that

$$-n_M\sigma^2_\mathrm{e} + \lambda_\beta\sigma^2_e tr\left[\left(\mathrm{E}[\mathbf{X}'_\mathbf{r}\mathbf{X}_\mathbf{r}] + \lambda_\beta\mathbf{I}\right)^{-1}\right] = -n_{M_i}\sigma^2_\mathrm{e} + \lambda^\mathrm{i}_\beta\sigma^2_\mathrm{e} tr\left[\left(\mathrm{E}\left[\mathbf{X}^{\mathbf{i}\prime}_\mathbf{r}\mathbf{X}^\mathbf{i}_\mathbf{r}\right] + \lambda^\mathrm{i}_\beta\mathbf{I}\right)^{-1}\right] \qquad [SM4.1]$$

The expectations matrices depend on marker alleles frequencies and linkage disequilibrium:

$$\mathrm{E}\left[\mathbf{X}^{\mathbf{i}\prime}_\mathbf{r}\mathbf{X}^\mathbf{i}_\mathbf{r}\right] = n_R\begin{pmatrix} s^2_1 & \cdots & 0 \\ \vdots & \ddots & \vdots \\ 0 & \cdots & s^2_{n_{M_i}}\end{pmatrix} \text{ and } \mathrm{E}[\mathbf{X}'_\mathbf{r}\mathbf{X}_\mathbf{r}] = n_R\begin{pmatrix} \sigma^2_1 & \Delta_{12} & \cdots & \Delta_{1n_M} \\ \Delta_{21} & \sigma^2_2 & \cdots & \Delta_{2n_M} \\ \vdots & \vdots & \ddots & \vdots \\ \Delta_{n_M1} & \Delta_{n_M2} & \cdots & \sigma^2_{n_M}\end{pmatrix}$$

If we assume all genotypic values variances equal in the independent loci case, *i.e.* $s^2_m = s^2$, it comes that $\tau_i = n_{M_i}s^2$ and $tr\left[\left(\mathrm{E}\left[\mathbf{X}^{\mathbf{i}\prime}_\mathbf{r}\mathbf{X}^\mathbf{i}_\mathbf{r}\right] + \lambda^\mathrm{i}_\beta\mathbf{I}\right)^{-1}\right] = \frac{n_{M_i}}{n_R s^2 + \lambda^\mathrm{i}_\beta} = \frac{1}{s^2}\frac{\gamma n_{M_i}}{\gamma n_R + n_{M_i}}$. The right hand side of $[SM4.1]$ is

$$-n_{M_i}\sigma^2_\mathrm{e} + \frac{n_{M_i}s^2}{\gamma}\sigma^2_\mathrm{e}\frac{1}{s^2}\frac{\gamma n_{M_i}}{\gamma n_R + n_{M_i}} = -n_{M_i}\sigma^2_\mathrm{e}\left(1 - \frac{n_{M_i}}{\gamma n_R + n_{M_i}}\right) = -n_{M_i}\sigma^2_\mathrm{e}\frac{\gamma n_R}{\gamma n_R + n_{M_i}}$$

Let $\mathrm{T} = tr\left[\left(\mathrm{E}[\mathbf{X}'_\mathbf{r}\mathbf{X}_\mathbf{r}] + \lambda_\beta\mathbf{I}\right)^{-1}\right]$, the equivalence gives $-n_M + \lambda_\beta T = -n_{M_i}\frac{\gamma n_R}{\gamma n_R + n_{M_i}}$

Thus $(-n_M + \lambda_\beta\mathrm{T})(\gamma n_R) = n_{M_i}(-\gamma n_R + n_M - \lambda_\beta\mathrm{T})$, and using $\tau = \sum_m \sigma^2_m = n_M\overline{\sigma^2_\mathrm{m}}$, we get

$$n_{M_i} = \frac{\left(\overline{\sigma^2_\mathrm{m}}\mathrm{T} - \gamma\right)(\gamma n_R)}{\left(\gamma - \overline{\sigma^2_\mathrm{m}}\mathrm{T} - \gamma^2 n_R/n_M\right)} \qquad [SM4.2]$$

In models predicting genetic gain expected from a genomic selection scheme applied to a real population, one of the parameters needed is the equivalent number of loci which can be estimated from formula $[SM4.2]$ given marker alleles frequencies and between markers linkage disequilibrium observed in the population.

A very simple approximation for n_{M_e} is obtained assuming equal variances $\sigma_m^2 = s^2$, and using relation between expected linkage disequilibrium and effective population size N_e as derived by [32] $\mathrm{E}[2\Delta_{ml}] = \sigma_m \sigma_l / \sqrt{1 + 4N_e d_{lm}}$ with d_{lm} the distance between ordered locus l and m, such that $d_{lm} = |l - m|L/n_M$, with L the genome length in Morgans. With those hypotheses, $\mathrm{T} = tr\left[\left(n_R s^2 \mathbf{R} + \lambda_\beta \mathbf{I}\right)^{-1}\right]$ with $\{\mathbf{R}\}_{\mathrm{ml}} = \mathrm{r}_{\mathrm{ml}} = \sqrt{n_M/(n_M + 4N_e|l - m|L)}$.

As $\lambda_\beta = \frac{n_M s^2}{\gamma}$ we have $\mathrm{T} = \frac{\gamma}{s^2} tr[(\gamma n_R \mathbf{R} + n_M \mathbf{I})^{-1}] = \frac{\gamma}{s^2}\mathrm{U}$

In this simplified situation, the equivalent number of loci is

$$n_{M_i} = n_M \frac{n_R \gamma (1 - \mathrm{U})}{n_R \gamma - n_M (1 - \mathrm{U})} \qquad [SM4.3]$$

Relation between trace T and the reference population size n_R.

The objective is to understand how the number of independent markers n_{M_i} varies with n_R.

From $[SM4.2]$, the derivative

$$\frac{dn_{M_i}}{dT} \propto \overline{\sigma_{\mathrm{m}}^2}(\gamma n_R)\left(\gamma - \overline{\sigma_{\mathrm{m}}^2}\mathrm{T} - \frac{\gamma^2 n_R}{n_M}\right) - \overline{\sigma_{\mathrm{m}}^2}\left(\overline{\sigma_{\mathrm{m}}^2}\mathrm{T} - \gamma\right)(\gamma n_R) = -\overline{\sigma_{\mathrm{m}}^2}\gamma^3 \frac{{n_R}^2}{n_M} < 0$$

Thus, the n_{M_i} number decreases when the trace T increases.

In the simplified situation, $\mathrm{T} = tr\left[\left(n_R s^2 \mathbf{R} + \lambda_\beta \mathbf{I}\right)^{-1}\right]$ with $\mathrm{r}_{\mathrm{ml}} = \sqrt{n_M/(n_M + 4N_e|l - m|L)}$.

We consider the simplest situation of 2 markers. In this case, $\mathrm{T} = \frac{2(n_R s^2 + \lambda_\beta)}{(n_R s^2 + \lambda_\beta)^2 - (n_R s^2 \mathrm{r}_{12})^2}$ showing that (i) the trace under linkage disequilibrium is larger than the trace for independent markers ($\mathrm{T}_{LD} > \mathrm{T}_{LE}$), (ii) the derivative of T_{LE} is negative ($\frac{d\mathrm{T}_{LE}}{dn_R} \propto -2s^2$) meaning that with larger n_R, T_{LE} is lower and n_{M_i} higher and (iii) the derivative of ratio $\mathrm{T}_{LE}/\mathrm{T}_{LD} = 1 - \frac{(n_R s^2 \mathrm{r}_{12})^2}{(n_R s^2 + \lambda_\beta)^2}$ is negative ($\frac{d\mathrm{T}_{LE}/\mathrm{T}_{LD}}{dn_R} = -2\frac{n_R s^4 \mathrm{r}_{12} \lambda_\beta}{(n_R s^2 + \lambda_\beta)^3}$) showing that the difference between those traces decreases when n_R becomes larger.

Globally, these information indicate that with larger reference population sizes, larger number of independent loci (figures closer to the number of markers in LD) are needed to obtain the same reliability.
