# Supplementary material for: Approximated prediction of genomic selection accuracy when reference and candidate populations are related
Source: Genet Sel Evol. 2016 Mar 3;48:18. doi: 10.1186/s12711-016-0183-3 (PMC4778372; doi:10.1186/s12711-016-0183-3)
Supplement: Supplementary file 5 — 10.1186/s12711-016-0183-3 The case of markers in linkage disequilibrium. Derivation of the crossed terms expectation of genomic values (E[x cl x cm X il X im]) in the situation of LD between loci l and m. Many examples are given for diverse situations. [file 12711_2016_183_MOESM5_ESM.pdf]

Additional file 5. The case of markers in Linkage disequilibrium

Principles

The objective is to derive $\mathrm{E}[x_{cl}x_{cm}X_{il}X_{im}]$ in the situation of LD. As in Supplementary Material 1, we use $X_{im} = g_{imf} + g_{imd}$ where g_{imf} and g_{imd} are the "values" of the alleles transmitted to individual i by its father and its dam, with g_{imf} and $g_{imd} = (0\ or\ 1) - p_m$. Equivalent terms are defined for x_{cl} , x_{cm} and X_{il}.

$$\mathrm{E}[x_{cl}x_{cm}X_{il}X_{im}] = \sum_{s\in\{f,d\}}\sum_{t\in\{f,d\}}\sum_{u\in\{f,d\}}\sum_{v\in\{f,d\}} \mathrm{E}[g_{cls}g_{cmt}g_{ilu}g_{imv}]$$

The random variable M_{cls} is the allele individual c received from s at locus l. M_{cmt}, M_{ilu} and M_{imv} are defined similarly. More generally, $M_{\alpha lf}$ and are the locus l allele individual α received from its father and $M_{\alpha ld}$, from its dam.

Four types of $g = (g_{cls}, g_{cmt}, g_{ilu,}g_{imv})$ vectors are encountered

$$\mathcal{T}_{s=t}^{u=v} : \ s = t\ and\ \ u = v$$

$$\mathcal{T}_{s=t}^{u\neq v} : s = t\ and\ \ u \neq v$$

$$\mathcal{T}_{s\neq t}^{u=v} : s \neq t\ and\ \ u = v$$

$$\mathcal{T}_{s\neq t}^{u\neq v} : s \neq t\ and\ \ u \neq v$$

As each of the indices s, t, u, v may be $f$or d, a total of 16 different g vectors are possible.

In type 1, both alleles (belonging to locus $m\ and\ l$) of each couple of locus (one for c and one for i) are on the same chromosome (may be from the two fathers, the two dams, c's father and i's dam or i's father and c's dam).

In type 2, both alleles (belonging to locus $m\ and\ l$) of the candidate c are on the same chromosome, while alleles of the reference i are not on the same chromosome.

Type 3 is the reverse from type 2.

In type 4, alleles of locus $m\ and\ l$ of both individuals c and i are on different chromosomes.

For each of these situations, we consider the IBD status between alleles at locus m on chromosomes ct and iv, and at locus l on chromosomes cs and iu.

We may have

$\mathcal{S}_{ml} = \{\ M_{cmt} \equiv M_{imv}\ \ and\ \ M_{cls} \equiv M_{ilu}\}$ with a probability φ_{ml}^{stuv}

$\mathcal{S}_{m\not{l}} = \{\ M_{cmt} \equiv M_{imv}\ \ and\ \ M_{cls} \not\equiv M_{ilu}\}$ with a probability $\varphi_{m\not{l}}^{stuv}$

$\mathcal{S}_{\not{m}l} = \{\ M_{cmt} \not\equiv M_{imv}\ \ and\ \ M_{cls} \equiv M_{ilu}\}$ with a probability $\varphi_{\not{m}l}^{stuv}$

$\mathcal{S}_{\not{m}\not{l}} = \{\ M_{cmt} \not\equiv M_{imv}\ \ and\ \ M_{cls} \not\equiv M_{ilu}\}$ with a probability $\varphi_{\not{m}\not{l}}^{stuv}$

The computation of the probability φ_k^{stuv} depends on the type of g -vector.

$$\mathrm{E}[g_{cls}g_{cmt}g_{ilu}g_{imv}] = \sum_{k\in\{ml,m\cancel{l},\cancel{m}l,\cancel{ml}\}} \varphi_k^{stuv}\mathrm{E}[g_{cls}g_{cmt}g_{ilu}g_{imv}|\mathcal{S}_k]$$

Expectations $\mathrm{E}[\boldsymbol{g_{cls}g_{cmt}g_{ilu}g_{imv}}|\boldsymbol{\mathcal{S}_k}]$

Let $p_{AA} = (1-p_m)(1-p_l) + \Delta_{lm}$; $p_{AB} = (1-p_m)p_l - \Delta_{lm}$; $p_{BA} = p_m(1-p_l) - \Delta_{lm}$ and $p_{BB} = p_m p_l + \Delta_{lm}$. Under $\mathcal{S}_{ml}$, only 4 genotypes vectors are possible :

				$\mathrm{prob}[g_{cls}g_{cmt}g_{ilu}g_{imv}\|\mathcal{S}_{ml}]$				
M_{cls}	M_{cmt}	M_{ilu}	M_{imv}	$s=t \; and \; u=v$	$s=t \; and \; u\neq v$	$s\neq t \; and \; u=v$	$s\neq t \; and \; u\neq v$	$g_{cls}g_{cmt}g_{ilu}g_{imv}$
A_l	A_m	A_l	A_m	p_{AA}	p_{AA}	p_{AA}	$(1-p_m)(1-p_l)$	$p_m^2 p_l^2$
A_l	B_m	A_l	B_m	p_{BA}	p_{BA}	p_{BA}	$p_m(1-p_l)$	$p_l^2(1-p_m)^2$
B_l	A_m	B_l	A_m	p_{AB}	p_{AB}	p_{AB}	$(1-p_m)p_l$	$p_m^2(1-p_l)^2$
B_l	B_m	B_l	B_m	p_{BB}	p_{BB}	p_{BB}	$p_m p_l$	$(1-p_l)^2(1-p_m)^2$

The expectation follows:

	$\mathrm{E}[g_{cls}g_{cmt}g_{ilu}g_{imv}\|\mathcal{S}_{ml}]$
$s=t \; and \; u=v$	$(1-p_m)(1-p_l)p_m p_l + \Delta_{lm}(1-2p_l)(1-2p_m)$
$s=t \; and \; u\neq v$	$(1-p_m)(1-p_l)p_m p_l + \Delta_{lm}(1-2p_l)(1-2p_m)$
$s\neq t \; and \; u=v$	$(1-p_m)(1-p_l)p_m p_l + \Delta_{lm}(1-2p_l)(1-2p_m)$
$s\neq t \; and \; u\neq v$	$(1-p_m)(1-p_l)p_m p_l$

For other IBD status ($\mathcal{S}_{m\cancel{l}}, \mathcal{S}_{\cancel{m}l}, \mathcal{S}_{\cancel{ml}}$), it must be noted that all expectations of the product $g_{cls}g_{cmt}g_{ilu}g_{imv}$ are null in g-types different from $\mathcal{T}_{s=t}^{u=v}$. For instance , given $\mathcal{R} = \{\mathcal{S}_{m\cancel{l}} \; ; \; s = t \; and \; u \neq v\}$, $\mathrm{E}[g_{cls}g_{cmt}g_{ilu}g_{imv}|\mathcal{R}] = \mathrm{E}[g_{cls}g_{cmt}g_{ilu} \times \mathrm{E}[g_{imv}|\mathcal{R}, g_{cls}, g_{cmt}, g_{ilu}]|\mathcal{R}]$.

But $\mathrm{E}[g_{imv}|\mathcal{R}, g_{cls}, g_{cmt}, g_{ilu}] = \mathrm{E}[g_{imv}] = 0$ (the variable g_{imv} is independent on the other g under $\mathcal{R}$).

Genotypes				Genotypes probability for IBD status:			Value of
M_{cls}	M_{cmt}	M_{ilu}	M_{imv}	$\mathcal{S}_{m\cancel{l}}$	$\mathcal{S}_{\cancel{m}l}$	$\mathcal{S}_{\cancel{ml}}$	$g_{cls}g_{cmt}g_{ilu}g_{imv}$
A_l	A_m	A_l	A_m	${p_{AA}}^2/[1-p_m]$	${p_{AA}}^2/[1-p_l]$	${p_{AA}}^2$	$p_m^2 p_l^2$
A_l	A_m	A_l	B_m	0	$p_{AA}p_{BA}/[1-p_l]$	$p_{AA}p_{BA}$	$-p_l^2 p_m(1-p_m)$
A_l	A_m	B_l	A_m	$p_{AA}p_{AB}/[1-p_m]$	0	$p_{AA}p_{AB}$	$-p_m^2 p_l(1-p_l)$
A_l	A_m	B_l	B_m	0	0	$p_{AA}p_{BB}$	$p_m(1-p_m)p_l(1-p_l)$
A_l	B_m	A_l	A_m	0	$p_{BA}p_{AA}/[1-p_l]$	$p_{BA}p_{AA}$	$-p_l^2 p_m(1-p_m)$
A_l	B_m	A_l	B_m	${p_{BA}}^2/p_m$	${p_{BA}}^2/[1-p_l]$	${p_{BA}}^2$	$p_l^2(1-p_m)^2$
A_l	B_m	B_l	A_m	0	0	$p_{BA}p_{AB}$	$p_m(1-p_m)p_l(1-p_l)$
A_l	B_m	B_l	B_m	$p_{BA}p_{BB}/p_m$	0	$p_{BA}p_{BB}$	$-(1-p_m)^2 p_l(1-p_l)$
B_l	A_m	A_l	A_m	$p_{AB}p_{AA}/[1-p_m]$	0	$p_{AB}p_{AA}$	$-p_m^2 p_l(1-p_l)$
B_l	A_m	A_l	B_m	0	0	$p_{AB}p_{BA}$	$p_m(1-p_m)p_l(1-p_l)$
B_l	A_m	B_l	A_m	${p_{AB}}^2/[1-p_m]$	${p_{AB}}^2/p_l$	${p_{AB}}^2$	$p_m^2(1-p_l)^2$
B_l	A_m	B_l	B_m	0	$p_{AB}p_{BB}/p_l$	$p_{AB}p_{BB}$	$-(1-p_l)^2 p_m(1-p_m)$
B_l	B_m	A_l	A_m	0	0	$p_{BB}p_{AA}$	$p_m(1-p_m)p_l(1-p_l)$
B_l	B_m	A_l	B_m	$p_{BB}p_{BA}/p_m$	0	$p_{BB}p_{BA}$	$-(1-p_m)^2 p_l(1-p_l)$
B_l	B_m	B_l	A_m	0	$p_{BB}p_{AB}/p_l$	$p_{BB}p_{AB}$	$-(1-p_l)^2 p_m(1-p_m)$
B_l	B_m	B_l	B_m	${p_{BB}}^2/p_m$	${p_{BB}}^2/p_l$	${p_{BB}}^2$	$(1-p_l)^2(1-p_m)^2$

	$\mathrm{E}[g_{cls}g_{cmt}g_{ilu}g_{imv} \mid s = t \text{ and } u = v]$
$\mathcal{S}_{m\cancel{l}}$	$\Delta_{lm}^2 \times [p_m^3 + (1-p_m)^3]/[p_m(1-p_m]$
$\mathcal{S}_{\cancel{m}l}$	$\Delta_{lm}^2 \times [p_l^3 + (1-p_l)^3]/[p_l(1-p_l]$
$\mathcal{S}_{\cancel{m}\cancel{l}}$	$\Delta_{lm}^2 \times (1-2p_m)(1-2p_l)$

Assembling all elements we get

$$\mathrm{E}[x_{cl}x_{cm}X_{il}X_{im}] = [(1-p_m)(1-p_l)p_m p_l + \Delta_{lm}(1-2p_l)(1-2p_m)][(\sum_{s\in\{f,d\}}\sum_{t\in\{f,d\}}\sum_{u\in\{f,d\}}\sum_{v\in\{f,d\}}\varphi_{ml}^{stuv}) - (\varphi_{ml}^{fdfd} + \varphi_{ml}^{fddf} + \varphi_{ml}^{dffd} + \varphi_{ml}^{dfdf})] + \Delta_{lm}^2 \sum_{s\in\{f,d\}}\sum_{u\in\{f,d\}}[\varphi_{m\cancel{l}}^{ssuu} \times [p_m^3 + (1-p_m)^3]/[p_m(1-p_m] + \varphi_{\cancel{m}l}^{ssuu} \times [p_l^3 + (1-p_l)^3]/[p_l(1-p_l] + \varphi_{\cancel{m}\cancel{l}}^{ssuu} \times (1-2p_m)(1-2p_l)]$$

Probabilities of IBD status $\mathcal{S}_k$

We are interested in genes at loci l and m individuals i and c received from specific parents denoted by s and u or t and v.

The probability $p(M_{cmt} \equiv M_{imv}) = \varphi_m^{tv}$ (*resp.* $p(M_{cls} \equiv M_{ilu}) = \varphi_l^{su}$) equals the coancestry coefficient between t and v (*resp.* s and u). It is estimated the usual way.

We only need to develop $\varphi_{ml}^{stuv} = p(\mathcal{S}_{ml}) = p(M_{cmt} \equiv M_{imv} \text{ and } M_{cls} \equiv M_{ilu})$. Indeed, the other terms are given by:

$$\varphi_{m\cancel{l}}^{stuv} = p(M_{cmt} \equiv M_{imv}) - p(M_{cmt} \equiv M_{imv} \text{ and } M_{cls} \equiv M_{ilu}) = \varphi_m^{tv} - \varphi_{ml}^{stuv}$$

$$\varphi_{\cancel{m}l}^{stuv} = p(M_{cls} \equiv M_{ilu}) - p(M_{cmt} \equiv M_{imv} \text{ and } M_{cls} \equiv M_{ilu}) = \varphi_l^{su} - \varphi_{ml}^{stuv}$$

$$\varphi_{\cancel{m}\cancel{l}}^{stuv} = 1 - (\varphi_{ml}^{stuv} + \varphi_{m\cancel{l}}^{stuv} + \varphi_{\cancel{m}l}^{stuv}) = 1 - (\varphi_m^{tv} + \varphi_l^{su}) + \varphi_{ml}^{stuv}$$

We first examine the situation of a single locus (say l). Given the pedigree, different genealogical chains linking c and i are often possible. They will be indexed $pl = 1 \cdots Nl_p$ (and $pm = 1 \cdots Nm_p$ when considering the m locus). A chain is characterized by a shared ancestor a_{pl} and consists of two subchains (pl_c and pl_i) linking a_{pl} to c and to i . In graph theory, these subchains are made of edges with nodes (the individuals) linked by arcs (characterizing transmission events). Subchain pl_c (resp. pl_i) comprises one and only one parent of c (resp. i). The ancestor may be c or i themselves, in which case the chain is extended to one parent on this ancestor. The lengths (numbers of generations) of the pl_c and pl_i subchains will be noted $n_{a_{pl}c}$ and $n_{a_{pl}i}$. We will note $\alpha = \{\alpha_1, \alpha_2 \cdots \alpha_{n_{a_{pl}c-1}}\}$ and $\beta = \{\beta_1, \beta_2 \cdots \beta_{n_{a_{pl}i-1}}\}$ the lists of intermediate individuals belonging to $pl_c = \{c, \alpha, a_{pl}\}$ and $pl_i = \{i, \beta, a_{pl}\}$. We are interested in the ancestor allele $M_{a_{pl}lwl}$ shared by individuals c and to i . Genders (f or d) of individual ω will be noted $\mathcal{G}(\omega)$.

The IBD status $M_{cls} \equiv M_{ilu}$ occurs if the allele at locus l carried by chromosomes transmitted by s to c and transmitted by u to i both come from the same ancestor allele carried by a_{pl} : $M_{cls} \equiv M_{ilu}$ if

$$\begin{cases} s = \mathcal{G}(\alpha_1) \text{ and } u = \mathcal{G}(\beta_1) \\ M_{cls} \equiv M_{\alpha_1 l \mathcal{G}(\alpha_2)} \equiv M_{\alpha_2 l \mathcal{G}(\alpha_3)} \cdots \equiv M_{a_{pl}lw} \text{ and } M_{ilu} \equiv M_{\beta_1 l \mathcal{G}(\beta_2)} \equiv M_{\beta_2 l \mathcal{G}(\beta_3)} \cdots \equiv M_{a_{pl}lwl} \text{ with } wl = f \text{ or } d \end{cases}$$

In other words the $M_{a_{pl}lwl}$ allele was transmitted :

- along the pl_c subchain to c via s (event $\mathcal{I}^l_{pl_c,wl} = \left\{M_{cls} \equiv M_{\alpha_1 l\mathcal{G}(\alpha_2)} \equiv M_{\alpha_2 l\mathcal{G}(\alpha_3)} \cdots \equiv M_{a_p lw}\right\}$ and
- along the pl_i subchain to i via u (event $\mathcal{I}^l_{pl_i,wl} = \left\{M_{ilu} \equiv M_{\beta_1 l\mathcal{G}(\beta_2)} \equiv M_{\beta_2 l\mathcal{G}(\beta_3)} \cdots \equiv M_{a_{pl}lwl}\right\}$

$$prob(\mathcal{I}^l_{pl_c,wl}) = prob(M_{cls} \equiv M_{\alpha_1 l\mathcal{G}(\alpha_2)})prob(M_{\alpha_1 l\mathcal{G}(\alpha_2)} \equiv M_{\alpha_2 l\mathcal{G}(\alpha_3)} | M_{cls} \equiv M_{\alpha_1 l\mathcal{G}(\alpha_2)}) \cdots$$

Transmissions events between generations being independent, for a single locus (l in the current example), $prob(\mathcal{I}^l_{pl_c,wl}) = \frac{1}{2}^{n_{a_{pl}c}}$ and $prob(\mathcal{I}^l_{pl_i,wl}) = \frac{1}{2}^{n_{a_{pl}i}}$.

It must be noted that pl_c and pl_i may share edges. In these situations $prob(\mathcal{I}^l_{pl_c,wl} \cap \mathcal{I}^l_{pl_i,wl}) = prob(\mathcal{I}^l_{pl_c,wl})prob(\mathcal{I}^l_{pl_i,wl}|\mathcal{I}^l_{pl_c,wl}) \neq prob(\mathcal{I}^l_{pl_c,wl})prob(\mathcal{I}^l_{pl_i,wl})$: If n_s edges belong to both pl_c and pl_i, $prob\left(\mathcal{I}^l_{pl_i,wl}\middle|\mathcal{I}^l_{pl_c,wl}\right) = \frac{1}{2}^{n_{a_{pl}i}-n_s}$. Thus $p(M_{cls} \equiv M_{ilu}|pl, wl) = \frac{1}{2}^{n_{a_{pl}c}+n_{a_{pl}i}-n_s}$

As there is two possible ancestral alleles ($M_{a_{pl}lwl}$ with $wl = f$ or d) shared by chromosomes transmitted by s to c and transmitted by u to i, we finally get the classical $p(M_{cls} \equiv M_{ilu}|pl) = \frac{1}{2}^{n_{a_{pl}c}+n_{a_{pl}i}-1-n_s}$. IBD status $M_{cls} \equiv M_{ilu}$ due to the transmission by the different possible chains being exclusive, the unconditional probability is $p(M_{cls} \equiv M_{ilu}) = \sum_{pl} \frac{1}{2}^{n_{a_{pl}c}+n_{a_{pl}i}-1-n_s}$

When considering both loci l and m simultaneously, all possible pairs (pl, pm) of genealogical chains and parental origins of ancestor alleles linking c and i must be examined.

$$\varphi^{stuv}_{ml} = p(\, M_{cmt} \equiv M_{imv} \; and \; M_{cls} \equiv M_{ilu}) = \sum_{pl,wl} \sum_{pm,wm} p(\, M_{cmt} \equiv M_{imv} \; and \; M_{cls} \equiv M_{ilu}|pl, wl, pm, wm)$$

$$\varphi^{stuv}_{ml} = \sum_{pl,wl} \sum_{pm,wm} p(\, M_{cls} \equiv M_{ilu}|pl, wl)p\left(\, M_{cmt} \equiv M_{imv}|\, M_{cls} \equiv M_{ilu}, pl, wl, pm, wm\right)$$

Previous definitions are extended to pm chain: to pm corresponds ancestor a_{pm}, vectors γ and δ are counterparts of α and β and events $\mathcal{I}^m_{pm_c,wm}$ and $\mathcal{I}^m_{pm_i,wm}$ of events $\mathcal{I}^l_{pl_c,wl}$and $\mathcal{I}^l_{pl_i,wl}$. We have $M_{cmt} \equiv M_{imv}$ if

$$\begin{cases} t = \mathcal{G}(\gamma_1) \text{ and } v = \mathcal{G}(\delta_1) \\ M_{cmt} \equiv M_{\gamma_1 m\mathcal{G}(\gamma_2)} \equiv M_{\gamma_2 m\mathcal{G}(\gamma_3)} \cdots \equiv M_{a_{pm}mw} \text{ and } M_{imv} \equiv M_{\delta_1 m\mathcal{G}(\delta_2)} \equiv M_{\delta_2 m\mathcal{G}(\delta_3)} \cdots \equiv M_{a_{pm}mw} \text{ with } w = f \text{ or } d \end{cases}$$

$$p(\, M_{cmt} \equiv M_{imv}|\, M_{cls} \equiv M_{ilu}, pl, wl, pm, wm) = p\left(\mathcal{I}^m_{pm_c,wm} \cap \mathcal{I}^m_{pm_i,wm}|\mathcal{I}^l_{pl_c,wl} \cap \mathcal{I}^l_{pl_i,wl}\right)$$

The pm chain may be partially confounded with pl chain: an edge (γ_k, γ_{k+1}) from pm may or not be present in pl. If not, $prob(M_{\gamma_k m\mathcal{G}(\gamma_{k+1})} \equiv M_{\gamma_{k+1} m\mathcal{G}(\gamma_{k+2})} |\mathcal{I}^l_{pl_c,wl} \cap \mathcal{I}^l_{pl_i,wl}) = 1/2$. If $(\gamma_k, \gamma_{k+1}) = (\alpha_h, \alpha_{h+1})$ is both in pm and pl, the probability simplifies to $prob(M_{\gamma_k m\mathcal{G}(\gamma_{k+1})} \equiv M_{\gamma_{k+1} m\mathcal{G}(\gamma_{k+2})} \left|M_{\alpha_h l\mathcal{G}(\alpha_{h+1})} \equiv M_{\alpha_{h+1} l\mathcal{G}(\alpha_{h+2})}\right.)$. In this case, either $\gamma_{k+2} = \alpha_{h+2}$ (alleles at locus l and m transmitted by $\gamma_{k+1} = \alpha_{h+1}$ to $\gamma_k = \alpha_h$ were on the same grand parental chromosome), giving $prob(M_{\gamma_k m\mathcal{G}(\gamma_{k+1})} \equiv M_{\gamma_{k+1} m\mathcal{G}(\gamma_{k+2})} \left|M_{\alpha_h l\mathcal{G}(\alpha_{h+1})} \equiv M_{\alpha_{h+1} l\mathcal{G}(\alpha_{h+2})}\right.) = 1 - r_{ml}$, or $\gamma_{k+2} \neq \alpha_{h+2}$, giving $prob(M_{\gamma_k m\mathcal{G}(\gamma_{k+1})} \equiv M_{\gamma_{k+1} m\mathcal{G}(\gamma_{k+2})} \left|M_{\alpha_h l\mathcal{G}(\alpha_{h+1})} \equiv M_{\alpha_{h+1} l\mathcal{G}(\alpha_{h+2})}\right.) = r_{ml}$.

Computing φ^{stuv}_{ml}, all types of $g = (g_{cls}, g_{cmt}, g_{ilu}, g_{imv})$ vectors must be considered ($\mathcal{T}^{u=v}_{s=t}, \mathcal{T}^{u\neq v}_{s=t}, \mathcal{T}^{u=v}_{s\neq t}, \mathcal{T}^{u\neq v}_{s\neq t}$) for all combination of pl and pm chains.

Examples

A. i and c are unrelated

In this case, probability $\varphi^{ssuu}_{\not m\not l} = 1$ is the only non null, and the expectation turns to be:

$$\mathrm{E}[x_{cl}x_{cm}X_{il}X_{im}] = 4\Delta^2_{lm}(1-2p_m)(1-2p_l)$$

B. i is one of $c's$ parents

Two chains are possible: $p_1 = a_{p_1} \to i \to c$ and $p_2 = a_{p_2} \to i \to c$ with a_{p_1} and a_{p_2} , parents of i as ancestors. Here $n_{a_{p1}c} = 2, n_{a_{p1}i} = 1, n_s = 1$.

a. l and m genes on the same chromosome in c and i ($\mathcal{T}^{u=v}_{s=t}$)

Non null probabilities are

$p(\,M_{cls} \equiv M_{ilu}|pl, wl) = \frac{1}{4}$ if $\mathcal{G}(i) = s$ and $\mathcal{G}(a_{pl}) = u$; $\forall wl = f$ or d and $\forall pl = p_1$ or p_2

$p(\,M_{cms} \equiv M_{imu}|M_{cls} \equiv M_{ilu}, pl, wl, pm, wm) = (1-r_{ml})^2$ if $\mathcal{G}(i) = s, \mathcal{G}(a_{pl}) = u$; $pl = pm, wl = wm$

$p(\,M_{cms} \equiv M_{imu}|M_{cls} \equiv M_{ilu}, pl, wl, pm, wm) = r_{ml}(1-r_{ml})$ if $\mathcal{G}(i) = s, \mathcal{G}(a_{pl}) = u$; $pl = pm, wl \neq wm$

Finally, $\varphi^{ssuu}_{ml} = \sum_{pl}\frac{1}{4}\left((1-r_{ml})^2 + r_{ml}(1-r_{ml})\right) = \sum_{pl}\frac{1}{4}(1-r_{ml}) = \frac{1-r_{ml}}{2}$

$\varphi^{ssuu}_{m\not l} = \frac{r_{ml}}{2}$; $\varphi^{ssuu}_{\not m l} = \frac{r_{ml}}{2}$; $\varphi^{ssuu}_{\not m\not l} = \frac{1-r_{ml}}{2}$

b. l and m genes on the same chromosome in c, on two chromosomes in i ($\mathcal{T}^{u\neq v}_{s=t}$)

$p(\,M_{cls} \equiv M_{ilu}|pl, wl) = \frac{1}{4}$ if $\mathcal{G}(i) = s$ and $\mathcal{G}(a_{pl}) = u$; $\forall wl = f$ or d and $\forall pl = p_1$ or p_2

$p(\,M_{cms} \equiv M_{imv}|M_{cls} \equiv M_{ilu}, pl, wl, pm, wm) = p\left(M_{cms} \equiv M_{im\mathcal{G}(a_{pm})}|M_{cls} \equiv M_{il\mathcal{G}(a_{pl})}\right)p\left(M_{im\mathcal{G}(a_{pm})} \equiv M_{a_{pm}mwm}\right) = \frac{1}{2}r_{ml}$ if $\mathcal{G}(i) = s, \mathcal{G}(a_{pl}) = u, \mathcal{G}(a_{pm}) = v$; $pl \neq pm, wl \neq wm$. Indeed, as $u \neq v$, (1) either $pl = p_1$ and $pm = p_2$, or $pl = p_2$ and $pm = p_1$ and (2) either $wl = f$ and $wm = d$, or $wl = d$ and $wm = f$

Finally, $\varphi^{ssuv}_{ml} = \sum_{pl,wl}\frac{1}{8}r_{ml} = \frac{r_{ml}}{2}$

$\varphi^{ssuv}_{m\not l} = \frac{1-r_{ml}}{2}$; $\varphi^{ssuv}_{\not m l} = \frac{1-r_{ml}}{2}$; $\varphi^{ssuv}_{\not m\not l} = \frac{r_{ml}}{2}$

c. l and m genes are on two chromosomes in c ($\mathcal{T}^{u=v}_{s\neq t}$ or $\mathcal{T}^{u\neq v}_{s\neq t}$)

In these cases $p(\,M_{cmt} \equiv M_{imu}|M_{cls} \equiv M_{ilu}, pl, wl, pm, wm) = 0$ since parent i cannot be both c sire and dam.

$\varphi_{ml}^{stuu} = 0 \,;\, \varphi_{m\not{l}}^{stuu} = \frac{1}{2} \,;\, \varphi_{\not{m}l}^{stuu} = \frac{1}{2} \,;\, \varphi_{\not{m}\not{l}}^{stuu} = 0$

$\varphi_{ml}^{stuv} = 0 \,;\, \varphi_{m\not{l}}^{stuv} = \frac{1}{2} \,;\, \varphi_{\not{m}l}^{stuv} = \frac{1}{2} \,;\, \varphi_{\not{m}\not{l}}^{stuv} = 0$

Using these probabilities, the expectation turns to be:

$$\mathrm{E}[x_{cl}x_{cm}X_{il}X_{im}] = (1-p_m)(1-p_l)p_m p_l + \Delta_{lm}(1-2p_l)(1-2p_m) + 2\Delta_{lm}^2\left[r_{ml}\left(\frac{p_m^3+(1-p_m)^3}{p_m(1-p_m)} + \frac{p_l^3+(1-p_l)^3}{p_l(1-p_l)}\right) + (1-r_{ml})(1-2p_m)(1-2p_l)\right]$$

When there is no linkage disequilibrium, $\mathrm{E}[x_{cl}x_{cm}X_{il}X_{im}] = (1-p_m)(1-p_l)p_m p_l = 4\sigma_{\mathrm{m}}^2\sigma_{\mathrm{l}}^2 a_{ci}^2$ as expected.

C. i and c had the same sire

Only one genealogical chain links i and c with subchains $p_c = a_p \to c$ and $p_i = a_p \to i$ with a_p their sire. Here $n_{a_p c} = n_{a_p i} = 1, n_s = 0$. Non null probabilities are obtained only when l and m genes on the same chromosome in c and $(\mathcal{T}_{s=t}^{u=v})$, and when $s = u = f = \mathcal{G}(a_p)$. In this case we have:

$$p(\, M_{cls} \equiv M_{ilu} | p, wl) = p(\, M_{cls} \equiv M_{a_p lwl} \;\&\; M_{ilu} \equiv M_{a_p lwl} \;| p, wl) = \frac{1}{4} \quad \forall wl = f \text{ or } d$$

$$p(\, M_{cms} \equiv M_{imu} | M_{cls} \equiv M_{ilu}, p, wl, wm) = p(\, M_{cms} \equiv M_{a_p mwm} |\, M_{cls} \equiv M_{a_p lwl}\; p, wl, wm)p(\, M_{imu} \equiv M_{a_p mwm} |\, M_{ilu} \equiv M_{a_p lwl}, p, wl, wm) = (1-r_{ml})^2 \quad \text{if} \quad wl = wm \;, r_{ml}^2 \quad \text{if} \quad wl \neq wm$$

Finally, $\varphi_{ml}^{ssuu} = \frac{1}{2}\left((1-r_{ml})^2 + r_{ml}^2\right) = \varphi_{\not{m}\not{l}}^{ssuu}$ and $\varphi_{m\not{l}}^{ssuu} = \varphi_{\not{m}l}^{ssuu} = r_{ml}(1-r_{ml})$

$$\mathrm{E}[x_{cl}x_{cm}X_{il}X_{im}] = \frac{(1-r_{ml})^2+r_{ml}^2}{2}[(1-p_m)(1-p_l)p_m p_l + (\Delta_{lm}+\Delta_{lm}^2)(1-2p_l)(1-2p_m)] + r_{ml}(1-r_{ml})\Delta_{lm}^2\left(\frac{p_m^3+(1-p_m)^3}{p_m(1-p_m)} + \frac{p_l^3+(1-p_l)^3}{p_l(1-p_l)}\right)$$
