# Supplementary material for: Approximated prediction of genomic selection accuracy when reference and candidate populations are related
Source: Genet Sel Evol. 2016 Mar 3;48:18. doi: 10.1186/s12711-016-0183-3 (PMC4778372; doi:10.1186/s12711-016-0183-3)
Supplement: Supplementary file 6 — 10.1186/s12711-016-0183-3 Another demonstration of Goddard et al. [18] accuracy. A complete demonstration is given using the notations of the present paper. [file 12711_2016_183_MOESM6_ESM.pdf]

Additional file 6. Another demonstration of Goddard et al (2011)[18] accuracy

Genotypes are coded using the standardized $x_{im} = (a_{im} - 2p_m)/\sigma_m$. The statistical model is $\mathbf{y} = \mathbf{X\beta} + \mathbf{e}$ and the aim is to predict $\mathrm{g} = \mathbf{W\beta}$. Marker effects are distributed in $\mathcal{L}(\mathbf{0}, \mathbf{I}\sigma_\beta^2)$. The $\mathbf{y}$ distribution conditional to $\mathbf{X}$ is such that $\mathrm{E}(\mathbf{y}|\mathbf{X}) = \mathbf{0}$ and $v(\mathbf{y}|\mathbf{X}) = \mathbf{XX}'\sigma_\beta^2 + \mathbf{I}\sigma_\mathrm{e}^2$. The total phenotypic variance is $v(\mathbf{y}) = \mathrm{v}_\mathbf{X}[\mathrm{E}(\mathrm{y}|\mathbf{X})] + \mathrm{E}_\mathbf{X}[v(\mathrm{y}|\mathbf{X})] = \mathrm{E}_\mathbf{X}[v(\mathrm{y}|\mathbf{X})] = n_M \mathrm{E}[\mathbf{G}]\sigma_\beta^2 + \mathbf{I}\sigma_\mathrm{e}^2$, where $\mathbf{G}$ is the genomic matrix. The markers BLUP is $\widehat{\boldsymbol{\beta}} = (\mathbf{X}'\mathbf{X} + \mathbf{I}\lambda_\beta)^{-1}\mathbf{X}'\mathbf{y} = \mathbf{Py}$ and the GEBVs are $\hat{\mathrm{g}} = \mathbf{WPy} = \mathbf{Sy}$.

Let $\mathbf{PX} = \mathbf{T} = \mathbf{T}'$ giving $\mathbf{P}\left(\mathbf{XX}' + \mathbf{I}\boldsymbol{\lambda}_{\boldsymbol{\beta}}\right)\mathbf{P}' = \mathbf{X}'\mathbf{X}\left(\mathbf{X}'\mathbf{X} + \mathbf{I}\boldsymbol{\lambda}_{\boldsymbol{\beta}}\right)^{-1} = \mathbf{T} = \mathbf{I} - \lambda_\beta(\mathbf{X}'\mathbf{X} + \mathbf{I}\lambda_\beta)^{-1}$

We look for $\mathrm{E}[r^2] = \frac{cov^2(\mathrm{g}, \hat{\mathrm{g}})}{v(\mathrm{g})v(\hat{\mathrm{g}})}$

$$v(\mathrm{g}) = v(\mathbf{W\beta}) = E_\mathbf{w}\left[\mathrm{v}_\beta[\mathbf{W\beta}|\mathbf{W}]\right] + \mathrm{v}_\mathbf{w}\left[\mathrm{E}_\beta[\mathbf{W\beta}|\mathbf{W}]\right] = E_\mathbf{w}\left[\mathrm{v}_\beta[\mathbf{W\beta}|\mathbf{W}]\right] = E_\mathbf{w}\left[\mathbf{WI}\sigma_\beta^2\mathbf{W}'\right]$$

Thus $v(\mathrm{g}) = \mathrm{E}[\mathbf{W}]' \mathbf{I}\sigma_\beta^2 \mathrm{E}[\mathbf{W}] + \mathrm{tr}\left[\mathbf{I}\sigma_\beta^2 \mathrm{v}(\mathbf{W})\right] = \sigma_\beta^2 \sum_{\mathrm{m}=1}^{n_M} \sigma_{\mathrm{wm}}^2$.

$$v(\hat{\mathrm{g}}) = \mathrm{E}_{\mathbf{W},\mathbf{X}}[v(\hat{\mathrm{g}}|\mathbf{W}, \mathbf{X})] + \mathrm{v}_{\mathbf{W},\mathbf{X}}[\mathrm{E}(\hat{\mathrm{g}}|\mathbf{W}, \mathbf{X})] = \mathrm{E}_{\mathbf{W},\mathbf{X}}[v(\hat{\mathrm{g}}|\mathbf{W}, \mathbf{X})]$$

$$\mathrm{v}(\hat{\mathrm{g}}|\mathbf{W}, \mathbf{X}) = \mathbf{WP}\left(\mathbf{XX}'\sigma_\beta^2 + \mathbf{I}\sigma_\mathrm{e}^2\right)\mathbf{P}'\mathbf{W}' = \mathbf{WTW}'\sigma_\beta^2$$

$$\mathrm{E}_{\mathbf{W},\mathbf{X}}[v(\hat{\mathrm{g}}|\mathbf{W}, \mathbf{X})] = \sigma_\beta^2\, \mathrm{E}_\mathbf{X}\left[\mathrm{E}_\mathbf{W}[\mathbf{WTW}'|\mathbf{X}]\right]$$

Let $\varphi_\mathrm{W} = \mathrm{E}[\mathbf{W}|\mathbf{X}]$, we get $\mathrm{E}_{\mathbf{W},\mathbf{X}}[v(\hat{\mathrm{g}}|\mathbf{W}, \mathbf{X})] = \sigma_\beta^2 \mathrm{E}_\mathbf{X}[\varphi_\mathrm{W}\mathbf{T}\varphi_\mathrm{W}' + \mathrm{tr}\{\mathbf{T}v(\mathbf{W}|\mathbf{X})\}]$

$$v(\hat{\mathrm{g}}) = \sigma_\beta^2\left(\mathrm{E}_\mathbf{X}[\varphi_\mathrm{W}\mathbf{T}\varphi_\mathrm{W}'] + \mathrm{E}_\mathbf{X}\left[\mathrm{tr}\{\mathbf{TD}_{\mathbf{W}|\mathbf{X}}\}\right]\right)$$

$$cov(\mathrm{g}, \hat{\mathrm{g}}) = \mathrm{E}_{\mathbf{W},\mathbf{X}}[cov(\mathrm{g}, \hat{\mathrm{g}}|\mathbf{W}, \mathbf{X})] + cov_{\mathbf{W},\mathbf{X}}[\mathrm{E}(\hat{\mathrm{g}}|\mathbf{W}, \mathbf{X}), \mathrm{E}(\mathrm{g}|\mathbf{W}, \mathbf{X})] = \mathrm{E}_{\mathbf{W},\mathbf{X}}[cov(\mathrm{g}, \hat{\mathrm{g}}|\mathbf{W}, \mathbf{X})]$$

$$cov(\mathrm{g}, \hat{\mathrm{g}}|\mathbf{W}, \mathbf{X}) = cov\left(\mathbf{W\beta}, \mathbf{W}\left(\mathbf{X}'\mathbf{X} + \mathbf{I}\lambda_\beta\right)^{-1}\mathbf{X}'(\mathbf{X\beta} + \mathbf{e})|\mathbf{W}, \mathbf{X}\right)$$

$$cov(\mathrm{g}, \hat{\mathrm{g}}|\mathbf{W}, \mathbf{X}) = cov(\mathbf{W\beta}, \mathbf{WT\beta}|\mathbf{W}, \mathbf{X}) = \mathbf{WTW}'\sigma_\beta^2$$

Thus $cov(\mathrm{g}, \hat{\mathrm{g}}) = v(\hat{\mathrm{g}})$

and $\mathrm{E}[r^2] = \frac{v(\hat{\mathrm{g}})}{v(\mathrm{g})} = \frac{\sigma_\beta^2(\mathrm{E}_\mathbf{X}[\varphi_\mathrm{W}\mathbf{T}\varphi_\mathrm{W}'] + \mathrm{E}_\mathbf{X}[\mathrm{tr}\{\mathbf{TD}_{\mathbf{W}|\mathbf{X}}\}])}{\sigma_\beta^2 \sum_{\mathrm{m}=1}^{n_M} \sigma_{\mathrm{wm}}^2}$

If we now suppose that

- Individuals are unrelated $\varphi_\mathrm{W} = \mathrm{E}_\mathbf{W}[\mathbf{W}|\mathbf{X}] = \mathrm{E}_\mathbf{W}[\mathbf{W}] = 0$ et $\mathbf{D}_{\mathbf{W}|\mathbf{X}} = \mathbf{D}_\mathbf{W}$
- Markers are in L.E. $v(\mathbf{W}) = \mathbf{D}_\mathbf{W} = \mathbf{I}$
- $\mathbf{X}'\mathbf{X} \sim \mathrm{E}[\mathbf{X}'\mathbf{X}] = n_R\mathbf{I}$

$\mathrm{E}[r^2] = \frac{\mathrm{E}_\mathbf{X}[\mathrm{tr}\{\mathbf{T}\}]}{n_M}$ with $\mathrm{E}_\mathbf{X}[\mathrm{tr}\{\mathbf{T}\}] = \mathrm{E}_\mathbf{X}\left[n_M - \lambda_\beta \frac{n_M}{n_R + \lambda_\beta}\right] = \frac{n_R n_M}{n_R + \lambda_\beta}$

Finally $\mathrm{E}[r^2] = \frac{n_R}{n_R + \lambda_\beta} = \frac{n_R}{n_R + n_M\lambda} = \frac{\frac{n_R}{n_M}h^2}{\frac{n_R}{n_M}h^2 + 1 - h^2}$
